# Symmetry‐Breaking Triplet Excited State Enhances Red Afterglow Enabling Ubiquitous Afterglow Readout

**DOI:** 10.1002/advs.202308897

**Published:** 2024-02-04

**Authors:** Bahadur Sk, Shuzo Hirata

**Affiliations:** ^1^ Department of Engineering Science The University of Electro‐Communications 1‐5‐1 Chofugaoka, Chofu Tokyo 182‐8585 Japan

**Keywords:** molecular distortion, persistent room temperature phosphorescence, red phosphorescence, symmetry‐breaking, symmetry‐forbidden transition

## Abstract

Molecular vibrations are often factors that deactivate luminescence. However, if there are molecular motion elements that enhance luminescence, it may be possible to utilize molecular movement as a design guideline to enhance luminescence. Here, the authors report a large contribution of symmetry‐breaking molecular motion that enhances red persistent room‐temperature phosphorescence (RTP) in donor‐*π*‐donor conjugated chromophores. The deuterated form of the donor‐*π*‐donor chromophore exhibits efficient red persistent RTP with a yield of 21% and a lifetime of 1.6 s. A dynamic calculation of the phosphorescence rate constant (*k*_p_) indicates that the symmetry‐breaking movement has a crucial role in selectively facilitating *k*_p_ without increasing nonradiative transition from the lowest triplet excited state. Molecules exhibiting efficient red persistent RTP enable long‐wavelength excitation, indicating the suitability of observing afterglow readout in a bright indoor environment with a white‐light‐emitting diode flashlight, greatly expanding the range of anti‐counterfeiting applications that use afterglow.

## Graphical abstract

Organic donor‐*π*‐donor chromophores exhibit a selective enhancement of radiation rate without increasing nonradiative transition from the lowest triplet excited state by symmetry‐breaking molecular motion. This results in a bright red persistent room‐temperature phosphorescence (RTP) yielding 21% and a lifetime of 1.6 s. This enables ubiquitous afterglow readout and expands the range soft anti‐counterfeiting applications.

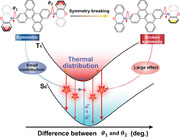

## 1 Introduction

Persistent (lifetime >100 ms) room‐temperature (RT) emission harvesting from metal‐free, heavy‐atom‐free organic chromophores is an active area of research due to diverse imaging advancements.^[^ ^1^ ^]^ This is especially important for bioimaging^[^ ^2^ ^]^ and anti‐counterfeit applications^[^ ^3^ ^]^ as it helps to avoid interference from background autofluorescence. In particular, persistent RT emission at long wavelengths is a crucial material tool for visualization—without interference from autofluorescence—from deep sites in vivo.^[^ ^1^, ^2^, ^3^ ^]^ However, persistent RT emission yield is low in red wavelengths.^[^ ^4^ ^]^ Persistent RT phosphorescence (RTP) from simple (metal‐ and heavy‐atom‐free) organic chromophores enables persistent bright emission compared with long persistent emission via charge separation and recombination.^[^ ^1^, ^5^ ^]^ Accordingly, persistent RTP from simple organic chromophores might increase the resolution of imaging and minimize autofluorescence challenges.^[^ ^6^ ^]^ However, currently, the red persistent RTP yield from simple organic chromophores is <16%.^[^ ^4h^ ^]^ Nevertheless, nearly 100% nonpersistent emission yield at red wavelengths of fluorescent chromophores and metal complexes has been reported.^[^ ^7^ ^]^ Therefore, why simple organic chromophores with highly efficient red persistent RTP have not been reported to date remains unclear. Commonly, to increase the RTP yield, the rate constant of radiation from T_1_ (*k*_p_) should be large compared with the rate constant of the nonradiative transition from T_1_ (*k*_nr_) at RT.^[^ ^8^ ^]^ However, *k*_nr_(RT) increases in a manner that is approximately proportional to the square of the wavelength based on the energy gap law, and *k*_p_ is inversely proportional to the cube of the wavelength.^[^ ^8^ ^]^ Therefore, *k*_p_ > *k*_nr_(RT) is challenging for persistent RTP at long wavelengths (red and near‐infrared). Therefore, discovering key elements of molecular structures and geometries that can selectively enhance *k*_p_ without increasing *k*_nr_(RT) is crucial for enhancing the efficiency of persistent RTP at long wavelengths.

Here we report a substantial contribution of thermally induced symmetry‐breaking of symmetric conjugated chromophores to enhancing *k*_p_ without enhancing *k*_nr_(RT), in a manner that achieved red persistent RTP with a yield of >20%. To evaluate chromophores that exhibit more efficient red afterglow RTP, we selected dibenzo[*g,p*]chrysene (DBC) as a phosphorescent conjugated unit (*π*) and phenoxazine as a planer donating substituted unit (D). Common quantum chemical calculation of *k*_p_ by using T_1_‐optimized structures suggests that the RTP yield of the D‐*π*‐D structure is small compared with the D‐*π* structure. However, the optically determined *k*_p_ of the D‐*π*‐D structure is larger than that of D‐*π*, although the two structures have comparable *k*_nr_(RT). A proposed dynamic quantum chemical calculation—considering the dihedral angle between the D and *π* units, depending on the thermal energy—Figures out that symmetry‐breaking caused by independent changes of two dihedral angles in the D‐*π*‐D structure largely enhanced *k*_p_ without increasing *k*_nr_(RT). Because of the large selective enhancement of *k*_p_ by the symmetry‐breaking, a deuterated compound of the D‐*π*‐D structure indicates red afterglow RTP with a RTP yield of 21% and an RTP lifetime of 1.6 s. We applied films using the high‐efficiency red persistent RTP chromophore to visually observe afterglow in the dark by simply turning off the common room light, and in bright environments by simply illuminating a white‐light‐emitting diode (LED) on a portable phone. This enables ubiquitous afterglow anti‐counterfeiting technology that does not require strong ultraviolet (UV) or deep blue light sources.

## 2 Results and Discussion

### 2.1 Prediction of *k*_nr_(RT) and *k*_p_ by Using a Previously Reported Method

**Figure 1:**
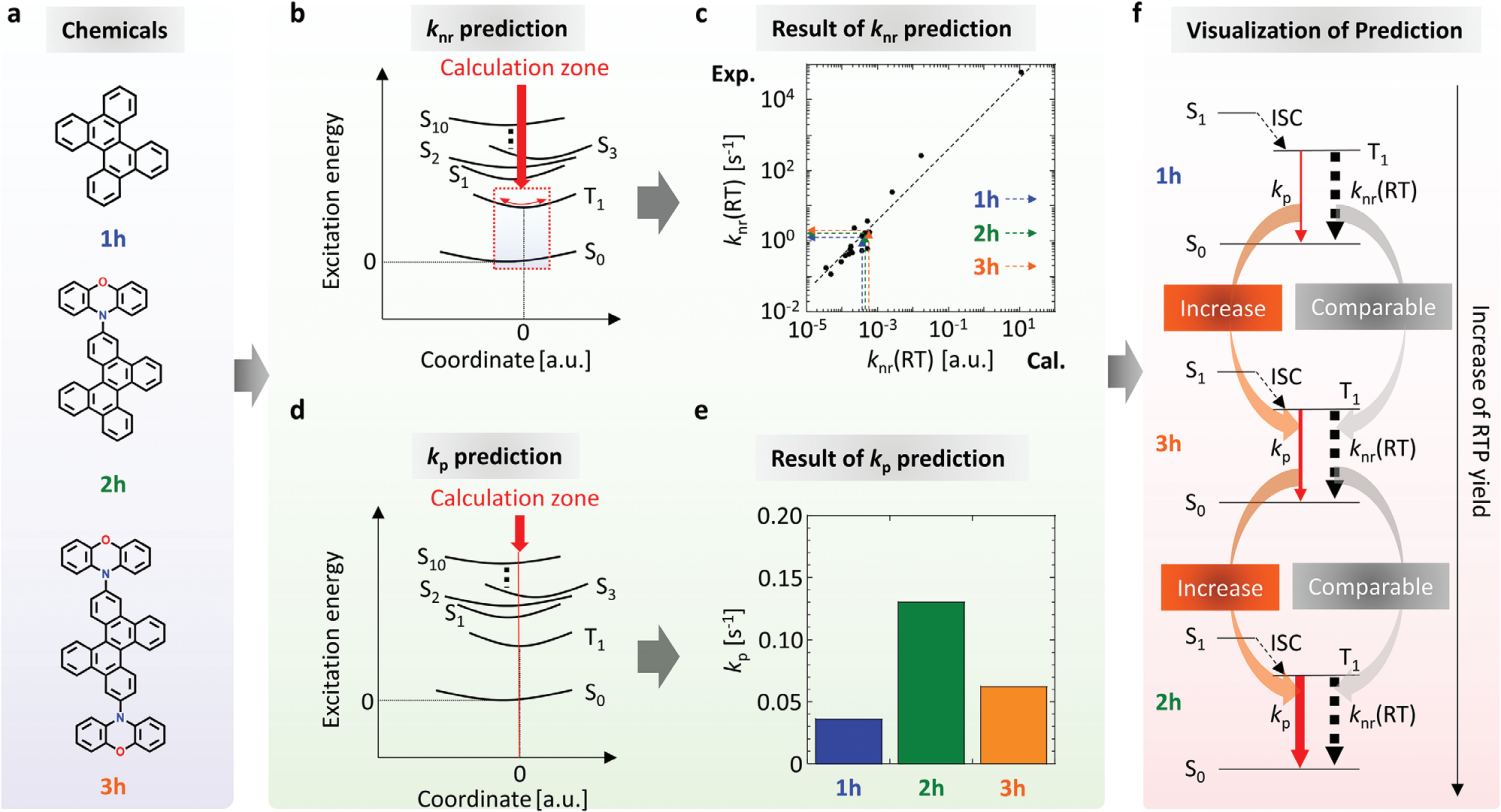
Prediction of red persistent RTP capability of **1h**–**3h** chromophores based on previously reported calculations of *k*_p_ and *k*_nr_(RT). a) Chemical structures. b) Schematic that shows the calculation zone regarding the relationship between the excitation energy and molecular coordinates for predicting *k*_nr_(RT). c) Predicted results of *k*_nr_(RT) using the procedure in (b) for **1h**–**3h**. Black‐filled circles represent previously reported data for other chromophores; reported in references,^[^ ^3^, ^4^, ^6^ ^]^ and.^[^ ^10^ ^]^ The Black dashed line represents a supporting line with a slope of 1. d) Schematic that shows the calculation zone regarding the relationship between the excitation energy and molecular coordinates for predicting *k*_p_. e) Predicted result of *k*_p_ using the procedure in (d) for **1h**–**3h**. f) Visualization of predicted results regarding *k*_p_ and *k*_nr_(RT).

DBC derivatives are molecules with red T_1_ energy.^[^ ^1^, ^4^ ^]^ Three kinds of DBC derivatives [chromophores **1h–3 h** (**Figure** **1**a)] are the focus of our work. For the three chromophores, we calculated the change of *k*_p_ and *k*_nr_(RT) using previously reported quantum chemical calculation procedures to predict the order of the persistent RTP yield. From investigations within the past 10 years, the rate of triplet deactivation at RT can be divided into the rate constant of the intramolecular nonradiative transition at RT [i.e., *k*_nr_(RT)] of the guest chromophores and the triplet quenching rate (*k*_q_), caused by the intermolecular interactions between the guest chromophores and the host at RT.^[^ ^8^ ^]^ Because the T_1_ energy of red RTP chromophores is much smaller than that of chromophores with short conjugated host molecules, *k*_q_(RT) is commonly suppressed when red RTP chromophores with small T_1_ energy are dispersed into short conjugated host molecules with large T_1_ energy and strong oxygen barrier characteristics.^[^ ^1^, ^8^, ^9^ ^]^ Regarding *k*_nr_(RT), predicting *k*_nr_(RT) for a variety of dispersed chemical structures was recently made possible, by calculating spin–orbit coupling (SOC) between T_1_ and S_0_, considering vibrations activated at RT (Figure 1b), because optically measured *k*_nr_(RT) has a good correlation with the calculated value for a variety of chemical structures (Figure 1c).^[^ ^3^, ^4^, ^6^, ^8^, ^10^ ^]^ Analysis using the reported vibrational SOC analysis suggests that *k*_nr_(RT) of **1h–3 h** are comparable and located in the range of 1×10° to 2×10° s^−1^ (Figure 1c). Regarding *k*_p_, the *n*th‐order singlet excited state (S*_n_*) is substantially related to *k*_p_, and *k*_p_ is substantially related to the transition dipole moment between S*_n_* and S_0_ ($\mu_{S_{n}-S_{0}}$) and the SOC between S_n_ and T_1_ ($\left\langle S_{n}|{\hat{H}}_{\mathrm{SO}}|T_{1}\right\rangle$).^[^ ^6^, ^11^ ^]^ However, substantial time is usually necessary to calculate $\mu_{S_{n}-S_{0}}$ and $\left\langle S_{n}|{\hat{H}}_{\mathrm{SO}}|T_{1}\right\rangle$ (*n* = 1, 2, 3…) for a variety of configurations, depending on vibrations allowed at RT. Therefore, *k*_p_ is often analyzed via calculations of $\mu_{S_{n}-S_{0}}$ and $\left\langle S_{n}|{\hat{H}}_{\mathrm{SO}}|T_{1}\right\rangle$ (*n* = 1, 2, 3…) by using a fixed structure optimized at T_1_ (Figure 1d).^[^ ^3^, ^4^, ^6^, ^11^ ^]^ The calculation of *k*_p_ using the optimized T_1_ geometry predicts the relationship **1** **h** < **3** **h** < **2 h** for the value of *k*_p_ (Figure 1e). Therefore, the predictions of *k*_nr_(RT) and *k*_p_ suggest that the red RTP yield of the three chromophores has a relationship **1** **h** < **3** **h** < **2** **h** when the intersystem crossing yield from the lowest singlet excited state (S_1_) to the triplet states (Φ_isc_) is comparable for the three chromophores (Figure 1f).

### 2.2 Optically Determined *k*_nr_(RT) and *k*_p_

**Figure 2:**
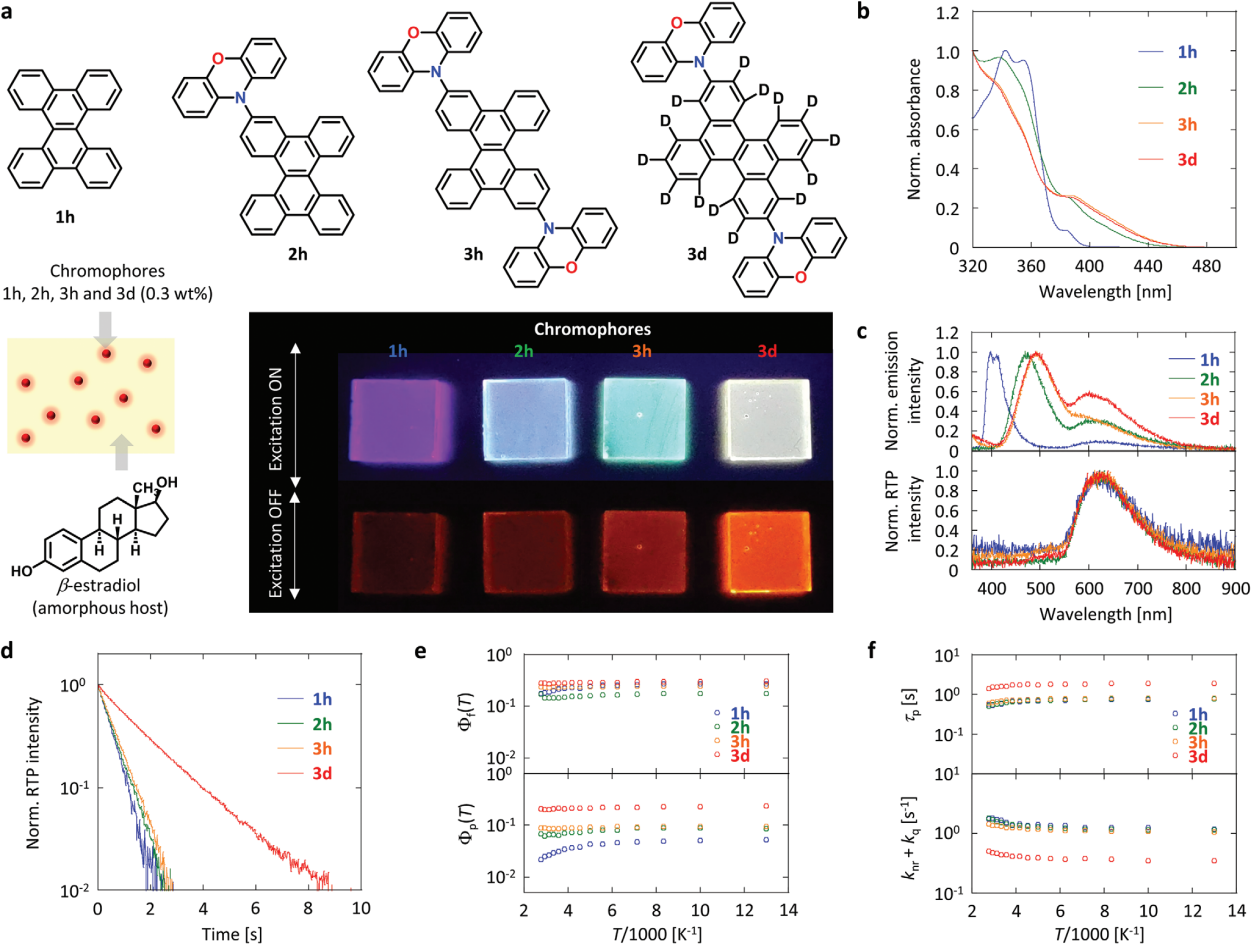
Optical properties of 0.3 wt.% chromophore‐doped amorphous *β*‐estradiol film. a) Chemical structures of **1h**, **2h**, **3h**, and **3d**; and condition of chromophores to generate RTP and photographs of steady‐state RT emission as well as persistent RTP soon after ceasing ultraviolet excitation. b) Absorption spectra. c) Steady‐state RT emission spectra (top) and persistent RTP spectra (bottom). d) RTP emission decay characteristics. e) Temperature dependence of *Φ*_f_ (top) and *Φ*_p_ (bottom). f) Temperature dependence of *τ*_p_ (top) and *k*_nr_ + *k*_q_ (bottom).

Contrary to the prediction in Figure 1, the RTP yield of **3** **h** was larger than that of **2** **h**. We prepared chromophores **1h**–**3 h** and **3d** (**Figure** **2**a) (Section S1 and S2, Supporting Information) and analyzed them by ^1^H nuclear magnetic resonance (NMR), ^13^C NMR, high‐resolution mass spectrometry, and elemental analysis (Section S3 and Figures S1–S17, Supporting Information). Chromophores **1h**–**3 h** and **3d** have absorption wavelengths <460 nm when dispersed in amorphous *β*‐estradiol at a concentration of 0.3 mass fraction (Figure 2b). Chromophores **1h**–**3 h** and **3d** dispersed in amorphous *β*‐estradiol exhibit fluorescence peaks at 397, 469, 494, and 491 nm, respectively (Figure S18, Supporting Information). The additional emission peak at 600–650 nm under excitation at 340 nm is due to the phosphorescence band (Figure 2c, top). Chromophore **3** **h** exhibits stronger emission from 600–650 nm compared with **1** **h** and **2** **h**, and the emission intensity of **3d** from 600–650 nm is increased compared with that of **3** **h**. After ceasing excitation, the red emission with a spectral peak from 600 to 650 nm remained (bottom of Figure 2c). The afterglow emission was caused by persistent RTP because the emission decay had single‐exponential characteristics (Figure 2d). The RTP peak wavelength of **1** **h**, **2** **h**, **3** **h**, and **3d** in amorphous *β*‐estradiol was 624, 626, 625, and 625 nm, respectively; in which radiation with wavelength >600 nm is commonly considered as red emission. The phosphorescence lifetime (*τ*_p_) at RT of 0.3 wt.% **1h**–**3 h** and **3d** dispersed in amorphous *β*‐estradiol was 0.58, 0.61, 0.64, and 1.6 s, respectively. The steady‐state RT emission yield (*Φ*_e_) at RT of 0.3 wt.% **1h**–**3 h** and **3d** dispersed in amorphous *β*‐estradiol was 23%, 21%, 32%, and 49%, respectively. By comparing the spectral intensity of the steady‐state RT emission with that of the persistent RTP soon after ceasing excitation, the phosphorescence yield (*Φ*_p_) at RT of 0.3 wt.% **1h**–**3 h** and **3d** dispersed in amorphous *β*‐estradiol was determined as 2.9%, 6.6%, 8.7%, and 21%, respectively (Figure 2a; Figure S19, Supporting Information). The fluorescence yield (*Φ*_f_) at RT of 0.3 wt.% **1h**–**3 h** and **3d** in dispersed in amorphous *β*‐estradiol was determined as 19%, 14%, 24%, and 28% by substituting *Φ*_p_(RT) from *Φ*_e_(RT). The value of *Φ*_p_(RT) = 21% for **3d** is the largest among previously reported red afterglow RTP‐emitting materials with an RTP peak wavelength >600 nm and an average delayed emission lifetime of >100 ms.^[^ ^12^ ^]^ In delayed fluorescence materials, the delayed emission substantially decreases from 1–100 ms after ceasing excitation, even when afterglow emission is observed (Figure S20, Supporting Information). This causes a large decrease in the average delayed emission lifetime, and the utilization of photons generated after 100 ms substantially decreases. However, the delayed emission from intrinsic RTP emission negligibly decreased from 1 to 100 ms after ceasing excitation. This is suitable for obtaining brighter afterglow harvesting—without interference from autofluorescence—by using a portable charge‐coupled device camera with a slow data collection time (>20 ms) and small‐scale capabilities. The temperature dependence of *Φ*_f_ and *Φ*_p_ indicates that *Φ*_f_ and *Φ*_p_ negligibly decreased from 77 K to RT (Figure 2e; Figure S21, Supporting Information). *τ*_p_(*T*) of the chromophores did not substantially decrease from 77 K to RT (top in Figure 2f; Figure S22, Supporting Information).

### 2.3 Comparison Between Optically Determined Results and Previously Reported Calculation Results for *k*_nr_(RT) and *k*_p_

The optically determined relationship between *k*_nr_(RT) and *k*_p_ had a different tendency compared with the predicted relationship between *k*_nr_(RT) and *k*_p_ in Figure 1. The experimentally determined *k*_nr_(RT) and *k*_p_, *Φ*_isc_ of 0.3 wt.% **1h**–**3 h** and **3d** in amorphous *β*‐estradiol are shown in **Tables** **1** and **2**. The *Φ*_isc_ values calculated using 1 − *Φ*_f_(RT) were ≈80%, 86%, 77%, and 72% for **1h**–**3 h** and **3d**, respectively (Table 1). The approximation of *Φ*_isc_ ≃ 1 − Φ_f_(RT) is reasonable for the chromophores. This is because the value of *Φ*_isc_ in amorphous *β*‐estradiol is comparable to that in benzene (Figures S23–S25 and Table S2, Supporting Information)^[^ ^13^ ^]^ and the fluorescence spectra (Figure 2b; Figure S26b, Supporting Information), as well as the fluorescence lifetime (Figures S18 and S26c, Supporting Information) did not substantially change between benzene and *β*‐estradiol. By substituting *Φ*_p_(RT), *τ*_p_(RT), and *Φ*_isc_ into the common formula *Φ*_p_(RT) = *Φ*_isc_ *k*_p_ *τ*_p_(RT), *k*_p_ of **1h–3 h**, and **3d** dispersed in amorphous *β*‐estradiol was determined as 0.063, 0.13, 0.18, and 0.18 s^−1^, respectively (Table 2). We obtained the temperature dependence of *k*_nr_(*T*) + *k*_q_(*T*) by applying *Φ*_p_(*T*), *k*_p_, and *Φ*_isc_ into *Φ*_p_(*T*) = *Φ*_isc_ *k*_p_/[*k*_p_ + *k*_nr_(*T*) + *k*_q_(*T*)] (Figure 2F, bottom). Assuming that *k*_nr_(*T*) and *k*_q_(*T*) have an exponential function as 1/*T* (Figure S27, Supporting Information), we separated *k*_nr_(*T*) and *k*_q_(*T*) to determine *k*_nr_(RT) and *k*_q_(RT) (Table 2).^[^ ^10^ ^]^ The temperature dependence of the emission properties of thin films doped with 0.3 wt.% **3d** in other representative nonconjugated polymer hosts was also verified. However, *Φ*_p_(RT) in these polymers is smaller than that in *β*‐estradiol (Section S7, Figure S28, Tables S4 and S5, Supporting Information). In these polymers, unlike amorphous *β*‐estradiol, more pronounced multiexponential phosphorescence decay characteristics, which indicate poor dispersion of latent dyes, were also confirmed (Section S7, Figures S29–S31, Supporting Information). Such differences in the degree of aggregation impart difficulties in quantitatively comparing *k*_nr_ and *k*_p_ for each guest chromophore. Therefore, in this paper, data in amorphous *β*‐estradiol were used to discuss the relationship between *k*_nr_, *k*_p_, and the molecular structure of the guest chromophore.

**Table 1:** Summary of photophysical parameters related to singlet states of chromophores (0.3 wt.%) doped into amorphous *β*‐estradiol.

Chromophores	*λ*_f_ ^a)^ [nm]	Φ_f_(RT)	*τ*_f_(RT) [ns]	*k*_f_ ^b)^ [10^7^ s^−1^]	Φ_isc_	*k*_isc_ ^e)^ [10^7^ s^−1^]
**1h**	397	0.20	11	1.8	0.80^c)^ (0.87 ± 0.22)^d)^	7.3
**2h**	469	0.14	3.7	3.9	0.86^c)^ (0.99 ± 0.25)^d)^	23
**3h**	494	0.23	7.2	3.3	0.77^c)^ (0.85 ± 0.21)^d)^	11
**3d**	491	0.28	6.8	4.0	0.72^c)^ (0.88 ± 0.22)^d)^	11

^a)^ Peak wavelength of fluorescence;

^b)^ Value determined with *k*_f_ = Φ_f_(RT)/*τ*_f_(RT);

^c)^ Value determined based on Φ_isc_ = 1 − Φ_f_(RT);

^d)^ Value determined by using transient absorption procedure in benzene;

^e)^ Value determined with *k*_isc_ = Φ_isc_/*τ*_f_(RT).

**Table 2:** Summary of photophysical parameters related to triplet states.

Chromo‐phores	Experimental^a)^						Calculation		
	Φ_p_(RT)	*τ*_p_(RT) ^c)^ (s)	*λ*_p_ ^d)^ (nm)	*k*_p_ ^e)^ (s^‐1^)	*k*_nr_(RT) ^f)^ (s^‐1^)	*k*_q_(RT) ^g)^ (s^‐1^)	$k_{p\;T_{1}\mathrm{opt}}$ ^h)^ (s^‐1^)	*k*_p θ_ (s^‐1^)	$\sum_{p}{|\partial \left\langle T_{1}\left|{\hat{H}}_{\mathrm{SO}}\right|S_{0}\right\rangle \partial Q_{p}|}^{2}P\left(\mathrm{RT}\right)FC$ ^k)^ (a.u.)
**1h** ^b)^	0.029	0.58	624	0.063	1.4	0.25	0.036	0.036^h)^	3.8
**2h** ^b)^	0.066	0.61	626	0.13	1.2	0.30	0.13	0.073^i)^	4.4
**3h**	0.087	0.64	625	0.18	1.1	0.42	0.062	0.18^j)^	5.7
**3d**	0.21	1.6	625	0.18	0.39	0.061	—	—	—

^a)^ Values by using 0.3 wt % chromophore doped in *β*‐estradiol;

^b)^ Experimental data have been reported in reference ^[^ ^4h^ ^]^;

^c)^ Single‐exponential fitting data;

^d)^ Peak wavelength of phosphorescence;

^e)^ Values determined by substituting experimentally observed Φ_p_(RT), *τ*_p_(RT), and Φ_isc_ = 1 − Φ_f_ (RT) into *k*_p_ = Φ_p_(RT)/(Φ_isc_ *τ*_p_);

^f)^ Values determined by using fitting lines of *k*_nr_(T) in Figure S27 (Supporting Information);

^g)^ Values determined by subtracting *k*_nr_(RT), determined by using the fitting lines of *k*_nr_(T) in Figure S27 (Supporting Information), from experimentally observed *k*_nr_(T) + *k*_q_(T);

^h)^ Calculated values by using the PBE0 functional and TZP basis sets, by using the optimized structure at T_1_ by DFT (Gaussian09/B3LYP/6‐31G(d));

^i)^ Figure S36 (Supporting Information) shows the information for calculations;

^j)^ Figure 4 shows the information for calculations;

^k)^ This factor is proportional to *k*_nr_(RT). Figures S32–S34 and Tables S6 and S7 (Supporting Information) show the information for calculations.

The optically measured *k*_nr_(RT) of **1h**–**3 h** in amorphous *β*‐estradiol was comparable and in the range of 1×10° to 2×10° s^−1^. The decrease of the optically observed *k*_nr_(RT) of **3d** compared with **3** **h** can be explained by the common isotope effect.^[^ ^1^, ^6^ ^]^ The determined values of *k*_nr_(RT) of **1h**–**3 h** closely corresponded to a previously determined correlation line between the optically determined *k*_nr_(RT) and calculated *k*_nr_(RT) values, considering all vibrations allowed at RT (Figures S32–S34, Tables S6 and S7, Supporting Information). Therefore, the prediction of *k*_nr_(RT) in Figure 1c worked well for **1h**–**3 h**.

**Figure 3:**
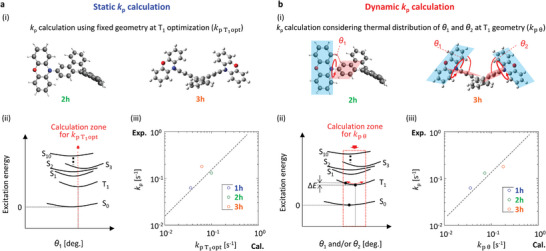
Comparison of results regarding the prediction of *k*_p_ between two procedures. a) Static *k*_p_ calculation: Case by using calculation procedure based on a fixed T_1_ geometry. b) Dynamic *k*_p_ calculation: Case by using calculation procedure considering the change *θ*_1_ and/or *θ*_2_, depending on the thermal energy. (i) Molecular condition of **2h** and **3h** for calculations. (ii) Schematics showing a zone that we used to calculate *k*_p_ in a Jablonski diagram. (iii) Result of the relationship between the optically measured result and the calculated result. In (iii), the dashed line represents a supporting line with a slope of 1.

However, the previously reported static *k*_p_ prediction by using optimized T_1_ geometries did not work for **1h–3 h**. When we considered the experimental result and the predicted result regarding *k*_p_, calculated based on the fixed T_1_ geometry ($k_{p\;T_{1}\mathrm{opt}}$) (**Figure** **3**a‐(i),(ii)) for **1h**–**3 h**, the relationship between the optically observed *k*_p_ and the calculated $k_{p\;T_{1}\mathrm{opt}}$ of **3** **h** did not have a strong correlation (Figure 3a‐(iii)). In addition, analysis after adding a D‐*π*‐D structure with a different chemical backbone from DBC exhibited a deteriorated correlation for the *k*_p_ versus $k_{p\;T_{1}\mathrm{opt}}$ plot (Figure S35a, Supporting Information). This indicates that previously reported static *k*_p_ calculations based on a fixed T_1_ geometry cannot be universally used to predict *k*_p_ of conjugated structures. Upon focusing the data of **1** **h** and **2** **h** in *k*_p_ versus $k_{p\;T_{1}\mathrm{opt}}$ plots (Figure 3a‐(iii)), the data of **1** **h** and **2** **h** are close in a manner that results in a supporting correlation line with a slope of 1; whereas the data of **3** **h** were somewhat far from the line. Therefore, we potentially underestimated the calculated $k_{p\;T_{1}\mathrm{opt}}$ of **3** **h**.

### 2.4 Role of Symmetry‐Breaking in Selectively Enhancing *k*_p_ Compared with *k*_nr_(RT)

**Figure 4:**
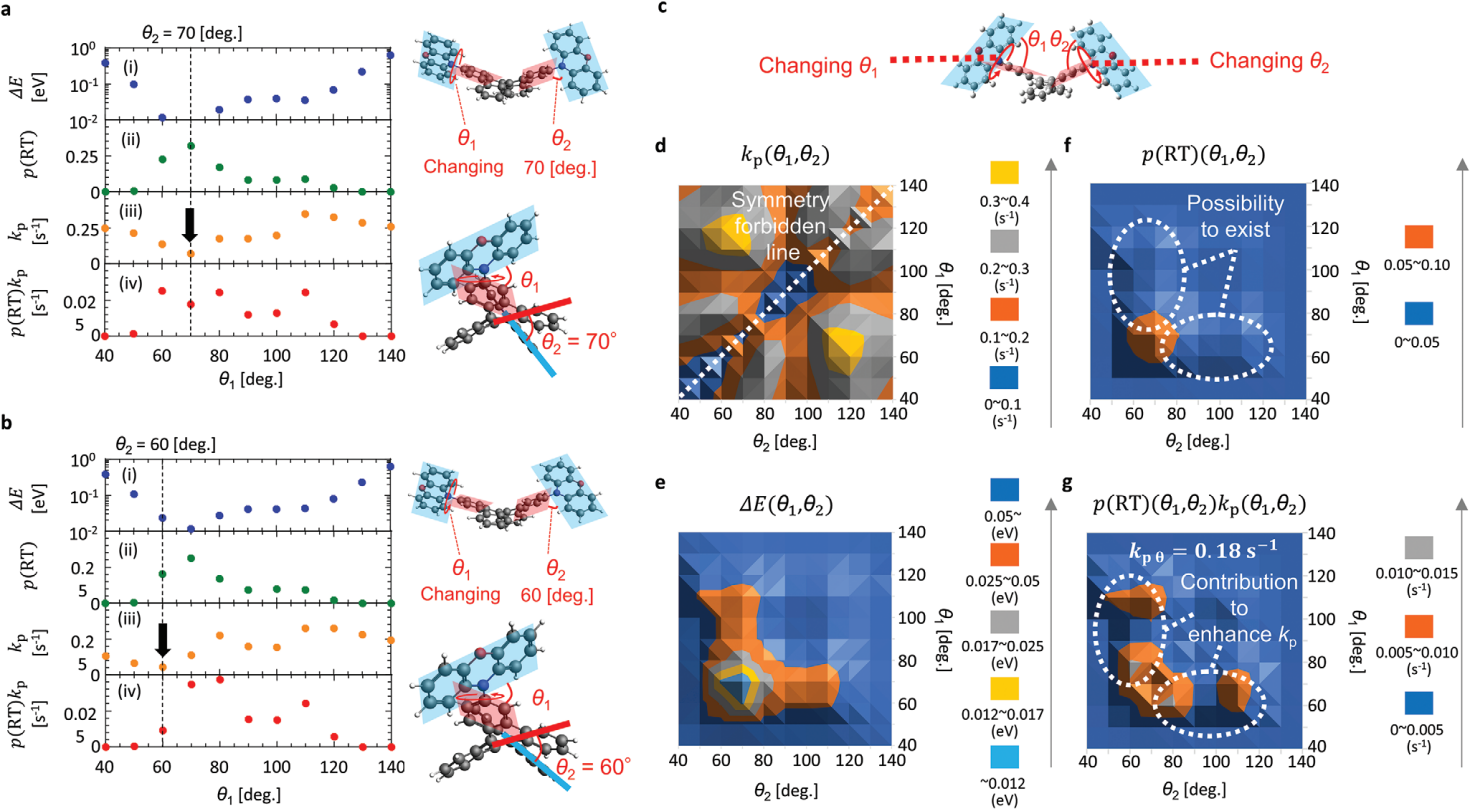
Contribution of the change of *θ*_1_ and *θ*_2_, considering the thermal energy to *k*_p_ of **3h**. a) Change of Δ*E* (i), *p*(RT) (ii), *k*_p_ (iii), and *p*(RT)*k*_p_ (iv) upon changing *θ*_1_ yet keeping *θ*_2_ = 70°. b) Change of Δ*E* (i), *p*(RT) (ii), *k*_p_ (iii), and *p*(RT)*k*_p_ (iv) upon changing *θ*_1_ yet keeping *θ*_2_ = 60°. c) Molecular illustration that explains the independent change of *θ*_1_ and *θ*_2_ for **3h**. d–g) 2D histograms of *k*_p_, Δ*E* (e), *p*(RT), and *p*(RT)*k*_p_ upon independently changing *θ*_1_ and *θ*_2_.

The reason for the potential underestimation of the *k*_p_ of **3** **h** is related to the symmetric structure of **3** **h**. Here, the thermal distribution depending on the dihedral angle between phenoxazine and the DBC unit (*θ*_1_ for **2** **h**, and *θ*_1_ and *θ*_2_ for **3** **h**) is considered (Figure 3b‐(i). We performed a dynamic calculation of *k*_p_ by considering the independent change of *θ*_1_ and *θ*_2_, based on the thermal energy (*k*_p θ_) (Figure 3b‐(ii)). The result exhibited a much better correlation between the optically measured *k*_p_ and the predicted result (*k*_p θ_), based on the proposed dynamic calculation (Figure 3b‐(iii)). In addition, analysis after adding a D‐*π*‐D structure with a different chemical backbone from DBC exhibited a good correlation for the *k*_p_ versus *k*_p θ_ plot (Figure S35b, Supporting Information). To perform the calculation shown in Figure 3b for **3** **h**, we initially determined the T_1_‐optimized geometry. *θ*_1_ and *θ*_2_ of the optimized T_1_ geometry were ≈70°. Next, we calculated the energy increase compared with the optimized T_1_ geometry (Δ*E*) (Figure 3b (ii)) upon changing *θ*_1_ yet fixing *θ*_2_ = 70° (**Figure** **4**a‐(i)). By using the Δ*E*, we calculated the possibility of each distorted geometry at RT [*p*(RT)], depending on the change of *θ*_1_, by using $p\left(\mathrm{RT}\right)\;\left(\theta_{1}\right)=\exp\left(-\Delta E(\theta_{1})/kT\right)/\int \exp\left(-\Delta E(\theta_{1})/kT\right)d\theta_{1}\;$ based on the Boltzmann distribution (Figure 4a (ii)), where *k* is the Boltzmann constant. Upon changing *θ*_1_ from 70°, *p*(RT) decreased. For the various geometries, we performed the calculation of *k*_p_ (Figure 4a‐(iii)). As a crucial point, *k*_p_ had the smallest value when *θ*_1_ = *θ*_2_ = 70° (arrow in Figure 4a‐(iii)). Then, we calculated Δ*E*, *p*(RT), and *k*_p_ in the same manner as upon changing *θ*_1_ yet fixing *θ*_2_ = 60° (Figure 4b). Similarly, we observed the smallest value of *k*_p_ when *θ*_1_ = *θ*_2_ = 60° (arrow in Figure 4b‐(iii)). Thus, the origin of small *k*_p_ for symmetric D‐*π*‐D structures in which *θ*_1_ = *θ*_2_ needs to be understood.

To elaborate on this point, we constructed a 2D histogram of *k*_p_ regarding independent changes of *θ*_1_ and *θ*_2_ (Figure 4c) [*k*_p_(*θ*_1_, *θ*_2_)] (Figure 4d). Because there was a large decrease in *k*_p_ values for *θ*_1_ = *θ*_2_, a symmetry‐forbidden line regarding *k*_p_ was evident. This indicates a mechanism for the observation that symmetric structures of **3** **h** corresponded to substantially decreased *k*_p_. We also calculated the dependence of Δ*E* on independent changes of *θ*_1_ and *θ*_2_ [Δ*E*(*θ*_1_, *θ*_2_)] (Figure 4e). By using Δ*E*(*θ*_1_, *θ*_2_), we calculated *p*(RT) (depending on independent changes of *θ*_1_ and *θ*_2_ [*p*(RT)(*θ*_1_, *θ*_2_)]) by using $p\left(\mathrm{RT}\right)\;\left(\theta_{1},\theta_{2}\right)=\exp\left(-\Delta E\left(\theta_{1},\theta_{2}\right)/\mathrm{kT}\right)/\;\int \int \;\exp\left(-\Delta E\left(\theta_{1},\theta_{2}\right)/\mathrm{kT}\right)d\theta_{1}d\theta_{2}\;$ based on the Boltzmann distribution (Figure 4f). Finally, we calculated a 2D histogram of *p*(RT)(*θ*_1_, *θ*_2_)*k*_p_(*θ*_1_, *θ*_2_) from data in Figure 4e–g). Integration of *p*(RT)(*θ*_1_, *θ*_2_)*k*_p_(*θ*_1_, *θ*_2_) regarding *θ*_1_ and *θ*_2_ was by the average value of *k*_p_ (*k*_p θ_) when *θ*_1_ and *θ*_2_ were distributed based on the Boltzmann theory. We calculated the *k*_p θ_ value of **3** **h** to be 0.18 s^−1^. Regarding **2** **h**, we considered the change of *θ*_1_ and determined *k*_p θ_ to be 0.073 s^−1^ (Figure S36, Supporting Information). As a result, we observed a good correlation between the optically determined *k*_p_ and the calculated *k*_p θ_ based on dynamic calculation for **1h**–**3 h** (Figure 3b‐(iii)). Therefore, **2** **h** and **3** **h** have a distribution of *θ*_1_ and *θ*_2_ even upon dispersal into a solid host. The static calculations that do not consider the coordinate change based on the thermal distribution resulted in three times smaller $k_{p\;T_{1}\mathrm{opt}}$ (0.062 s^−1^) based on fixed T_1_ geometry compared with *k*_p θ_ (0.18 s^−1^) of **3** **h** chromophore. However, the changes in the dihedral angles (*θ*_1_ and *θ*_2_) were negligibly related to *k*_nr_(RT) (Section S8 and Figure S37, Supporting Information).

### 2.5 Origin of Enhancing *k*_p_ by Symmetry‐Breaking

**Figure 5:**
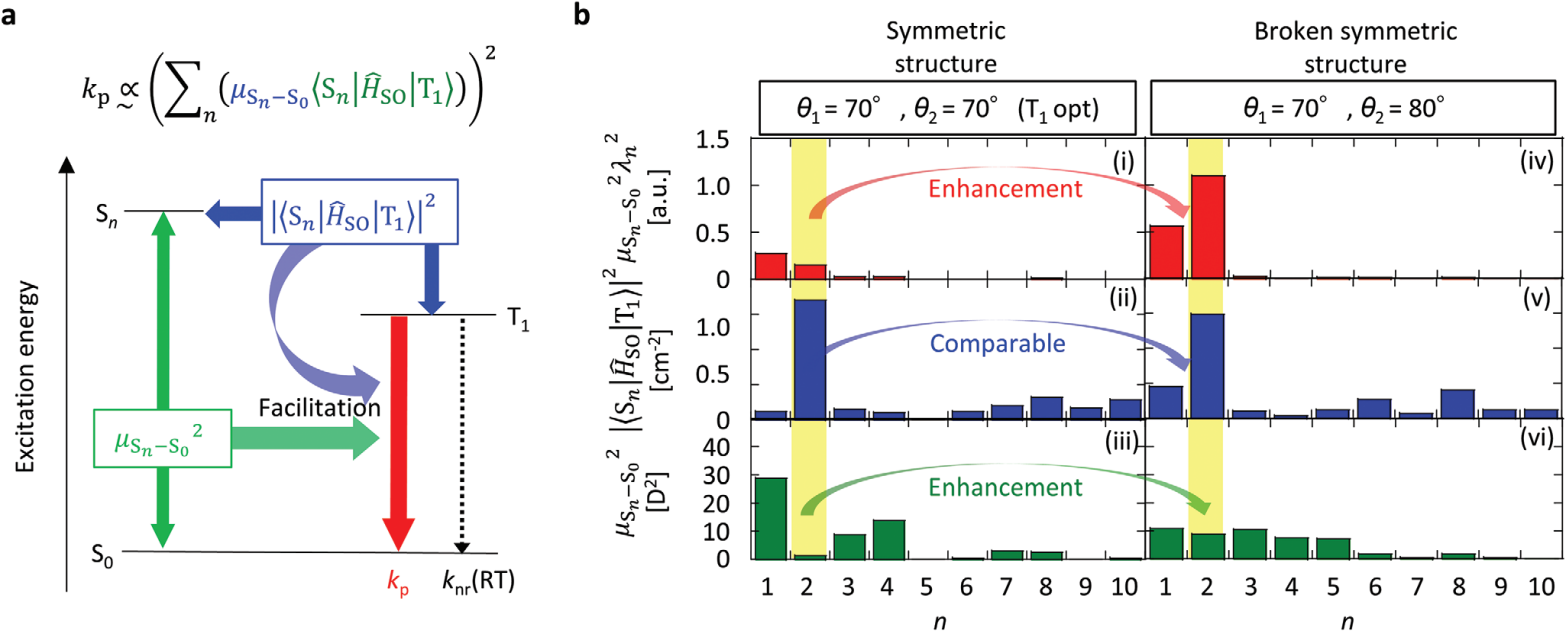
Identification of the primary contributing factor for enhanced *k*_p_ in **3h** in terms of the transition dipole moment as well as SOC. a) Energy diagram to explain the relationship among *k*_p_, ${\mu_{S_{n}-S_{0}}}^{2}$, and ${\left\langle S_{n}|{\hat{H}}_{\mathrm{SO}}|T_{1}\right\rangle }^{2}$. b) Relationship among *n*, ${\mu_{S_{n}-S_{0}}}^{2}{\lambda_{n}}^{2}$ (i), ${\left\langle S_{n}|{\hat{H}}_{\mathrm{SO}}|T_{1}\right\rangle }^{2}$ (ii), and ${\mu_{S_{n}-S_{0}}}^{2}$ (iii) of a symmetric geometry in which *θ*_1_ = 70° and *θ*_2_ = 70° (left) and a disrupted symmetric geometry in which *θ*_1_ = 70° and *θ*_2_ = 80° (right).

Although the contribution of symmetry‐breaking by changing *θ*_1_ and *θ*_2_ to enhanced *k*_p_ is clear, further detailed analysis regarding parameters contained in *k*_p_ indicates that an increase of the S*_n_*–S_0_ transition dipole moment by changing *θ*_1_ and *θ*_2_ enhanced *k*_p_. For some chromophores in previous reports, *k*_p_ is approximately calculated based on the following formula,^[^ ^6^, ^11^ ^]^

(1) $$k_{p}\propto E_{T_{1}-S_{0}}^{3}{\left[\sum_{n}\left(\mu_{S_{n}-S_{0}}\lambda_{n}\right)\right]}^{2}$$

(2) $$\lambda_{n}≃\langle S_{n}|{\hat{H}}_{\mathrm{SO}}|T_{1}\rangle /E_{S_{n}-T_{1}}$$

where $E_{T_{1}-S_{0}}$ is the energy difference between T_1_ and S_0_, $\mu_{S_{n}-S_{0}}$ is the transition dipole moment between S*_n_* and S_0_, $\left\langle S_{n}|{\hat{H}}_{\mathrm{SO}}|T_{1}\right\rangle$ is the SOC between S*_n_* and T_1_, and $E_{S_{n}-T_{1}}$ is the energy difference between S*_n_* and T_1_. The calculated values of $E_{T_{1}-S_{0}}$ in **1h–3 h** are comparable (Figure S38, Supporting Information) and reasonably explain the red energy of the chromophores, upon considering a statistical relationship between the optically determined $E_{T_{1}-S_{0}}$ values and the calculated $E_{T_{1}-S_{0}}$ (Figure S39, Supporting Information). Because of the comparable $E_{T_{1}-S_{0}}$ among **1h**–**3 h**, the difference of *k*_p_ could correspond to a different $\mu_{S_{n}-S_{0}}\lambda_{n}$ based on Equation (1). On the basis of Equations (1) and (2), *k*_p_ is approximately proportional to $\mu_{S_{n}-S_{0}}$ ^2^ and $\left\langle S_{n}|{\hat{H}}_{\mathrm{SO}}|T_{1}\right\rangle$ ^2^ (**Figure** **5**a). Therefore, visualization of $\mu_{S_{n}-S_{0}}$ ^2^ and $\left\langle S_{n}|{\hat{H}}_{\mathrm{SO}}|T_{1}\right\rangle$ ^2^ for each *n* (Figure 5b) is useful for understanding the main contribution to the selective enhancement of *k*_p_. For the symmetric optimized T_1_ geometry of **3** **h** (*θ*_1_ = 70°, *θ*_2_ = 70°), $\mu_{S_{n}-S_{0}}$ ^2^λ_*n*_^2^ in *n* = 1 and *n* = 2 mainly contributed to *k*_p_; whereas $\mu_{S_{n}-S_{0}}$ ^2^λ_*n*_^2^ in *n* = 2 exhibited a lesser contribution (Figure 5b‐(i)). In S_2_, the small $\mu_{S_{2}-S_{0}}$ ^2^ (Figure 5b‐(iii)) corresponded to the small $\mu_{S_{2}-S_{0}}$ ^2^λ_2_^2^, although $\left\langle S_{2}|{\hat{H}}_{\mathrm{SO}}|T_{1}\right\rangle$ ^2^ was large (Figure 5b‐(ii)). Therefore, the small $\mu_{S_{2}-S_{0}}$ ^2^ is a reason for the underestimated $k_{p\;T_{1}\mathrm{opt}}$ (0.063 s^−1^) for the symmetric optimized T_1_ structure of **3** **h**. However, there was also a disrupted symmetric geometry with *θ*_1_ = 70° and *θ*_2_ = 80°, depending on the thermal distribution at RT (Figure 4f). Regarding the thermally activated broken symmetric geometry in which *θ*_1_ = 70° and *θ*_2_ = 80°, ${\mu_{S_{2}-S_{0}}}^{2}$ substantially increased (Figure 5b‐(vi)); whereas $\left\langle S_{2}|{\hat{H}}_{\mathrm{SO}}|T_{1}\right\rangle$ ^2^ did not substantially decrease (Figure 5b‐(v)). Because $\mu_{S_{2}-S_{0}}$ ^2^λ_2_^2^ of the disrupted symmetric geometry substantially increased compared with the symmetric geometry in which *θ*_1_ = *θ*_2_ = 70° (Figure 5b‐(iv)), the increase of ${\mu_{S_{2}-S_{0}}}^{2}$ contributed to the enhanced *k*_p_.

**Figure 6:**
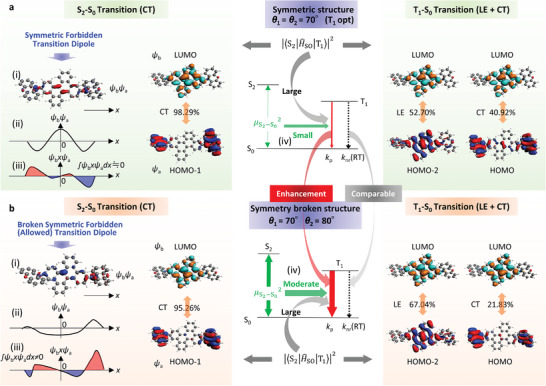
Visualization of breaking symmetry‐forbidden transition contributing to selective *k*_p_ enhancement in **3h**. a,b) Schematic of molecular orbitals for *k*_p_ enhancement attributable to the S_2_–S_0_ transitions for a symmetric geometry in which *θ*_1_ = *θ*_2_ = 70° and a disrupted symmetric geometry in which *θ*_1_ = 70° and *θ*_2_ = 80°.

The large enhancement of ${\mu_{S_{2}-S_{0}}}^{2}$ by symmetry‐breaking can be understood by considering the symmetry‐forbidden transition dipole moment in the S_2_–S_0_ transition. We defined two molecular orbitals primarily relating to the S_2_–S_0_ transition as *ψ*_a_ and *ψ*_b_. Regarding the symmetric geometry with *θ*_1_ = *θ*_2_ = 70°, the overlapping density between *ψ*_a_ and *ψ*_b_ (*ψ*_a_ *ψ*_b_) becomes an even function along the *x*‐axis (**Figure** **6**a‐(i)). Therefore, *ψ*_a_x*ψ*_b_ becomes an odd function along the *x*‐axis (Figure 6a‐(ii)); hence ∫*ψ*_a_x*ψ*_b_dx approaches 0 (Figure 6a‐(iii)). Therefore, $\mu_{S_{2}-S_{0}}$∝ |∫*ψ*_a_x*ψ*_b_dx|^2^ becomes small and negligibly contributes to the enhancement of *k*_p_ (Figure 6a‐(iv)). However, the even functional characteristics along the *x*‐axis of *ψ*_a_ *ψ*_b_ are slightly disrupted for the thermally activated asymmetric geometry in which *θ*_1_ = 70° and *θ*_2_ = 80° (Figure 6b‐(i),(ii)). Therefore, a much larger value remains for ∫*ψ*_a_x*ψ*_b_dx (Figure 6b‐(iii)) for the symmetry‐breaking geometry compared with the optimized symmetric geometry in which *θ*_1_ = *θ*_2_ = 70° at T_1_. The large enhancement of ${\mu_{S_{2}-S_{0}}}^{2}$ is because of the enhanced ∫*ψ*_a_x*ψ*_b_dx that facilitates *k*_p_ (Figure 6b‐(iv)). When *θ*_2_ approaches 90° upon fixing *θ*_1_ = 70°, a phenoxazine unit becomes perpendicular to the DBC unit. Therefore, ${\mu_{S_{2}-S_{0}}}^{2}$ may receive a negative contribution to decrease the value when electron transfer between phenoxazine unit and DBC unit is related to ${\mu_{S_{2}-S_{0}}}^{2}$. However, the contribution of breaking the symmetry‐forbidden transition to enhance ${\mu_{S_{2}-S_{0}}}^{2}$ is still substantial. Therefore, *k*_p_ of the geometry in which *θ*_1_ = 70° and *θ*_2_ = 90° is still large compared with that of the T_1_‐optimized geometry in which *θ*_1_ = *θ*_2_ = 70° (Figure 4a‐(iii)). We report the same mechanism of the selective *k*_p_ enhancement for a different symmetry‐disrupted geometry in which *θ*_1_ = 70° and *θ*_2_ = 50°. We observed an enhancement of ${\mu_{S_{2}-S_{0}}}^{2}$ and a negligible decrease of $\left\langle S_{2}|{\hat{H}}_{\mathrm{SO}}|T_{1}\right\rangle$ ^2^ for the geometry (Figure S40a, Supporting Information). The enhancement of ${\mu_{S_{2}-S_{0}}}^{2}$ could be explained by breaking the symmetry‐forbidden transition nature as well (Figure S40b, Supporting Information).

### 2.6 Universality of Symmetry‐Breaking Phosphorescence Enhancement in a Variety of D‐*π*‐D Structures

**Figure 7:**
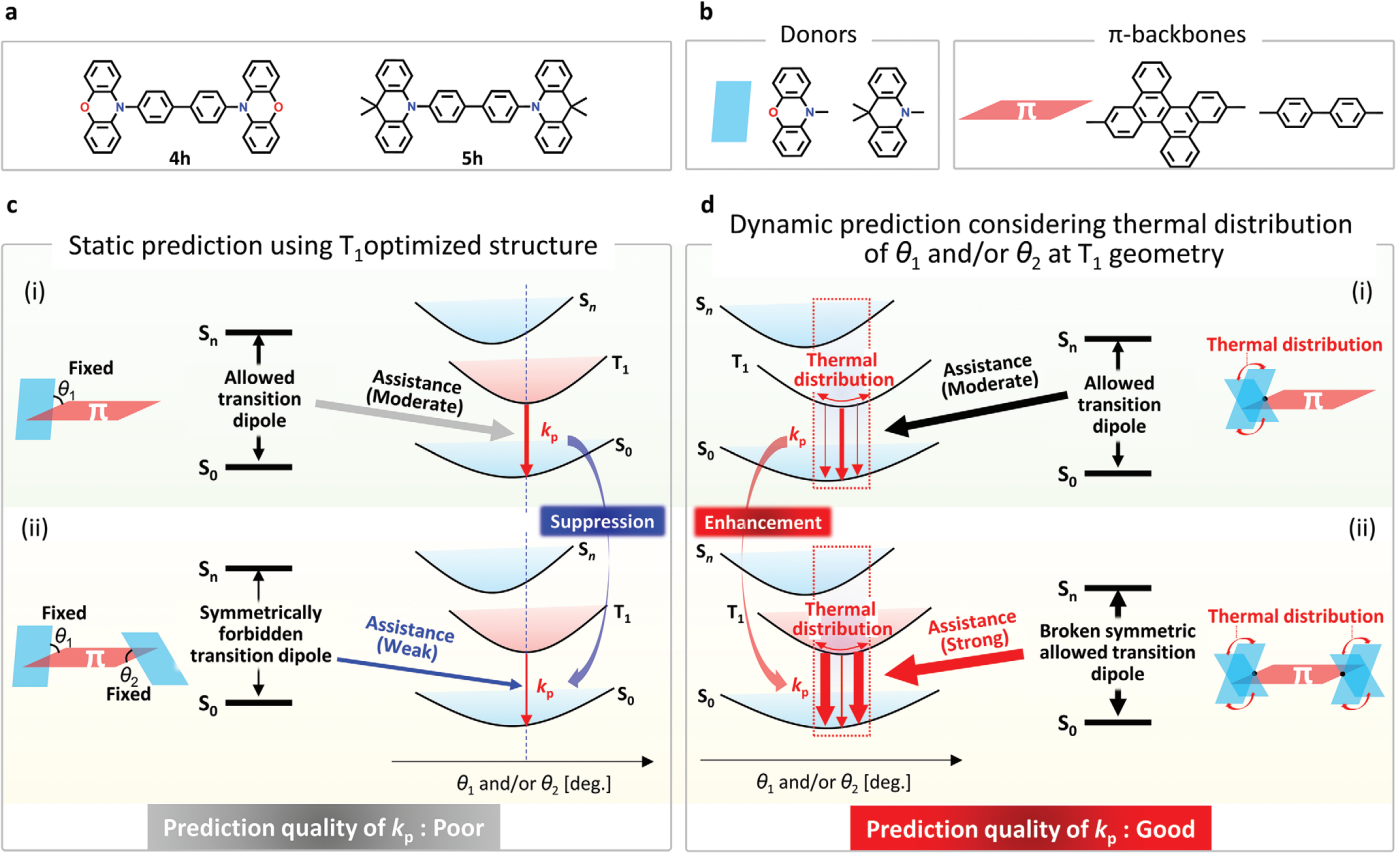
Role of thermally activated symmetry‐breaking that facilitates a triplet radiation rate in a variety of D‐*π*‐D structures. a) Other examples of D‐*π*‐D structures that exhibit the enhancement of triplet radiation by symmetry‐breaking. b) Examples of donors and *π* backbones that enable enhancement of triplet radiation by symmetry‐breaking. c) Difference between the calculated *k*_p_ in the context of D‐*π* and D‐*π*‐D structures upon static condition using the optimized T_1_ geometry. d) Difference between the calculated *k*_p_ in the context of D‐*π* and D‐*π*‐D structures upon dynamic condition considering the thermal distribution of the dihedral angle between the donor and *π* backbone.

The selective increase of *k*_p_ caused by the symmetry‐broken molecular structure is not exclusive to the **3** **h** chromophore but also applies to other D‐*π*‐D structures. For example, **4** **h** (**Figure** **7**a, in which a DBC core is changed into a biphenyl core from **3** **h**) exhibited a small value of *k*_p_ (7.0×10^−3^ s^−1^) when calculated based on a fixed optimized geometry at T_1_. In the T_1_ geometry, **4** **h** has a symmetric structure, and *θ*_1_ as well as *θ*_2_ of **4** **h** are ≈60°. However, the optically observed *k*_p_ was 0.61 s^−1^ (Figure S41, Supporting Information). This is ≈100× larger than that calculated by using the fixed T_1_ geometry. However, upon considering an independent distribution of *θ*_1_ and *θ*_2_ based on the Boltzmann distribution, the calculated *k*_p θ_ was 0.95 s^−1^ (Figure S42, Supporting Information). This is close to the optically observed *k*_p_. Because the calculated *k*_p θ_ of the D‐*π*‐D structure **4** **h** (0.95 s^−1^) is larger than that of D‐*π* structure (0.30 s^−1^) (Figure S43, Supporting Information), D‐*π*‐D structures are more suitable for enhancing *k*_p_. In the fixed geometry optimized at T_1_ of **4** **h** (*θ*_1_ = *θ*_2_), the S_2_–S_0_ transition becomes symmetry‐forbidden and does not contribute to the enhancement of *k*_p_ (Figures S44a and S44c, Supporting Information). However, for a thermally activated asymmetric geometry at RT of **4** **h** (*θ*_1_ ≠ *θ*_2_), the disrupted symmetry‐forbidden S_2_–S_0_ transition can enhance *k*_p_ (Figures S44b and S44d, Supporting Information). Regarding **5 h** in which the two phenoxazine donating units of **4** **h** change into two acridine donating units (Figure 7a), we also calculated the symmetric structure along the *x*‐axis at the T_1_ geometry. The *k*_p_ was calculated by using the optimized symmetric T_1_ geometry in which *θ*_1_ = *θ*_2_ was small (Figure S45a, Supporting Information) whereas the calculated *k*_p_ increased upon disrupting the symmetric geometry (*θ*_1_≠*θ*_2_) (Figure S45b, Supporting Information). Hence, the symmetry‐breaking induced selective enhancement of the *k*_p_ phenomenon proved to be universal for various D‐*π*‐D structures composed of planer D substituents and a *π* unit (Figure 7b). Regarding D‐*π* structures, the symmetry‐forbidden transition was not evident. Therefore, there might not be a substantial underestimation of *k*_p_ for D‐*π* structures even upon using the optimized T_1_ geometry for predicting *k*_p_ (Figure 7c‐(i)). However, because there are symmetry‐forbidden transition characteristics for the S*_n_*–S_0_ transition of D‐*π*‐D structures, static calculations that use the symmetry‐optimized T_1_ structure cause the substantial underestimation of *k*_p_ (Figure 7c‐(ii)), also resulting in under‐prediction of *k*_p_ compared with the corresponding result for the D‐*π* structure. The dynamic calculation that considered the statistic distribution of *θ*_1_ and *θ*_2_, based on thermal energy, appropriately considered the disrupted symmetry‐forbidden transition dipoles between the S*_n_* and S_0_ transition (Figure 7d‐(ii)). This leads to an appropriate prediction that *k*_p_ of the D‐*π*‐D structures is larger than the *k*_p_ of the D‐*π* structures.

### 2.7 Ubiquitous Afterglow Readout

**Figure 8:**
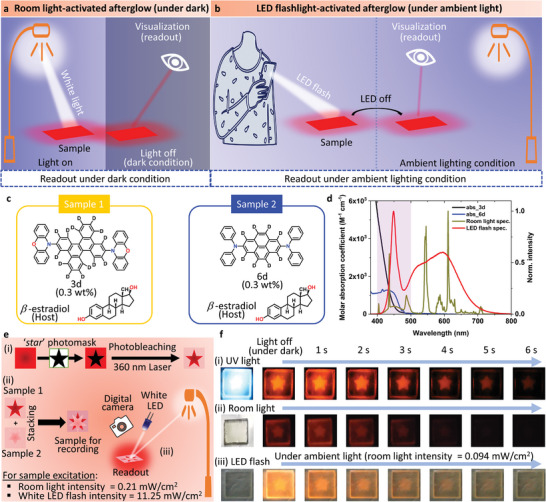
Demonstration of ubiquitous afterglow readout. a) White room light‐activated afterglow readout with unaided eyes in the dark as a ubiquitous afterglow readout. b) White LED‐activated bright afterglow readout with unaided eyes in white room light. c) Structure and mass ratio of **3d**, **6d**, and *β*‐estradiol used in sample 1 (left) and sample 2 (right). d) Comparison of absorption spectra of **3d** and **6d**, and light emission spectra of white room light and white LED. The overlap between the excitation and absorption spectra in the 420–480‐nm (sky blue color) range indicates that visible light is excitable. e) Schematic explaining the preparation procedure and afterglow readout procedure for samples with a stacked structure of samples 1 and 2. f)‐(i) Demonstration of conventional afterglow readout from the stacked sample in the dark by using ultraviolet excitation. (ii) Demonstration of white room light‐activated afterglow readout with unaided eyes in the dark of the stacked sample. The intensity of white room light is 0.21 mW cm^−2^ at the position of the sample upon conversion of the number of photons in the white light at 532 nm. (iii) White LED light‐activated bright afterglow readout with unaided eyes under the white room light of the stacked sample. The intensity of white light excitation from the LED is 11.25 mW cm^−2^ at the sample position of the number of photons in the white light at 532 nm. The Intensity of white room light is 0.094 mW cm^−2^ at the position of the sample upon conversion of the number of photons in the white light at 532 nm.

The afterglow readout of pure organic materials requires excitation with strong UV or deep blue light. However, UV light or blue light is not always readily available. Developing a pure organic material that enables one to easily check afterglow information (by using common fluorescent tube lights or white LEDs in a mobile phone) will enable ubiquitous checking of afterglow information, i.e., ubiquitous afterglow readout. For example, when weak indoor light or sunlight acts as excitation light and the afterglow information from the medium can be determined visually in the dark with the area around the medium obscured with a hand, it is possible to obtain the afterglow information without a light source (**Figure** **8**a). Upon termination of the strong afterglow that is evident even under indoor light is produced after the white light emitted from the LED of a common mobile phone, there is no need to create darkness; afterglow information can be more easily confirmed without a special excitation light source or photodetector because there is no need to create darkness (Figure 8b). Unlike charge‐recombination‐type afterglow, which remains for a long time, organic persistent RTP can produce brighter afterglow by using a weak excitation light source.^[^ ^6^ ^]^ Therefore, there is the potential for clear afterglow recognition in a bright environment upon white LED excitation. To confirm these ubiquitous afterglow readouts, we compared **3d**‐doped and **6d**‐doped amorphous *β*‐estradiol (samples 1 and 2, respectively) as films exhibiting red persistent RTP with comparable RTP lifetimes (Figure 8c).^[^ ^14^ ^]^ Because samples 1 and 2 exhibit absorption <480 nm, they absorb light from 420–480 nm in common room light (Figure 8d). We created samples 1 and 2 exhibiting a star‐shaped persistent RTP pattern by photobleaching, and stacked samples 1 and 2 for evaluation (Figure 8e). Immediately upon termination of the UV light source excitation, because the excitation is sufficient the star‐shaped marks from samples 1 and 2 can be easily seen in the dark environment in the stacked sample (Figure 8f‐(i); Movie S1, Supporting Information). However, we observed star‐shaped red afterglow with unaided eyes only from sample 1 of the staked sample in a dark environment immediately upon termination of the white room light irradiation (Figure 8f‐(ii); Movie S2, Supporting Information). This is because the excitation by using room light is not large and the *Φ*_p_(RT) of sample 2 (≈2%) is much smaller than that of sample 1 (21%).^[^ ^14^ ^]^ Furthermore, because light from 420–480 nm is included in portable LEDs when we brought the LED close to the medium and turned off the LED, we observed a red afterglow with unaided eyes under the room light environment only from sample 1 of the stacked sample (Figure 8f‐(iii); Movie S3, Supporting Information). Therefore, a large *Φ*_p_(RT) at a long wavelength is crucial to extract ubiquitous afterglow readout. The ubiquitous afterglow readout, which is available under all normal circumstances, enables verification of high‐performance anticounterfeit functionality without any special additional tools.

## 3 Conclusion

Although researchers commonly obtain information regarding molecular conformations that include *θ* from single‐crystal structures, information on the conformation that includes dihedral angles beyond orientation in amorphous solid media is difficult to obtain. Our results indicate that the proposed statistical analysis regarding the correlation between the experimentally observed *k*_p_ and dynamically calculated *k*_p_, considering the thermal distribution of *θ*, is useful for obtaining the distribution of the dihedral angle, including symmetry information of the chromophores. Because the dihedral angle of chromophores doped into amorphous semiconductor hosts is also an active area of research (in the context of efficient and rapid thermally activated delayed fluorescence of chromophores in organic light‐emitting diodes),^[^ ^15^ ^]^ the proposed analysis is crucial for chromophores in optoelectronics applications. In addition, many D‐*π*‐D structures are used to enhance the S_1_–S_0_ transition dipole for highly efficient fluorescent emitters used in organic lasing^[^ ^16^ ^]^ and efficient two‐photon absorption (TPA) chromophores.^[^ ^17^ ^]^ However, the control of S*_n_*–S_0_ transition dipole has not been considered because the S*_n_*–S_0_ transition dipole is not related to fluorescence and TPA. Our proposed dynamic calculation procedure clarified a previously unknown promising science that activating the S*_n_*–S_0_ transition, facilitated due to symmetry‐breaking caused by thermally induced movement in the D‐*π*‐D structures, has a crucial role in selectively facilitating *k*_p_ without increasing *k*_nr_(RT) for enhancing RTP. Although thermally induced molecular motions and vibrations are often considered as factors that deactivate luminescence, our result reveals that there are molecular movements that enhance RTP. Therefore, investigating flexible motions for selective *k*_p_ enhancement by using dynamic quantum chemical calculations will be crucial to obtaining more efficient simple organic RTP emitters in long wavelengths. Because *Φ*_p_(RT) of the D‐*π*‐D structure (8.7% for **3 h)** is only 2.1% higher than that of the D‐*π* structure (6.6% for **2** **h**) in this paper, the difference might not be large. However, *Φ*_p_(RT) = 8.7% for **3** **h** is >1.3× larger than *Φ*_p_(RT) = 6.6% for **2** **h**. When long‐wavelength persistent RTP with a yield of >50% is expressed, a difference of >1.3× becomes a yield difference of ≥15%, which is not a small difference. Although D‐*π*‐D structures have elements that lead to top performance of long‐wavelength persistent RTP yield, this paper points out that such high‐performance molecules are overlooked because of the large underestimation of *k*_p_ in calculations using optimized D‐*π*‐D structures. Therefore, the estimation of conformation that takes into account thermally distributed symmetry‐breaking in solid films as indicated in this paper will help identify dyes that exhibit highly efficient persistent RTP at long wavelengths. Although improving persistent RTP yield in the long‐wavelength region is important in autofluorescence‐free bioimaging applications, it also enables efficient excitation with white light. Activation of persistent RTP by this white light excitation resulted in not only a dark afterglow readout activated by indoor white fluorescence but also a bright afterglow readout even under indoor fluorescent lighting by using the white light from an LED installed in a mobile phone. The ubiquitous bright afterglow readout, which operates with common white light, greatly expands the range of anti‐counterfeiting applications by using afterglow.

## 4 Experimental Section

### Materials

Chromophore **1** **h** (> 98.0%) and *β*‐estradiol (> 97.0%) were obtained from the Tokyo Chemical Industry Co., Ltd. (Japan) and synthesized **2** **h** as per the literature.^[^ ^4h^ ^]^ Syntheses of **3** **h** and **3d** were performed as Sections S1 and S2 (Supporting Information), and were characterized via proton nuclear magnetic resonance (^1^H NMR), carbon nuclear magnetic resonance (^13^C NMR) (ECA‐500, JEOL, Tokyo, Japan) spectroscopy, matrix‐assisted laser‐desorption ionization high‐resolution mass spectrometry (HRMS‐MALDI) (JMS‐S3000, JEOL), electrospray ionization high‐resolution mass spectroscopy (HRMS‐ESI) (JMS‐T100 AccuTOF, JEOL), and elemental analysis (Series II CHNS/O 2400 analyzer, PerkinElmer, MA, USA, and MT‐5, YANAKO, Tokyo, Japan) (Section S3, Supporting Information).

### Sample Preparation

A mixture (powder) of 99.7 wt.% *β*‐estradiol as a host and 0.3 wt.% **1h**–**3 h**, **3d**, or **4** **h** as a guest were heated to 200 °C to dissolve the guests into a melted *β*‐estradiol host. The melted material was sandwiched between two quartz substrates at 200 °C and quenched to RT to prepare amorphous *β*‐estradiol films doped with 0.3 wt.% **1h**–**3 h**, **3d**, or **4** **h** between two quartz substrates. For photophysical measurements under vacuum, a quartz substrate was peeled from the sandwiched samples, and amorphous films on a quartz substrate were used.

### Optical Measurements

The absorption spectra were measured by using an absorption photo‐chronometer (V‐670, JASCO, Tokyo, Japan). The steady‐state RT emission yield under vacuum (*Φ*_e_(RT)) was determined by using absolute photoluminescence quantum yield equipment (C9920‐02G, Hamamatsu Photonics, Shizuoka, Japan) (Figure S19a, Supporting Information). In the measurement, the emission signal (red line in Figure 2a) was determined by integrating photon signals in each wavelength from the sample before, under, and after ceasing excitation (Figure S19b, Supporting Information). To compare steady‐state emission intensity under excitation with afterglow emission intensity soon after ceasing excitation, the time change of emission spectral intensity before, under, and after ceasing excitation was measured by using monochromatic light from the excitation unit of a fluorimeter (FP‐8300, JASCO) as excitation and a photonic multichannel analyzer (C10027‐01, Hamamatsu Photonics) as a photodetector. The emission spectral intensity of steady‐state emission under excitation at 340 nm (A_1_) was compared with the emission spectral intensity of afterglow emission spectra soon (20–40 ms) after cessation of the 360‐nm excitation (A_2_) (Figure S19c, Supporting Information). *Φ*_p_(RT) was determined from *Φ*_p_(RT) = *Φ*_e_(RT)A_2_/A_1_. The fluorescence yield at RT (*Φ*_f_(RT)) was determined as *Φ*_e_(RT) − *Φ*_p_(RT). Reference ^[^ ^3e^ ^]^ summarizes the logical reasonability of the determination procedure of *Φ*_p_(RT). The temperature dependence of fluorescence and phosphorescence characteristics as per a cryostat (Optistat DN‐V, Oxford Instruments, Abingdon‐on‐Thames, UK) was used to change the temperature. The fluorescence intensity change and phosphorescence intensity change were recorded by changing the temperature. *Φ*_f_(T) and *Φ*_p_(T) were determined by comparing the emission intensities at T K with those at RT. The yield of intersystem crossing from S_1_ to triplet states (*Φ*_isc_) was determined by using transient absorption techniques. Transient absorption measurements were performed with a sub‐nanosecond transient absorption spectrophotometer (picoTAS, Unisok, Osaka, Japan) equipped with a 355‐nm, Q‐switched microchip laser (PNV‐M02510‐1×0, Teem Photonics, Meylan, France). Section S6 (Supporting Information) provides a more detailed procedure.

### Quantum Chemical Calculations

A geometry of T_1_ was optimized by using DFT based on Gaussian09 with the B3LYP functional and 6–31G(d) basis set. Vibrational information of T_1_ geometry was calculated by DFT based on Gaussian09 with the B3LYP functional and 6–31G(d) basis set. For each geometry depending on vibrations from T_1_, the SOC and transition dipole moment were calculated by using the Amsterdam Density Functional (ADF) 2018 package. In the SOC calculations, the SOC operator within the zeroth‐order regular approximation was ${\hat{H}}_{\mathrm{SO}}$. The SOC parameter between the *n*th‐order singlet excited state and the *m*th‐order triplet excited state $\left\langle S_{n}|{\hat{H}}_{\mathrm{SO}}|T_{m}\right\rangle$ was treated as a perturbation based on the scalar relativistic orbitals, with the PBE0 functional and TZP basis set. Detailed information is explained in Section S9 (Supporting Information).

## Conflict of Interest

The authors declare no conflict of interest.

## Supporting information

Supporting Information

Supplemental Movie 1

Supplemental Movie 2

Supplemental Movie 3

Supporting cif

## Data Availability

The data that support the findings of this study are available in the supplementary material of this article.
