# Supplementary material for: Symmetry‐Breaking Triplet Excited State Enhances Red Afterglow Enabling Ubiquitous Afterglow Readout
Source: Adv Sci (Weinh). 2024 Feb 4;11(14):2308897. doi: 10.1002/advs.202308897 (PMC11005713; doi:10.1002/advs.202308897)
Supplement: Supplementary file 1 — Supporting Information [file ADVS-11-2308897-s001.pdf]

ADVANCED SCIENCE Open Access

Supporting Information

for *Adv. Sci.*, DOI 10.1002/advs.202308897

Symmetry-Breaking Triplet Excited State Enhances Red Afterglow Enabling Ubiquitous Afterglow Readout

*Bahadur Sk and Shuzo Hirata**

Supporting Information

Symmetry-breaking triplet excited state enhances red afterglow enabling ubiquitous afterglow readout

*Bahadur Sk, and Shuzo Hirata**

*Corresponding author. E-mail: shuzohirata@uec.ac.jp

Contents

Supporting Text
- **Section S1. Synthesis and characterizations of chromophores**
- **Section S2. Single-crystal X-ray diffraction analysis**
- **Section S3. NMR and mass spectra**
- **Section S4. Optical measurements**
- **Section S5. Optical properties in amorphous *β*-estradiol host**
- **Section S6. Determination of Φ_{isc}**
- **Section S7. Optical properties in polymer hosts**
- **Section S8. Effect of k_{nr} on change of dihedral angle of D-π-D structure**
- **Section S9. Quantum chemical calculations**
- **Section S10. Ubiquitous afterglow readout**
- **Section S11. Supporting movies**
- **Section S12. References**

Figures S1 to S45

Tables S1 to S7

Movies S1 to S3

References (1 to 16)

Section S1. Synthesis and characterizations of chromophores

Synthesis of 2,10-dibromodibenzo[*g*,*p*]chrysene (2):

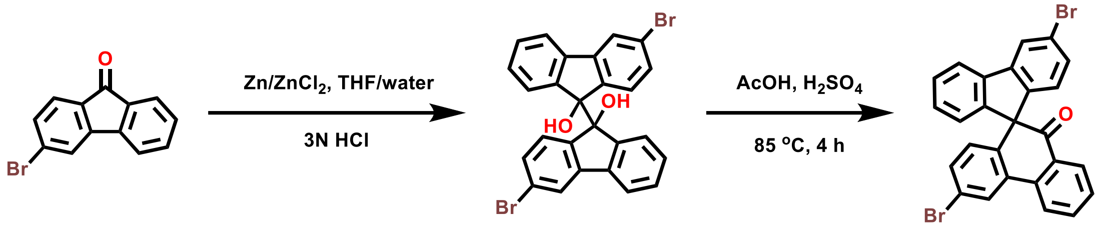

1

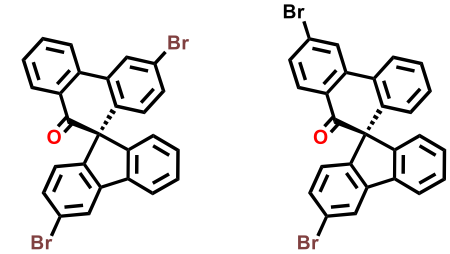

spiro-1 (1) spiro-2

Scheme S1. Synthetic scheme for 3,6'-dibromo-10'*H*-spiro[fluorene-9,9'-phenanthren]-10'-one (Spiro-1, **1**).

First, 2,10-dibromodibenzo[*g*,*p*]chrysene (**2**) was synthesized as per the literature.[S1,S2] Next, 3-bromo-9-fluorenone (0.50 g, 1.9 mmol, 1.0 eqv.), zinc powder (3.2 g, 48 mmol, 25 eqv.) and zinc chloride (0.53 g, 3.9 mmol, 2.0 eqv.) were dissolved in 50% aqueous tetrahydrofuran (THF) (15 mL), and the reaction mixture was stirred at room temperature for 4 h. Then, 3 mL of 3 M hydrogen chloride (HCl) was added to the reaction mixture, and the Zn powder was removed by filtration. The filtrate was extracted with toluene (3 × 20 mL). The combined organic phase was washed with water (2 × 30 mL) and brine solution (2 × 30 mL). The organic phase was dried over anhydrous sodium sulfate (Na_2SO_4), and the solvent was evaporated under vacuum. The crude product of 3,3'-dibromo-9*H*,9'*H*-[9,9'-bifluorene]-9,9'-diol was purified by short silica gel column chromatography (20% ethyl acetate/hexane, 0.45 g, 0.86 mmol, 89% yield) and directly used for the next step. The mixture of 3,3'-dibromo-9*H*,9'*H*-[9,9'-bifluorene]-9,9'-diol (0.45 g, 0.86 mmol), acetic acid (AcOH) (10 mL), and sulfonic acid (H_2SO_4) (0.50 mL) was stirred at 85°C for 4 h. Then, the reaction mixture was cooled to room temperature and combined with cold water (40 mL). The organic layer was collected in dichloromethane (DCM) (3 × 50 mL) and further washed with water (2 × 60 mL) and brine solution (2 × 40 mL). The organic phase was dried over anhydrous Na_2SO_4 and the solvent was evaporated under vacuum. Because of the equal ratio of the spiro-1 (3,6'-dibromo-10'*H*-spiro[fluorene-9,9'-phenanthren]-10'-one) and spiro-2 (3,3'-dibromo-10'H-spiro[fluorene-9,9'-phenanthren]-10'-one) as well as their different R_f values in the toluene/hexane mixture (R_f = 0.65 for spiro-1 and 0.55 for spiro-2), the isomers were separated by silica gel column chromatography with a 20% toluene/hexane mixture as the eluent. The spiro-1 (3,6'-dibromo-10'*H*-spiro[fluorene-9,9'-phenanthren]-10'-one) (**1**) was obtained at ca. 90 mg (0.18 mmol, yield = 21%). ^{1}H NMR (500 MHz, chloroform-*d*, δ): 8.33 (d, *J* = 1.8 Hz, 1H), 8.04 (dd, *J* = 8.0, 1.2 Hz, 1H), 7.92 (d, *J* = 1.8 Hz, 1H), 7.83 (d, *J* = 8.2 Hz, 1H), 7.75 (dt, *J* = 7.6, 0.9 Hz, 1H), 7.58 (dd, *J* = 8.3, 1.8 Hz, 1H), 7.43 – 7.37 (m, 2H), 7.33 (dd, *J* = 8.1, 1.9 Hz, 1H), 7.20 (td, *J* = 7.6, 1.1 Hz, 1H), 7.15 – 7.10 (m, 1H), 7.00 (dt, *J* = 7.7, 0.9 Hz, 1H), 6.92 (d, *J* = 8.1 Hz, 1H), 6.61 (dd, *J* = 7.9, 1.3 Hz, 1H); ^{13}C NMR (126 MHz, chloroform-*d*, δ): 195.89, 147.14, 145.69, 144.01, 140.24, 139.80, 139.09, 131.70, 130.98, 130.76, 130.35, 130.14, 129.31, 128.84, 128.76, 128.60, 128.54, 128.28,

126.65, 126.37, 124.74, 124.50, 123.98, 122.83, 121.07, 68.33; HRMS (ESI) *m/z*: calculated for $C_{26}H_{14}Br_2ONa$ $[M+Na]^+$ is 524.929 and found 524.930.

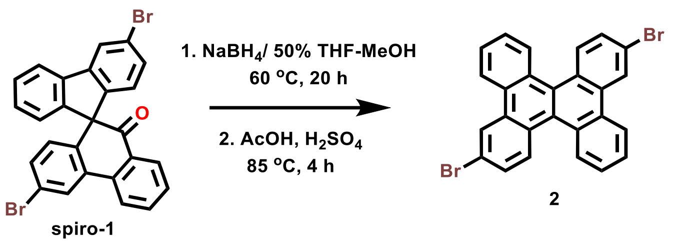

Scheme S2. Synthetic scheme for 2,10-dibromodibenzo[*g*,*p*]chrysene (**2**).

Spiro-1 (80 mg, 0.16 mmol, 1.0 eqv.) was dissolved in THF (3 mL) and methanol (MeOH) (3 mL) in a three-neck round-bottom flask under nitrogen. The reaction mixture was heated to 65°C, sodium borohydride ($NaBH_4$) (7.0 mg, 0.19 mmol, 1.2 eqv.) was added in three portions (~2 mg each time) over 4 h with heating, and the reaction was monitored by thin-layer chromatography (TLC). After completion, the reaction mixture was cooled to ice-cold condition and quenched by using 5 mL of 1 M HCl. The product was then extracted with DCM (3 × 10 mL). The combined organic extracts were washed with water (2 × 10 mL) and saturated brine (20 mL). The organic phase was then dried over anhydrous Na_2SO_4, filtered, and the solvent was removed under reduced pressure. The product was isolated as a white solid which was purified by recrystallization from hexane and directly used for the next step. The mixture of 3,6'-dibromo-10'*H*-spiro[fluorene-9,9'-phenanthren]-10'-ol, AcOH (8 mL), and H_2SO_4 (0.2 mL) was set for stirring at 85°C for 4 h. Next, the reaction mixture was cooled to room temperature and combined with cold water (30 mL). The reaction mixture was neutralized by using 10% aq. sodium hydrogen carbonate. Then, the organic layer was collected in DCM (3 × 20 mL) and dried by using Na_2SO_4. The crude product was then purified by silica gel column chromatography [10% DCM/hexane] to afford the colorless compound **2** (54 mg, 0.11 mmol, yield = 70%). ^{1}H NMR (500 MHz, chloroform-*d*, δ): 8.82 (d, *J* = 2.0 Hz, 2H), 8.69 – 8.41 (m, 6H), 7.74 – 7.63 (m, 6H); ^{13}C NMR (126 MHz, chloroform-*d,* δ): 132.59, 130.32, 129.83, 129.47, 129.00, 127.64, 127.45, 127.20, 126.64, 123.79, 121.14; HRMS (MALDI) *m/z*: calculated for $C_{26}H_{14}Br_2$ $[M]^+$ is 485.944 and found 485.943; Elemental analysis calculated for $C_{26}H_{14}Br_2$: C = 64.23, H = 2.90; found: C = 64.39, H = 3.04. We confirmed substitution of bromine at the 2 and 10 positions of DBC in **2** by using single-crystal X-ray diffraction analysis (Table S1).

Synthesis of 2,10-di(10H-phenoxazin-10-yl)dibenzo[*g*,*p*]chrysene(3h):

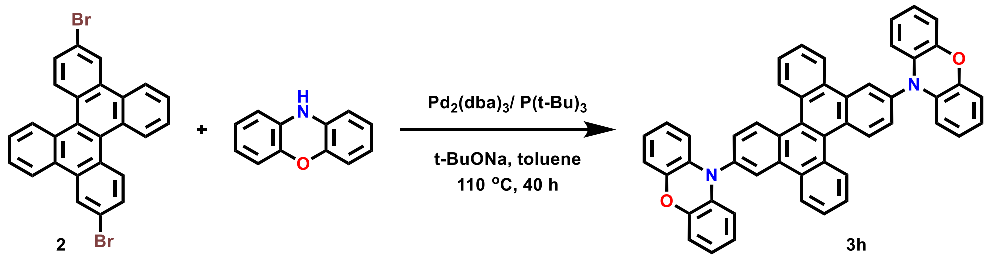

Scheme S3. Synthetic scheme for 2,10-di(10*H*-phenoxazin-10-yl)dibenzo[*g*,*p*]chrysene (**3h**).

Toluene (6 mL) was added to a mixture of 2,10-dibromodibenzo[*g*,*p*]chrysene (30 mg, 0.062 mmol, 1.0 eqv.), phenoxazine (25 mg, 0.14 mmol, 2.2 eqv.), Tris(dibenzylideneacetone)dipalladium(0) ($Pd_2(dba)_3$) (1.51 mg, 0.00160 mmol, 0.040 eqv.), sodium *tert*-Butoxide (*t*-BuONa) (6 mg, 0.06 mmol, 1 eqv.), and tri-*tert*-butylphosphine [P(*t*-

butyl)$_3$] (0.13 mg, 0.00064 mmol, 0.010 eqv.) in a glove box. The solution was stirred under nitrogen at 110°C for 40 h. After cooling to room temperature, the reaction was quenched by adding 20 mL of distilled water, and the organic layer was then extracted with DCM (3 × 10 mL). The collected organic products were then dried over anhydrous Na_2SO_4 and concentrated under vacuum. The crude product was then purified by silica gel column chromatography (20% DCM/hexane). The yellow solid (19 mg, 0.027 mmol) as **3h** was obtained with a yield = 44%. Because the TLC spot of phenoxazine and product is close, the yellow solid product was further purified by 3× reprecipitation in hot methanol followed by sublimation. ^{1}H NMR (500 MHz, chloroform-*d,* δ): 8.96 (d, *J* = 8.7 Hz, 2H), 8.78 – 8.71 (m, 4H), 8.66 (dt, *J* = 7.0, 2.3 Hz, 2H), 7.75 – 7.68 (m, 4H), 7.63 (dd, *J* = 8.6, 2.0 Hz, 2H), 6.78 – 6.57 (m, 12H), 6.08 (d, *J* = 8.1 Hz, 4H); ^{13}C NMR (126 MHz, chloroform-*d,* δ) 144.14, 137.49, 134.58, 133.50, 132.11, 130.40, 129.23, 128.99, 128.95, 128.85, 127.82, 127.56, 127.26, 126.24, 124.07, 123.44, 121.64, 115.68, 113.54; HRMS (ESI) *m/z*: calculated for $C_{50}H_{30}N_2O_2$ [M+H]$^+$: 691.239; found: 691.241. Elemental analysis calculated for $C_{50}H_{30}N_2O_2$: C = 86.94, H = 4.38, N = 4.06; found: C = 86.66, H = 4.47, N = 4.20.

Deuteration of dibenzo[g,p]chrysene (DBC-d$_{16}$):

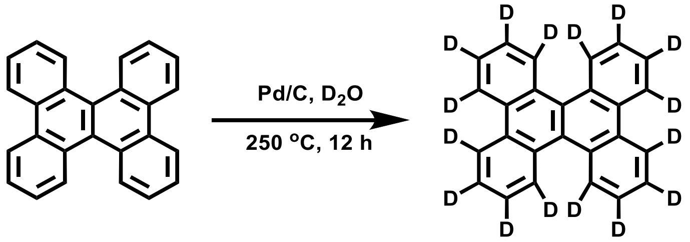

Scheme S4. Synthetic scheme for deuterated dibenzo[*g*,*p*]chrysene (DBC-d$_{16}$).

Dibenzo[*g,p*]chrysene (1056 mg, 3.22 mmol), 10 wt% palladium on activated carbon (125 mg), and D_2O (25 mL) were added to a 50-mL Teflon-lined autoclave; and heated to 250°C for 12 h. The internal pressure of the reaction mixture was 4–5 MPa at 250°C. The mixture was then cooled to RT and the organic layer was collected in DCM by filtration. After drying over Na_2SO_4, DCM was removed to give to a paled yellow powder (1059 mg). The pale yellow powder (1051 mg), 10 wt% palladium on activated carbon (125 mg), and D_2O (25 mL) were added to a 50-mL Teflon-lined autoclave; and heated to 250°C for 12 h. The internal pressure of the reaction mixture was 4–5 MPa at 250°C. The mixture was then cooled to RT, and the organic layer was collected in DCM by filtration. After drying over Na_2SO_4, DCM was removed to give a pale yellow powder (1020 mg, 2.99 mmol) as a dibenzo[*g*,*p*]chrysene-d$_{16}$. The deuteration reaction was performed 2× with an overall reaction yield of 93%. HRMS (MALDI) *m/z*: calculated for $C_{26}H_{14}Br_2$ [M]$^+$: 344.226; found: 341.212 (Figure S10). Deuteration yield (79%) was confirmed by ^{1}H NMR analysis (Figure S11).

Synthesis of deuterated 2,10-dibromodibenzo[g,p]chrysene (2'):

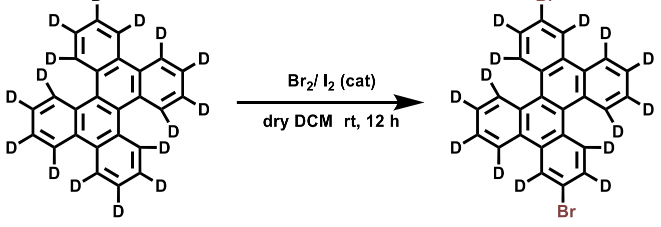

2'

Scheme S5. Synthetic scheme for deuterated 2,10-dibromodibenzo[*g*,*p*]chrysene (**2'**).

In a 25-mL sealed tube, DBC-d_{16} (100 mg, 0.293 mmol, 1.00 eqv.) was dissolved in anhydrous DCM (4 mL). Iodine (7.37 mg, 0.0290 mmol, 0.0991 eqv.) was added, and the mixture was stirred for 30 min at room temperature. Bromine (92.8 mg, 0.581 mmol, 1.98 eqv.) dissolved in 1 mL DCM was added slowly by using a syringe and stirring was continued for 2 h at room temperature. The white solid precipitate was obtained after complete consumption of bromine. The precipitate was filtered, washed with methanol, and dried. The crude product (175 mg) was then purified by silica gel column chromatography (10% DCM/hexane) to afford **2'** (120 mg, yield = 82.4%). HRMS–MALDI calculated for $C_{26}D_{14}Br_2$ is 500.291 and found 497.004 (Figure S12). Product **2'** was further purified by temperature gradient sublimation. The needle-like crystals of **2'** was obtained during sublimation, and X-ray diffraction analysis indicates that the needle crystals were 2,10-dibromodibenzo[*g*,*p*]chrysene-d_{14} (Table S1). However, some small randomly shaped nano-micro crystals were also observed at the same temperature and pressure, possibly due to the isomer impurity. To avoid any isomer impurity, we collected the needle-like crystals and performed the next reaction to obtain **3d** chromophore.

Synthesis of 10,10'-(dibenzo[*g*,*p*]chrysene-2,10-diyl-d14)bis(10*H*-phenoxazine) (3d):

Toluene (10 mL) was added to a mixture of 2,10-dibromodibenzo[*g*,*p*]chrysene-d_{14} (20 mg, 0.040 mmol, 1.0 eqv.), phenoxazine (18 mg, 0.098 mmol, 2.4 eqv.), $Pd_2(dba)_3$ (1.5 mg, 0.0016 mmol, 0.040 eqv.), *t*-BuONa (3.8 mg, 0.040 mmol, 0.99 eqv.), and P(*t*-butyl)$_3$ (0.08 mg, 0.0004 mmol, 0.01 eqv.) in a glove box. The solution was stirred under nitrogen at 110°C for 46 h. After cooling to room temperature, the reaction was quenched by adding 15 mL of distilled water. The organic layer was then extracted with DCM (3 × 10 mL). The collected organic products were then dried over anhydrous Na_2SO_4 and concentrated under vacuum. The crude product was then purified by silica gel column chromatography (20% DCM/hexane) to give a yellow powder (12 mg, 43%). The product was further purified by reprecipitation in hot methanol, followed by sublimation. HRMS (MALDI) *m/z*: calculated for $C_{50}H_{16}D_{14}N_2O_2$ $[M]^+$: 704.319; found: 701.294 (Figure S13). Deuteration yield (75%) of deuterated parts was confirmed by ^{1}H NMR analysis (Figure S14).

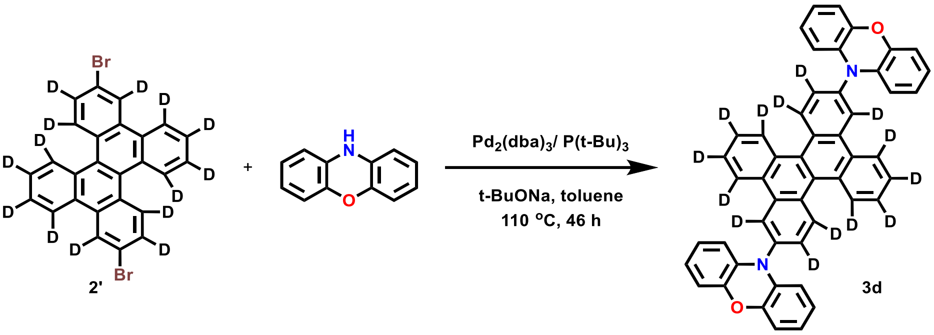

Scheme S6. Synthetic scheme for 10,10'-(dibenzo[*g*,*p*]chrysene-2,10-diyl-d14)bis(10*H*-phenoxazine) (**3d**).

Synthesis of 4,4'-di(10*H*-phenoxazin-10-yl)-1,1'-biphenyl (4h):

Toluene (15 mL) was added to a mixture of 4,4'-dibromo-1,1'-biphenyl (200 mg, 0.641 mmol, 1.00 eqv.), phenoxazine (258 mg, 1.41 mmol, 2.20 eqv.), $Pd_2(dba)_3$ (11.7 mg, 0.0128 mmol, 0.0200 eqv.), *t*-BuONa (61 mg, 0.64 mmol, 0.99 eqv.), and tri-*tert*-butylphosphine [P(*t*-butyl)$_3$] (1.3 mg, 0.0064 mmol, 0.010 eqv.) in a glove box. The solution was stirred under nitrogen at 110°C for 48 h. After cooling to room temperature, the reaction was quenched by adding 30 mL of distilled water, and the organic layer was then extracted with DCM (3 × 20 mL). The collected organic products were then dried over anhydrous Na_2SO_4 and concentrated under

vacuum. The crude product was then purified by silica gel column chromatography (20% DCM/hexane). The

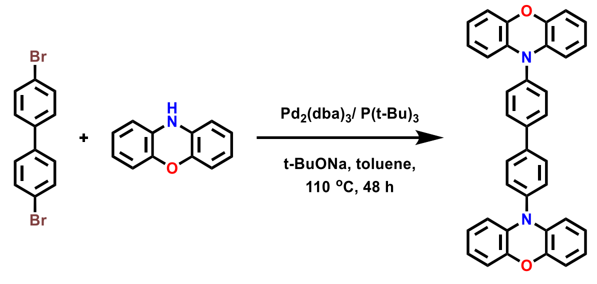

Scheme S7. Synthetic scheme for 4,4'-di(10*H*-phenoxazin-10-yl)-1,1'-biphenyl (**4h**).

light-yellow solid (74.8 mg, 0.145 mmol) was obtained with a yield of 22.6%. The product was further purified by reprecipitation in hot methanol and sublimed. ^{1}H NMR (500 MHz, chloroform-*d,* δ) 7.88 (d, *J* = 8.2 Hz, 4H), 7.50 – 7.43 (m, 4H), 6.75 – 6.57 (m, 12H), 6.01 (d, *J* = 7.7 Hz, 4H); ^{13}C NMR (126 MHz, chloroform-*d*, δ) 144.11, 140.34, 138.73, 134.41, 131.47, 129.84, 123.35, 121.54, 115.61, 113.37; HRMS (ESI) *m/z*: calculated for $C_{36}H_{24}N_2O_2$ $[M]^+$: 516.184; found: 516.185. Elemental analysis calculated for $C_{36}H_{24}N_2O_2$: C = 83.70, H = 4.68, N = 5.12; found: C = 83.64, H = 4.65, N = 5.23.

Calculation of deuteration yield via NMR spectroscopy:

The DBC-d_{16} deuteration yield was analyzed by ^{1}H NMR spectroscopy.[S3,S4] A comparison of deuterated and nondeuterated ^{1}H peak widths in the presence of a dibromoethane ($C_2H_4Br_2$) reference was performed. A stock solution of $C_2H_4Br_2$ in chloroform-d ($CDCl_3$) was prepared. Then, the same quantities of DBC and DBC-d_{16} were dissolved in the stock solution, and placed in the NMR tube. Figure S11 shows the data that were used to determine the deuteration yield of dibenzo[*g*,*p*]chrysene-d_{16}.

Section S2. Single-crystal X-ray diffraction analysis

Single colorless needle-shaped crystals of 2,10-dibromodibenzo[*g*,*p*]chrysene (**2**) and deuterated 2,10-dibromodibenzo[*g*,*p*]chrysene (**2'**) were obtained by slow evaporation from chloroform and sublimation, respectively. A suitable crystal 0.68 mm × 0.04 mm × 0.03 mm for **2** and 0.55 mm × 0.03 mm × 0.03 mm for **2'** was selected, and mounted on a suitable support on an XtaLAB Synergy R, DW system, HyPix diffractometer (Rigaku, V1.171.42.72a, 2022). The crystal was kept at a steady T = 301(2) K and 298.7(4)K during data collection for **2** and **2'**, respectively. The structure was solved with the ShelXT 2018/2 (Sheldrick, 2018) structure solution program by using the intrinsic phasing solution method and by using Olex2 as the graphical interface.[S5] The model was refined with version 2018/3 of ShelXL 2018/3 (Sheldrick, 2015) by using the least-squares minimization. Table S1 shows the parameters for the crystal data.

Table S1. Crystallographic data table for **2** and **2'**.

Compound	2	2'
Chemical formula	$C_{26}H_{14}Br_2$	$C_{26}D_{14}Br_2$
Formula weight	486.19	500.28
Temperature/K	301(2)	298.7(4)
Crystal system	monoclinic	monoclinic
Space group	$P2_1/c$	$P2_1/c$
a,/Å	12.3082(8)	12.5050(12)
b/Å	19.5021(11)	19.3398(10)
c/Å	7.9005(6)	7.8883(6)
α/°	90	90
β/°	101.585(7)	101.565(8)
γ/°	90	90
Volume/Å^3	1857.8(2)	1869.0(3)
Z	4	4
ρ_{calc}g/cm^3	1.738	1.778
μ/mm^{-1}	4.373	4.346
F(000)	960	960
Radiation	Mo Kα (λ = 0.71073)	Mo Kα (λ = 0.71073)
2θ range for data collection/°	3.972 to 63.444	5.366 to 63.434
Index ranges	$-17 \le h \le 15$, $-28 \le k \le 24$, $-11 \le l \le 10$	$-16 \le h \le 16$, $-23 \le k \le 28$, $-10 \le l \le 11$
Reflections collected	13948	19023
Independent reflections	5118 [R_{int} = 0.0623, R_{sigma} = 0.0657]	5160 [R_{int} = 0.0805, R_{sigma} = 0.0976]
Data/restraints/parameters	5118/0/253	5160/0/253
Goodness-of-fit on F^2	0.94	1.027
Final R indexes [I>=2σ (I)]	R_1 = 0.0638, wR_2 = 0.1631	R_1 = 0.0944, wR_2 = 0.2583
Final R indexes [all data]	R_1 = 0.1301, wR_2 = 0.1937	R_1 = 0.2099, wR_2 = 0.3208
Largest diff. peak/hole / e Å^{-3}	0.54/-0.54	3.44/-0.60
CCDC	2270771	2270770

Section S3. NMR and mass Spectra

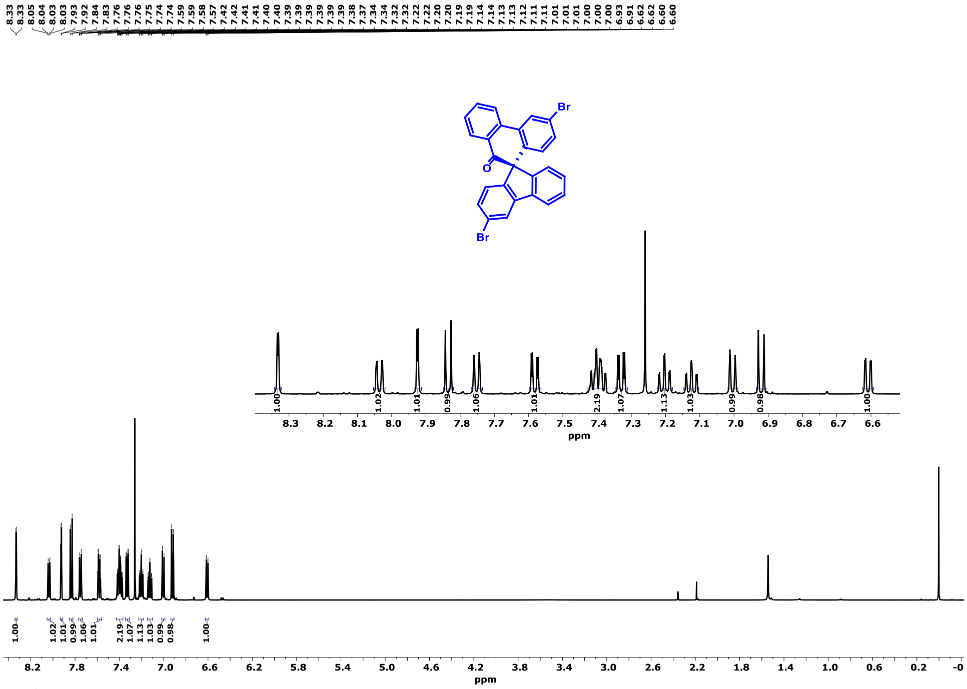

Figure S1. ^{1}H NMR spectrum of 3,6'-dibromo-10'*H*-spiro[fluorene-9,9'-phenanthren]-10'-one (**1**) in $CDCl_3$.

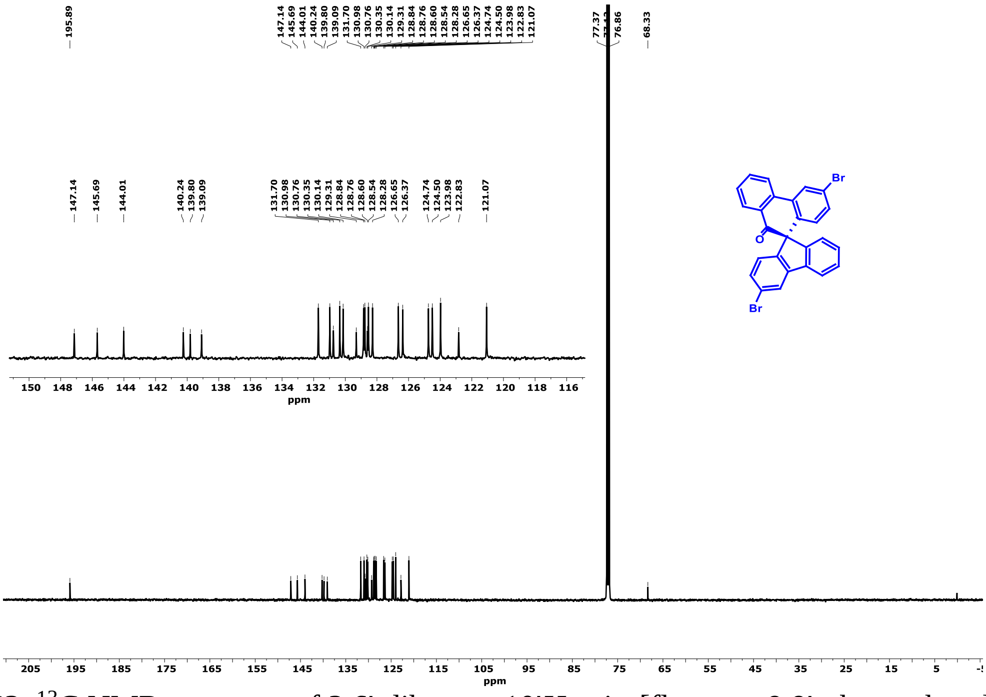

Figure S2. ^{13}C NMR spectrum of 3,6'-dibromo-10'*H*-spiro[fluorene-9,9'-phenanthren]-10'-one (**1**) in $CDCl_3$.

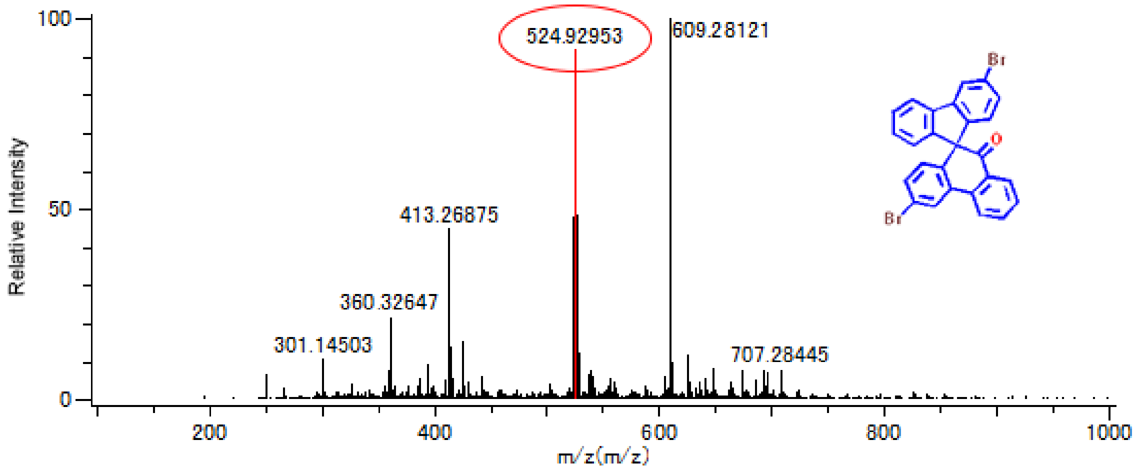

Figure S3. HRMS–ESI mass spectrum of 3,6'-dibromo-10'*H*-spiro[fluorene-9,9'-phenanthren]-10'-one (**1**).

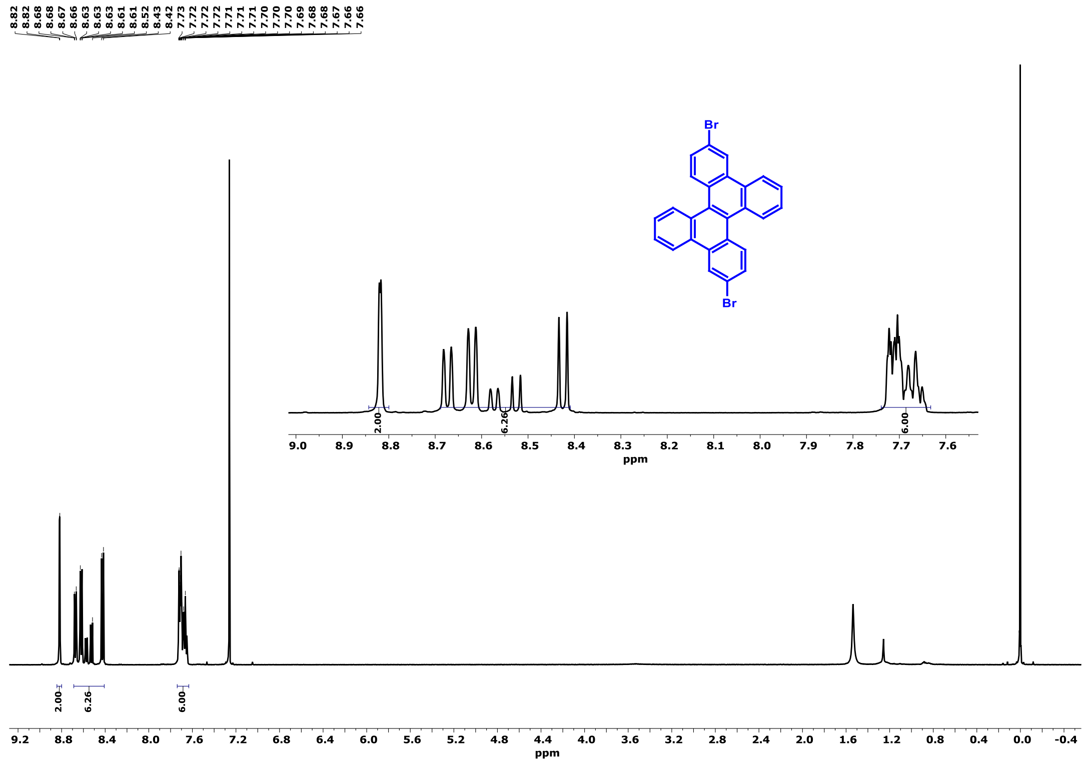

Figure S4. ^{1}H NMR spectrum of 2,10-dibromodibenzo[*g*,*p*]chrysene (**2**) in $CDCl_3$.

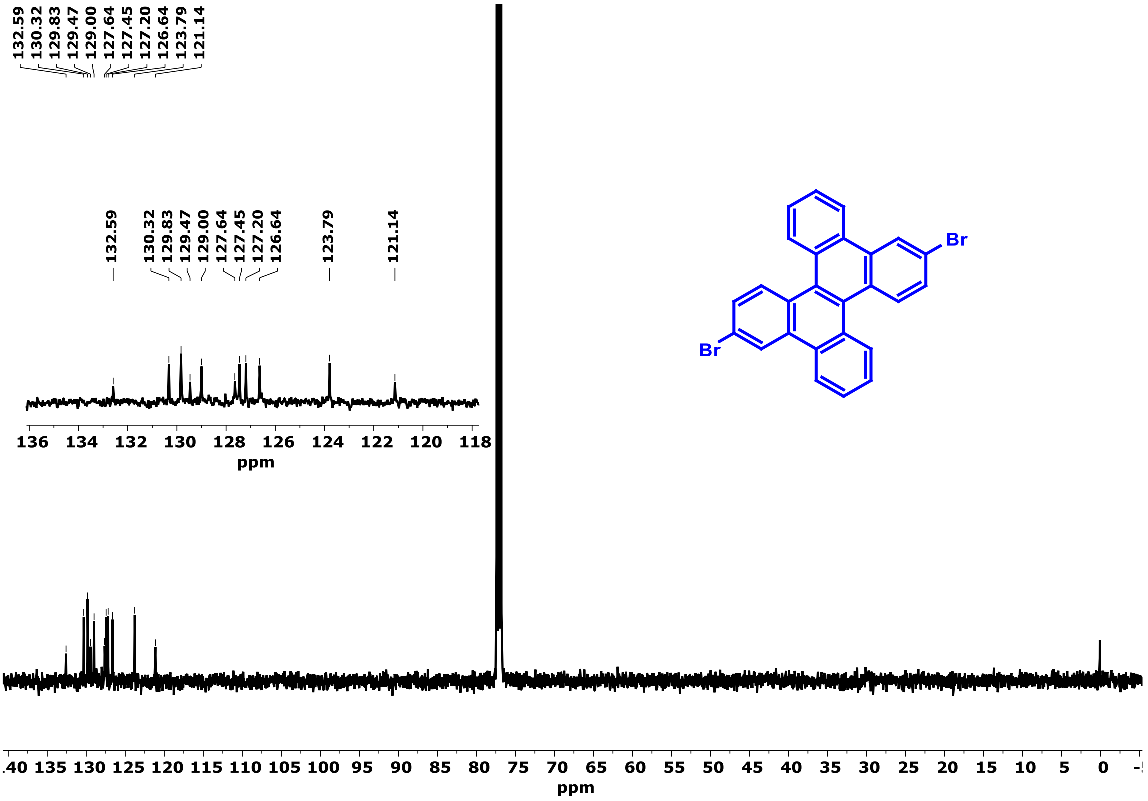

Figure S5. ^{13}C NMR spectrum of 2,10-dibromodibenzo[*g*,*p*]chrysene (**2**) in $CDCl_3$.

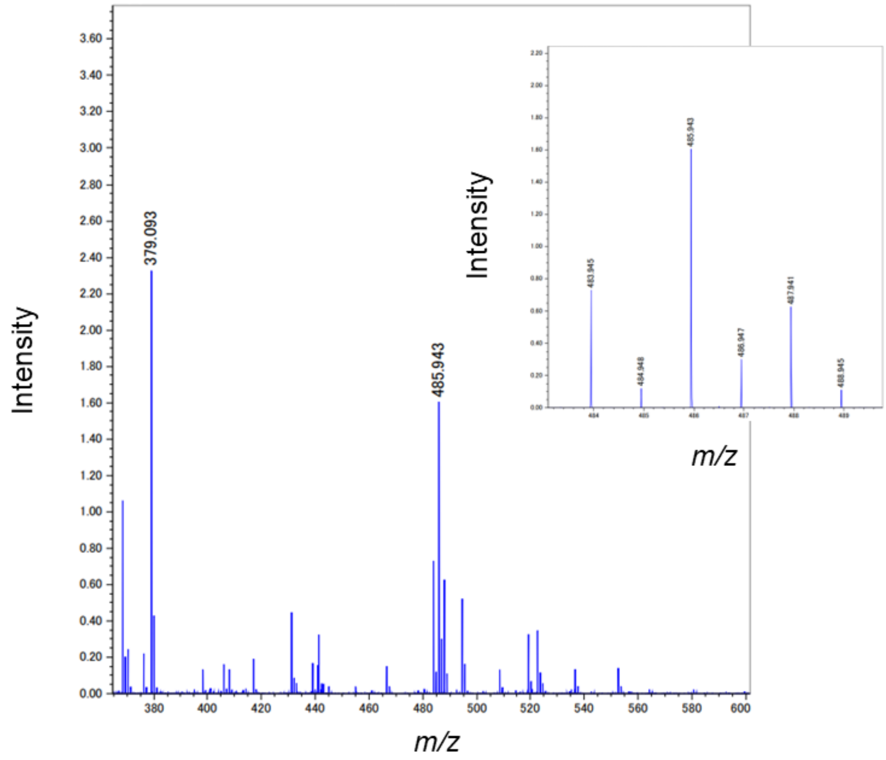

Figure S6. HRMS–MALDI mass spectrum of 2,10-dibromodibenzo[*g*,*p*]chrysene (**2**).

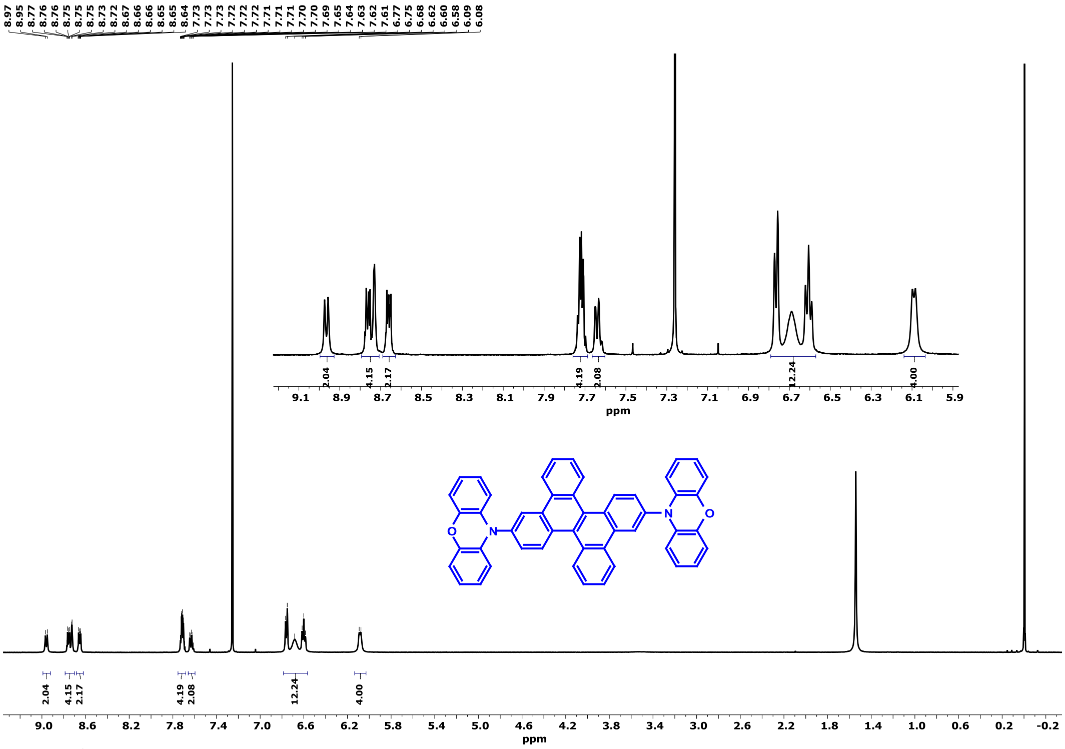

Figure S7. ^{1}H NMR spectrum of 2,10-di(10*H*-phenoxazin-10-yl)dibenzo[*g*,*p*]chrysene (**3h**) in $CDCl_3$.

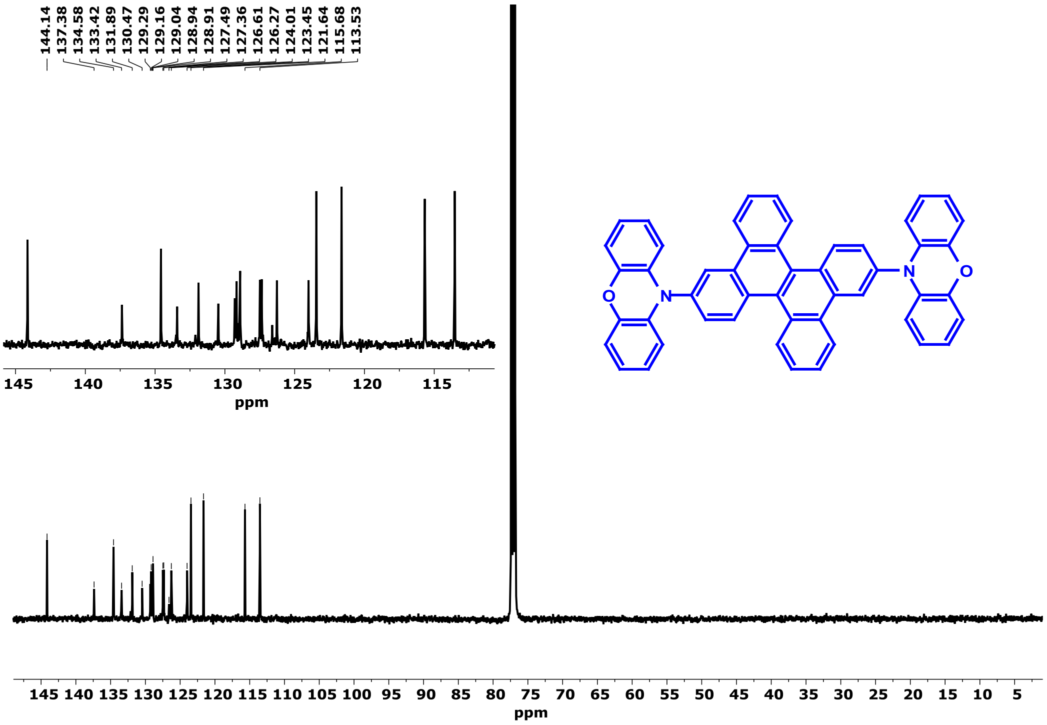

Figure S8. ^{13}C NMR spectrum of 2,10-di(10H-phenoxazin-10-yl)dibenzo[*g*,*p*]chrysene (**3h**) in $CDCl_3$.

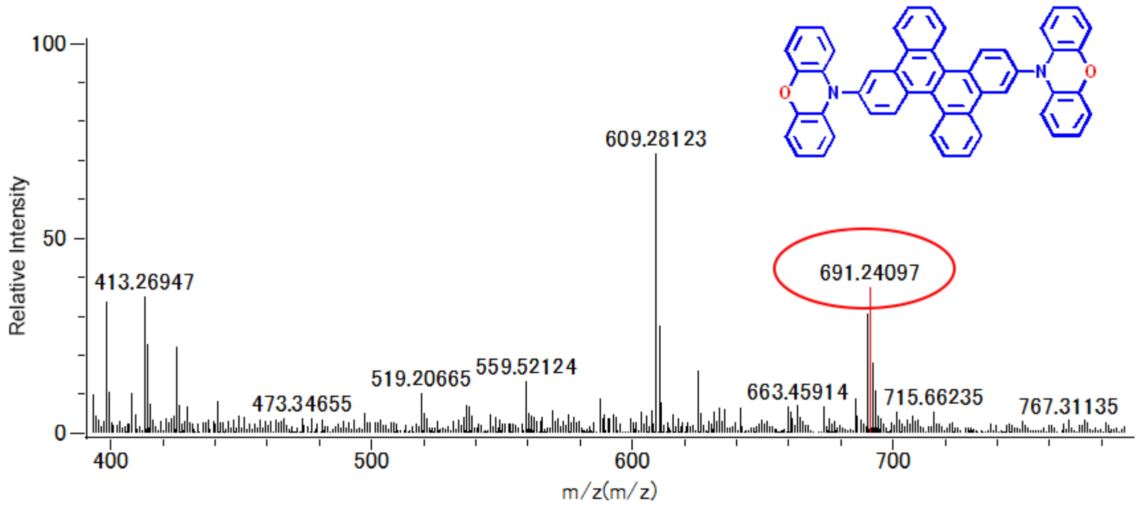

Figure S9. HRMS–ESI mass spectrum of 2,10-di(10H-phenoxazin-10-yl)dibenzo[*g*,*p*]chrysene (**3h**).

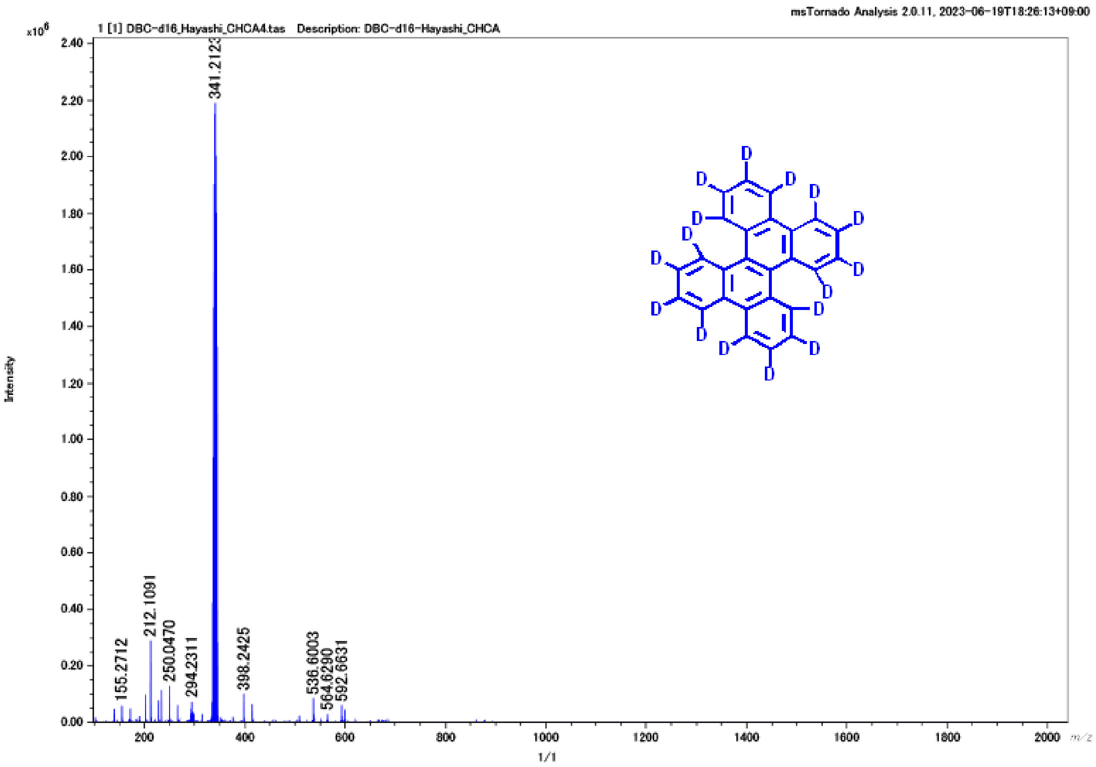

Figure S10. HRMS–MALDI mass spectrum of dibenzo[*g*,*p*]chrysene-d_{16}.

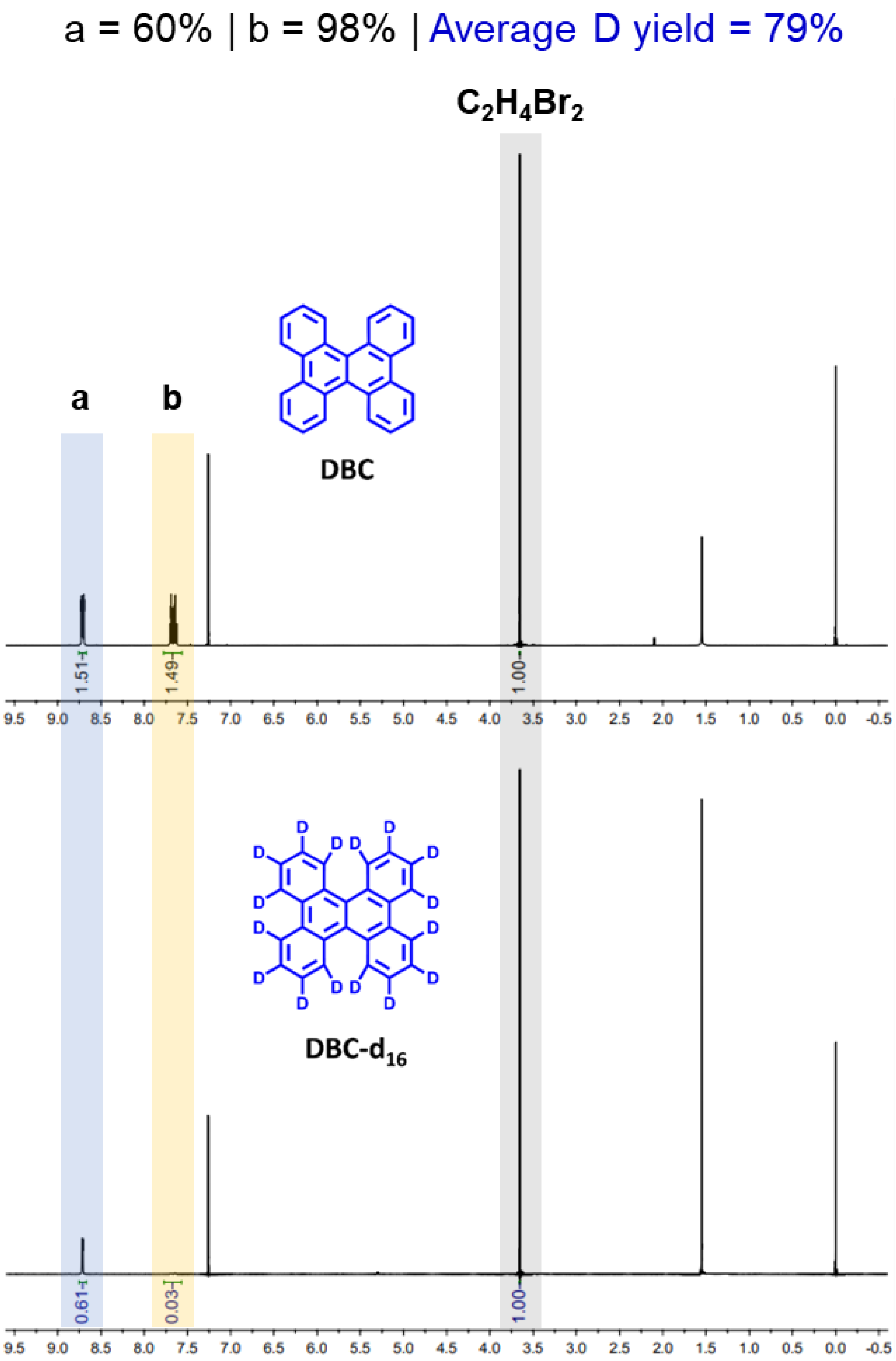

Figure S11. Calculation of deuteration yield of DBC-d_{16} by ^{1}H NMR spectroscopy. ^{1}H NMR spectra of DBC and DBC-d_{16} in presence of reference $C_2H_4Br_2$ in $CDCl_3$. The $C_2H_4Br_2$ protons were chosen as the reference for calculating the deuteration yield of DBC-d_{16} and found 79%.

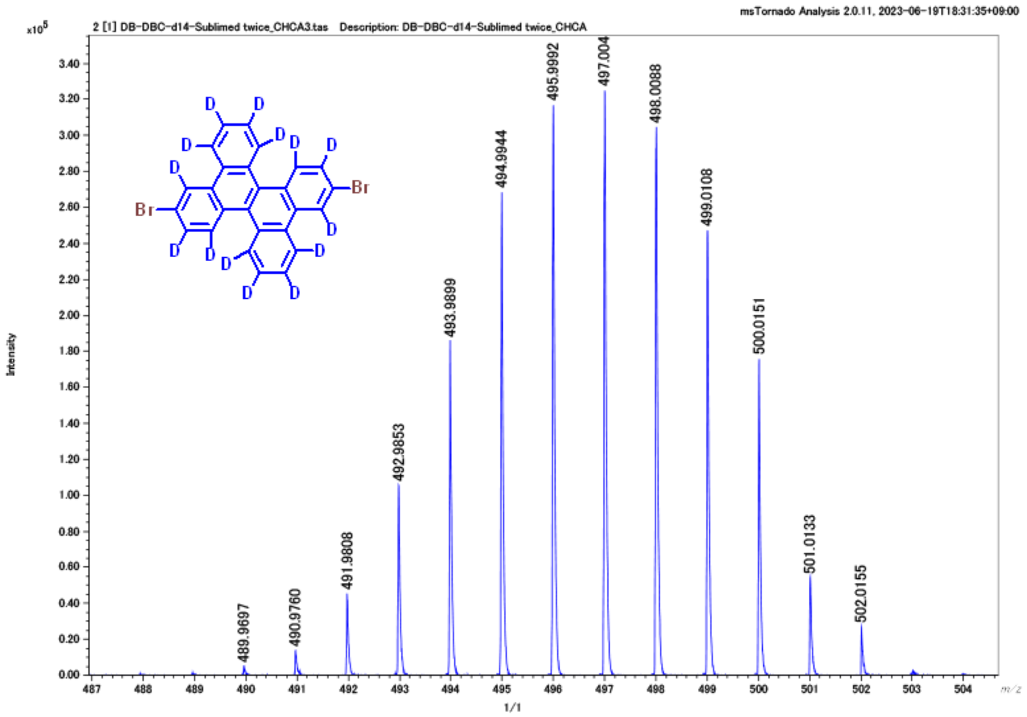

Figure S12. HRMS–MALDI mass spectrum of 2,10-dibromodibenzo[*g*,*p*]chrysene-d_{14} (**2'**).

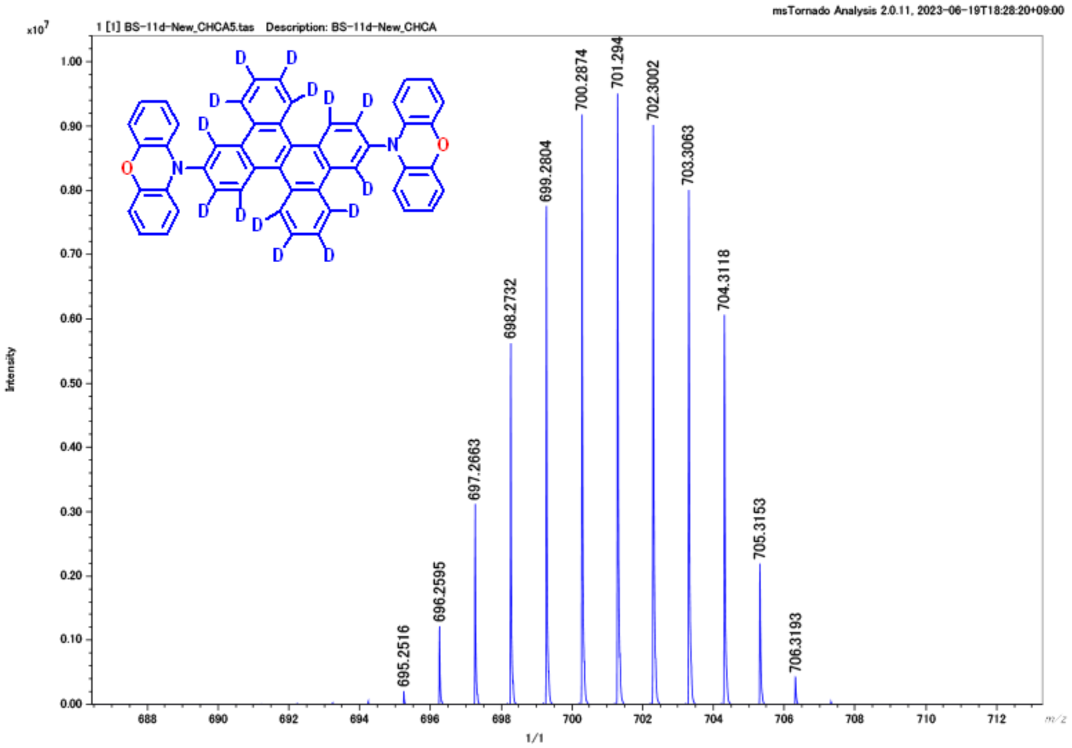

Figure S13. HRMS–MALDI mass spectrum of 2,10-di(10*H*-phenoxazin-10-yl)dibenzo[*g*,*p*]chrysene-d_{14} (**3d**).

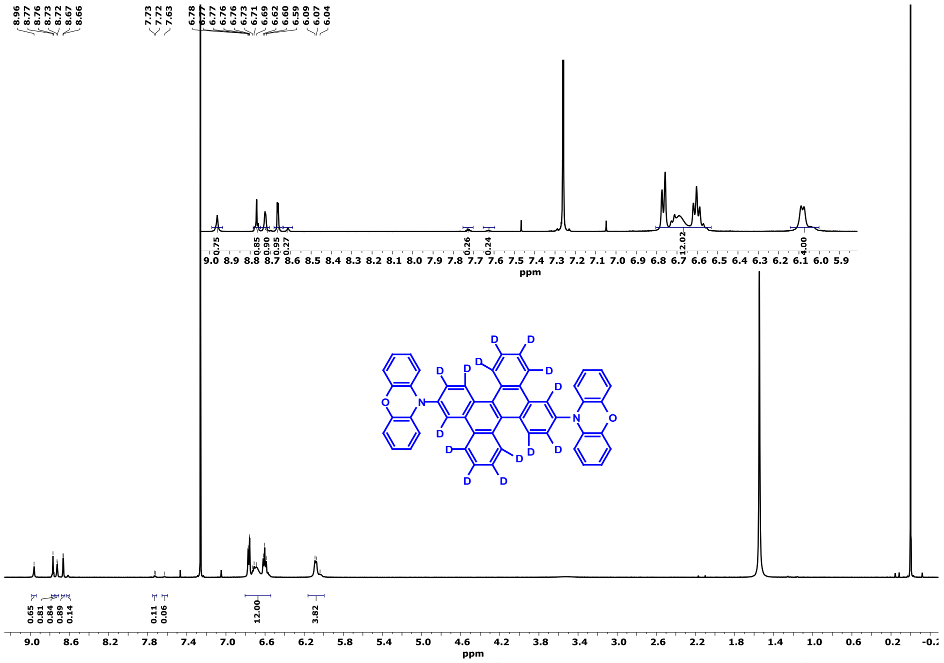

Figure S14. Calculation of deuteration yield by ^{1}H NMR spectroscopy. ^{1}H NMR spectrum of 10,10'-(dibenzo[*g*,*p*]chrysene-2,10-diyl-d14)bis(10*H*-phenoxazine) (**3d**) in $CDCl_3$. The phenoxazine protons were chosen as the reference for calculating the deuteration yield of the DBC core and found 75%.

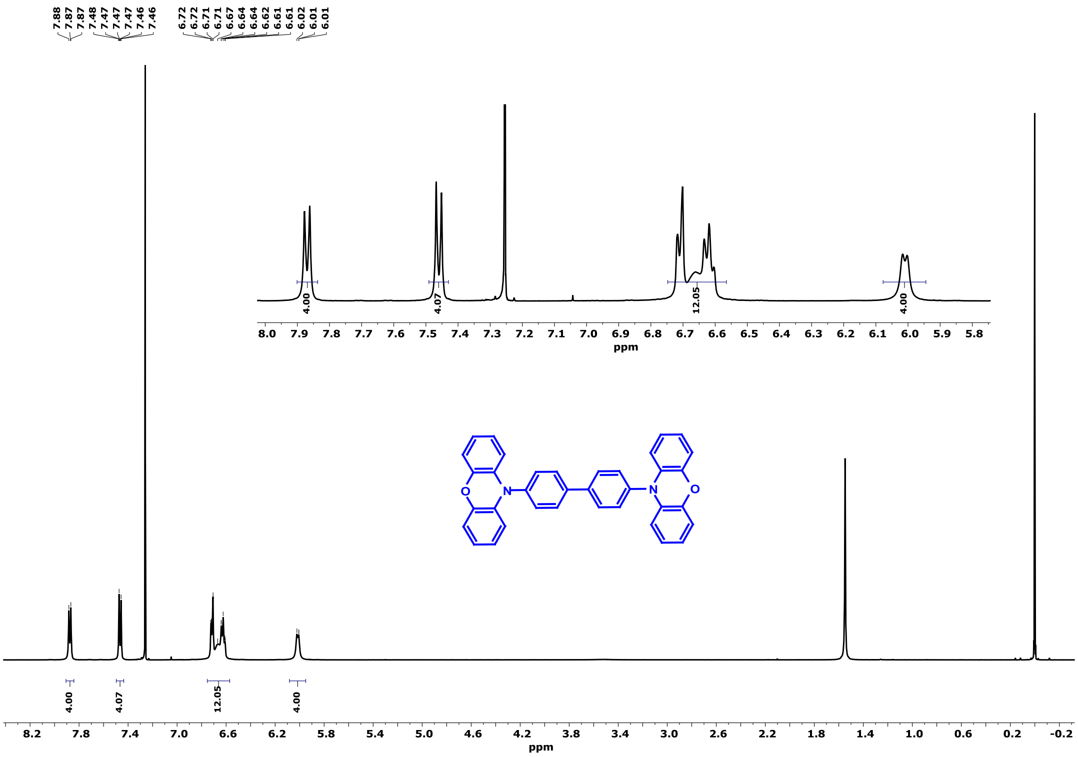

Figure S15. ^{1}H NMR spectrum of 4,4'-di(10*H*-phenoxazin-10-yl)-1,1'-biphenyl (**4h**) in $CDCl_3$.

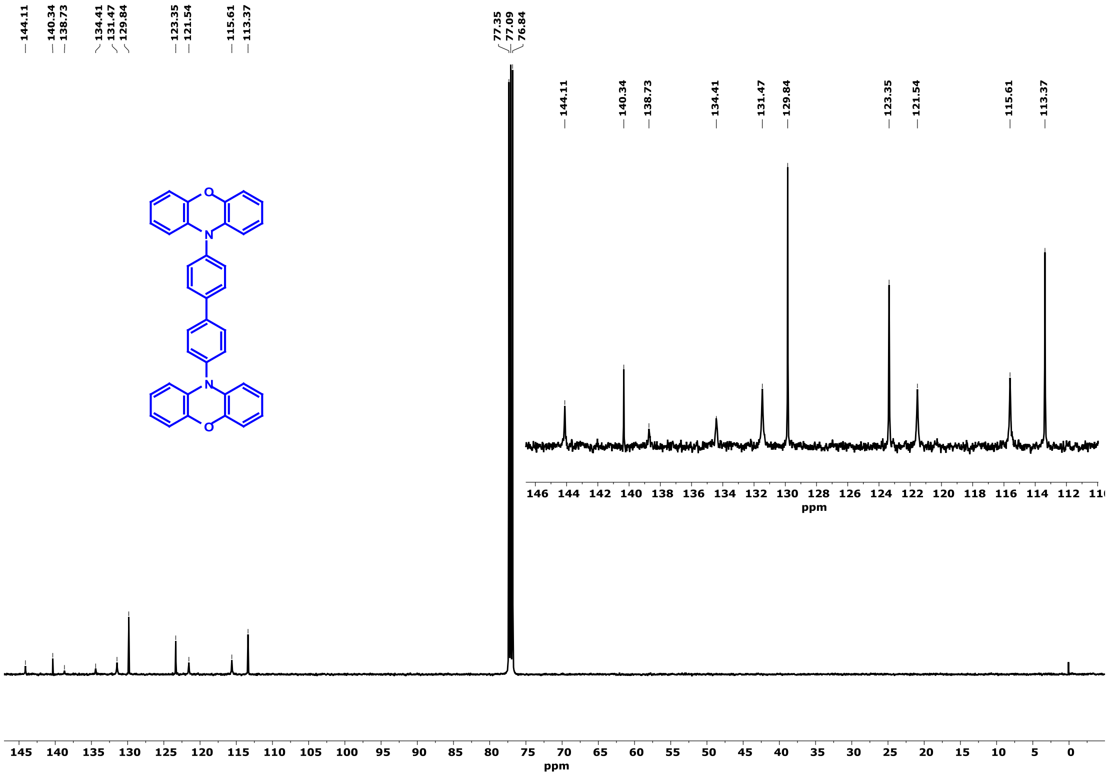

Figure S16. ^{13}C NMR spectrum of 4,4'-di(10*H*-phenoxazin-10-yl)-1,1'-biphenyl (**4h**) in $CDCl_3$.

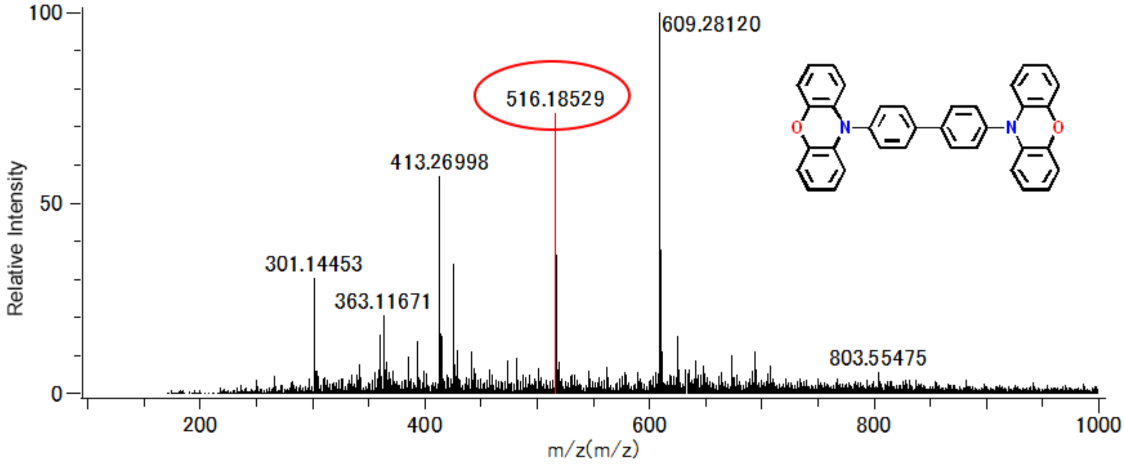

Figure S17. HRMS–ESI mass spectrum of 4,4'-di(10*H*-phenoxazin-10-yl)-1,1'-biphenyl (**4h**).

Section S4. Optical measurements

The absorption spectra were measured by using a absorption photo-chrometer (V-670, JASCO, Tokyo, Japan). The steady-state RT emission yield under vacuum (Φ_e(RT)) was determined by using absolute photoluminescence quantum yield equipment (C9920-02G, Hamamatsu Photonics, Shizuoka, Japan) (Figure S19a). In the measurement, emission signal (red line in Figure 2a) was determined by integrating photon signals in each wavelength from the sample before, under, and after ceasing excitation (Figure S19b). To compare steady-state emission intensity under excitation with afterglow emission intensity soon after ceasing excitation, the time change of emission spectral intensity before, under, and after ceasing excitation was measured by using monochromatic light from the excitation unit of a fluorimeter (FP-8300, JASCO) as an excitation and a photonic multichannel analyzer (C10027-01, Hamamatsu Photonics) as a photodetector. The emission spectral intensity of steady-state emission under excitation at 340 nm (A_1) was compared with the emission spectral intensity of afterglow emission spectra soon (20–40 ms) after cessation of the 360-nm excitation (A_2) [Figure S19c]. Φ_p(RT) was determined from $\Phi_p(RT) = \Phi_e(RT)A_2/A_1$. The fluorescence yield at RT (Φ_f(RT)) was determined as $\Phi_e(RT) - \Phi_p(RT)$. Reference S3 summarizes the logical reasonability of the determination procedure of Φ_p(RT). The temperature dependence of fluorescence and phosphorescence characteristics as per a cryostat (Optistat DN-V, Oxford Instruments, Abingdon-on-Thames, UK) was used to change the temperature. The fluorescence intensity change and phosphorescence intensity change were recorded by changing the temperature. Φ_f(T) and Φ_p(T) were determined by comparing the emission intensities at T K with those at RT. The yield of intersystem crossing from S_1 to triplet states (Φ_{isc}) was determined by using transient absorption techniques. Transient absorption measurements were performed with a sub-nanosecond transient absorption spectrophotometer (picoTAS, Unisok, Osaka, Japan) equipped with a 355-nm, Q-switched microchip laser (PNV-M02510-1×0, Teem Photonics, Meylan, France). Section S6 provides a more detailed procedure.

Section S5. Optical properties in amorphous *β*-estradiol host

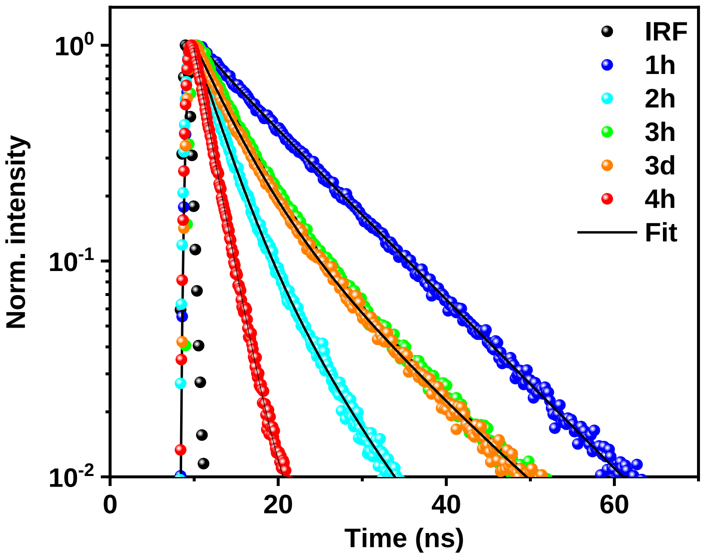

Figure S18. Normalized fluorescence decay profile of chromophores **1h**–**3h**, **3d**, and **4h** in amorphous *β*-estradiol (0.3 wt%) at room temperature.

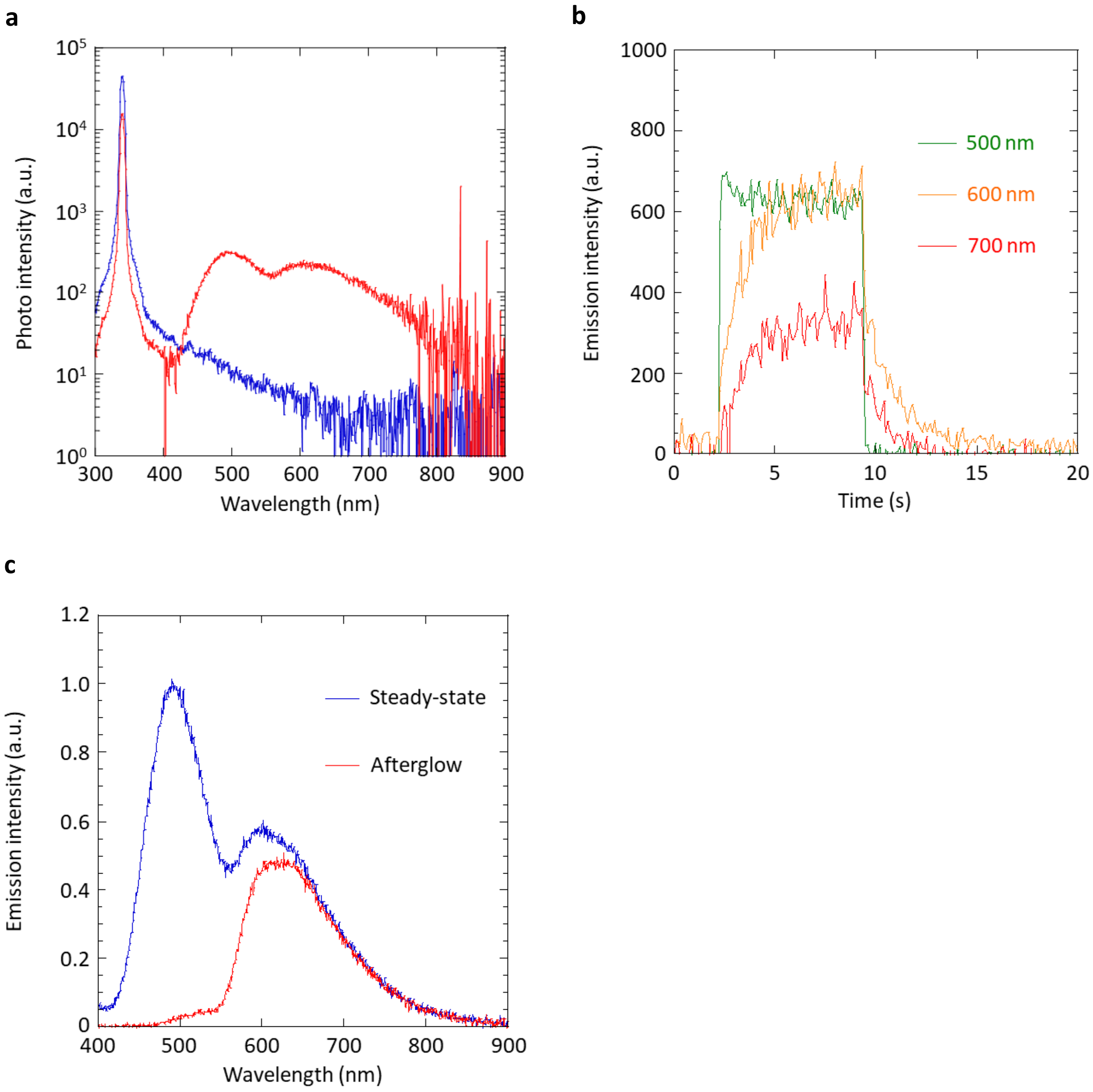

Figure S19. Information for determining the RTP yield of 0.3 wt% **3d** doped into an amorphous *β*-estradiol film on a quartz substrate as a sample. (a) Signals from the sample (red line) and a quartz substrate as a reference (blue line) in the integration sphere. Excitation: 340 nm. (b) Time change of photons from samples at 500, 600, and 700 nm when excitation at 340 nm started at 2.4 s and stopped 9.4 s in the integration sphere. (c) Steady-state emission spectral intensity under 340-nm excitation and afterglow emission spectral intensity at RT. In (a)–(c), measurements were performed under vacuum.

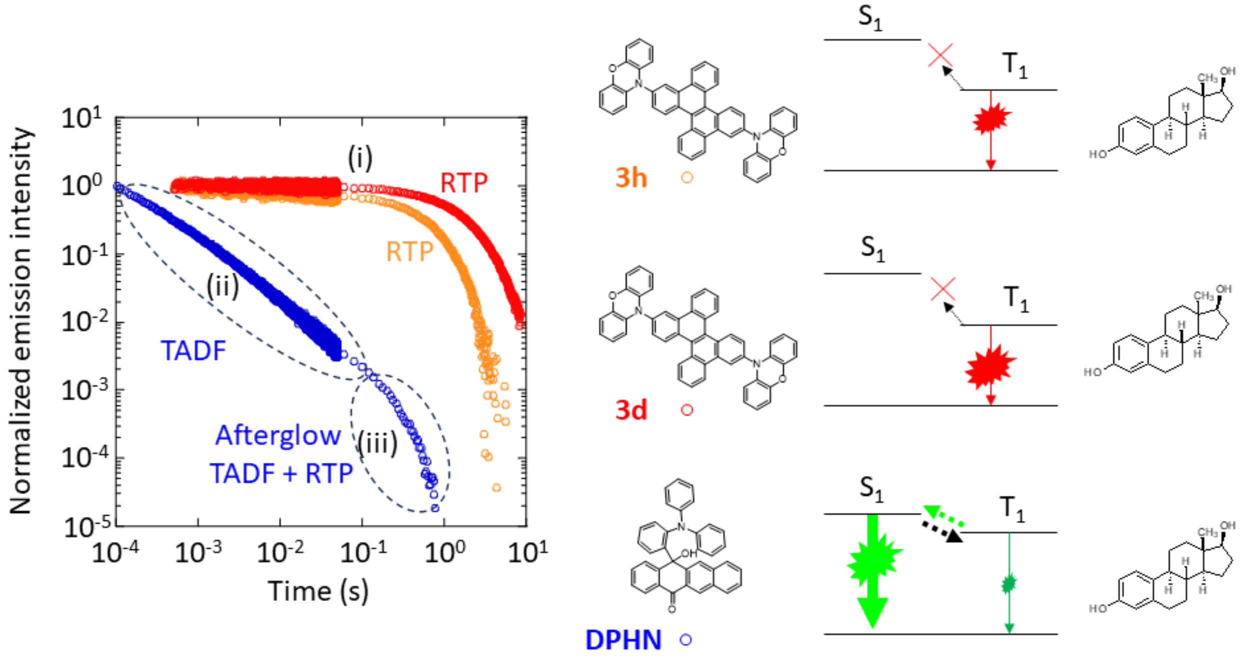

Figure S20. Afterglow emission decay characteristics of chromophores **3h**, **3d**, and **DPHN** dispersed in amorphous *β*-estradiol (0.3 wt%) at room temperature (log emission intensity vs log time plots). Chromophore **DPHN** has been reported in reference [S6]. Chromophores **3h** and **3d** were used as examples of a molecule to indicate afterglow emission with RTP character in an amorphous *β*-estradiol host. Chromophore **DPHN** was used as an example of a molecule to indicate afterglow emission with thermally activated delayed fluorescence (TADF) and RTP characteristics in an amorphous *β*-estradiol host. Regarding afterglow emitting materials with pure RTP character and an average afterglow lifetime approaching 1 s, the emission intensity after ceasing excitation negligibly decreased for 100 ms after cessation of the excitation (i). Regarding afterglow emitting materials with TADF and RTP characteristics, the emission intensity after ceasing excitation substantially decreased for 100 ms after cessation of the excitation (ii) even there was a long afterglow component caused by a long-lived triplet state (iii). Regarding afterglow emitting materials with pure RTP character, the afterglow components were collected with cost-effective two-dimensional photodetectors such as a charge-coupled device (CCD) and a complementary metal-oxide semiconductor (CMOS). This is because most photons were generated within 0.1–1 s, which can be collected by the two-dimensional photodetectors. However, most of photons were generated before 100 ms for afterglow emitting materials with TADF and RTP characteristics because TADF acts as a leakage of afterglow. Therefore, collection of photons after 100 ms was insubstantial.

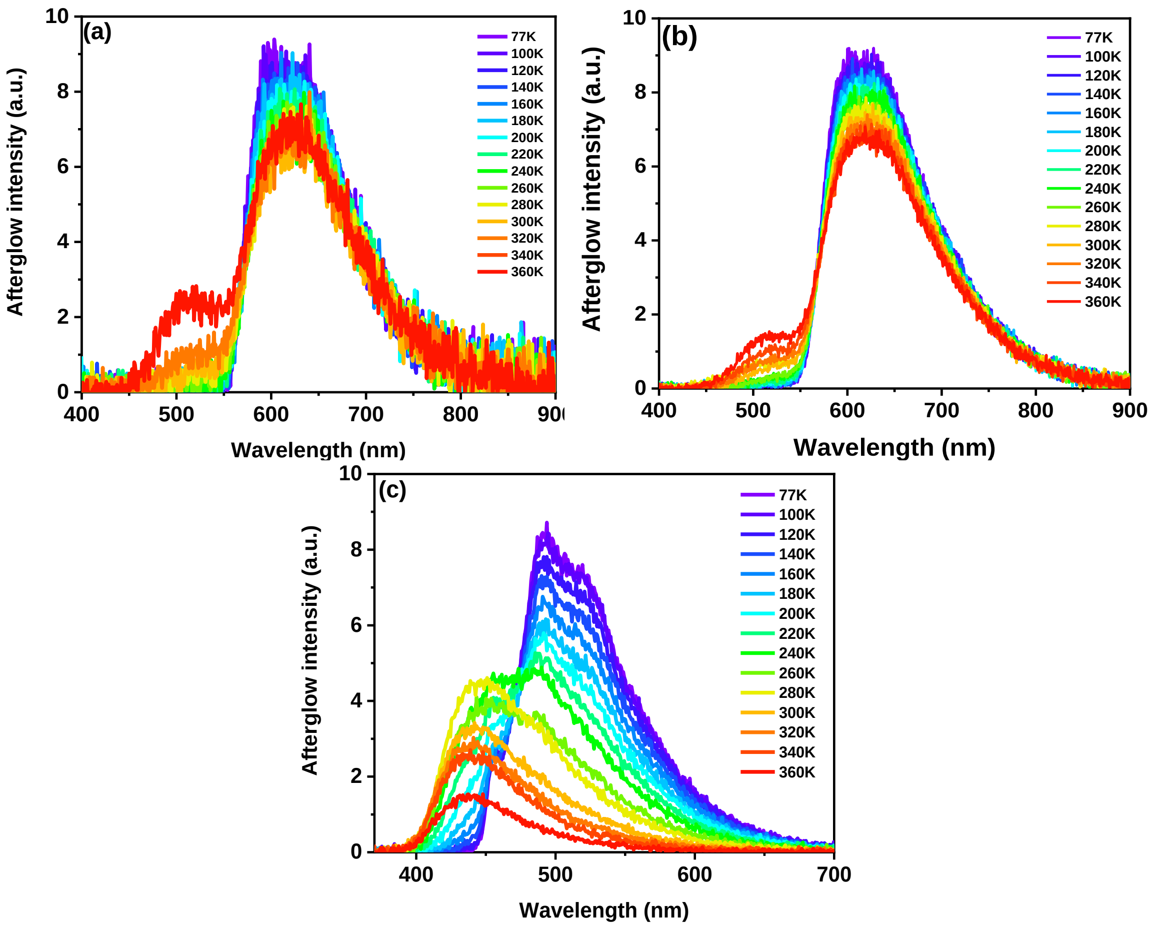

Figure S21. Temperature dependence of persistent phosphorescence spectral intensities in chromophores (a) **3h**, (b) **3d**, and (c) **4h** in amorphous *β*-estradiol. The concentration of chromophores was 0.3 wt% and the excitation wavelength was 340 nm.

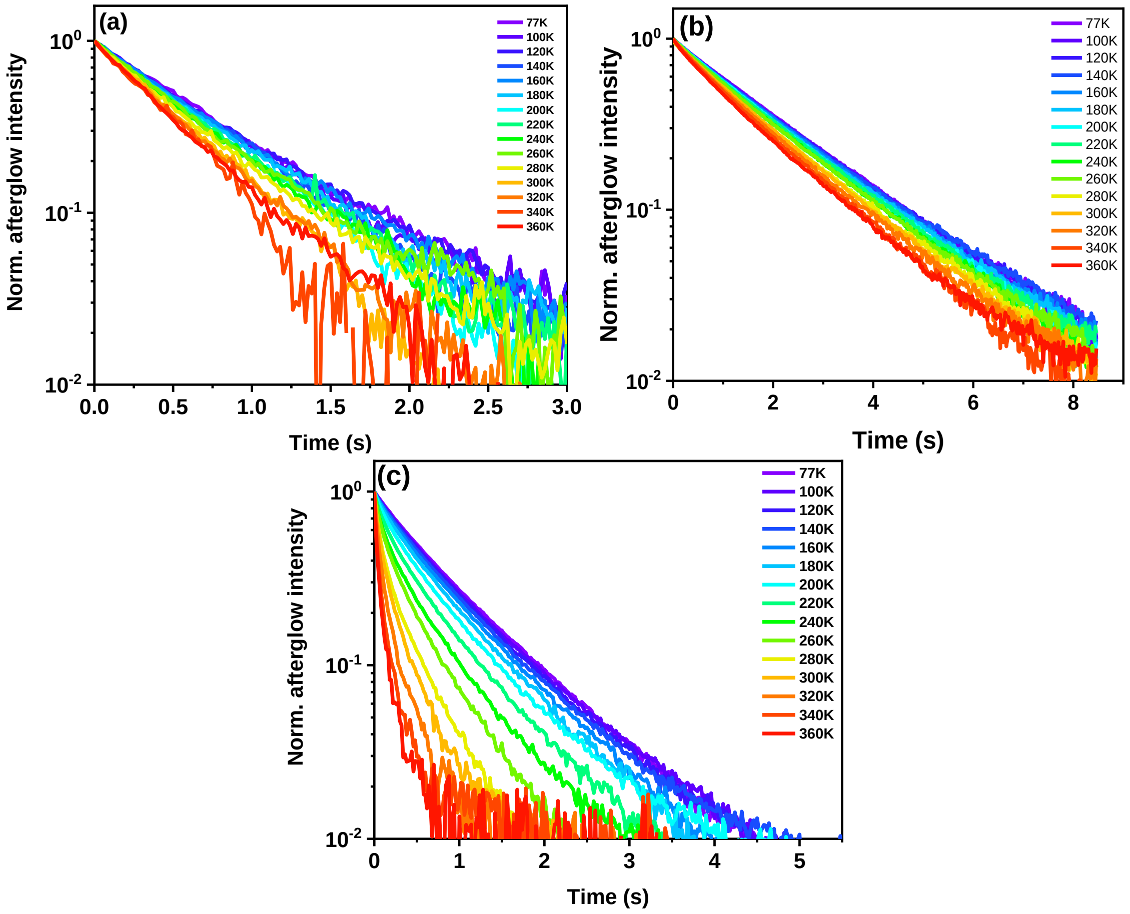

Figure S22. Temperature dependence of persistent phosphorescence decay in chromophores (a) **3h**, (b) **3d**, and (c) **4h** in amorphous *β*-estradiol. The concentration of the chromophores was 0.3 wt% and the excitation wavelength was 340 nm.

Section S6. Determination of Φ_{isc}

The intersystem crossing (ISC) yield from the low-lying singlet excited state (S_1) to triplet states (Φ_{isc}) at RT was determined experimentally via triplet–triplet (T–T) energy transfer from a triplet sensitizer donor to a β-carotene acceptor.[S3,S7,S8] Benzophenone was used as a reference with $\Phi_{isc} = 1$ for evaluating Φ_{isc} for **3h**, **3d**, and **4h** in benzene. The optical density of the **3h**, **3d**, and **4h** samples, as well as the benzophenone reference, was fixed at OD = 1 at 355-nm excitation, and 0.5 mM β-carotene was mixed in benzene in a 1-mm-thick quartz cell. The samples and reference were degassed thoroughly 3× by freeze–pump–thaw cycles before the transient absorptions (355-nm excitation, 530-nm detection) were measured. Transient absorption decay kinetics of the samples and the reference in the absence of β-carotene (OD = 1 at 355 nm) were also recorded by using the same optical setup.

All transient absorption kinetics and spectra were measured with a sub-nanosecond transient absorption spectrophotometer (picoTAS, Unisoku, Osaka, Japan) and a 355-nm Q-switched microchip laser (PNV–M02510–1×0, Teem Photonics, Meylan, France). A, B, τ_1, and τ_2 were determined by fitting data in Figure S24 by using Equation (S1):

$$\Delta A = A\left(1 - e^{-t/\tau_1}\right)e^{-t/\tau_2} + Be^{-t/\tau_1} \quad , \qquad \text{(S1)}$$

where t is the time after excitation with a 355-nm pulse. The energy transfer efficiency from the low-lying excited triplet state (T_1) of the triplet sensitizer to T_1 of β-carotene (Φ_{T-T}) in the benzene solution was calculated by using:

$$\Phi_{T-T} = \frac{\tau_0 - \tau_1}{\tau_0}, \qquad \text{(S2)}$$

where τ_0 and τ_1 are the sensitizer triplet lifetimes in the absence and presence of β-carotene, respectively. Transient absorption decay kinetics in the absence of the triplet β-carotene acceptor were fitted with Equations (S3 and S4) to evaluate the average triplet lifetimes (τ_0) of the samples and reference:

$$\text{For single exponential, } F(t) = Ae^{-t/\tau} \qquad \text{(S3)}$$

$$\text{Multiexponential, } F(t) = A_1e^{-t/\tau_1} + A_2e^{-t/\tau_2}. \qquad \text{(S4)}$$

Table S2 and Figure S25 summarize the τ_0 of **3h**, **3d**, **4h**, and the reference in the absence of β-carotene. Figure S24 shows the changes in transient absorption decays that are attributable to T–T energy transfer from the triplet sensitizer to β-carotene. Φ_{isc} at RT for **3h**, **3d**, and **4h** in benzene were calculated by using:

$$\Phi_{isc} = \frac{\alpha A}{\Phi_{T-T}}, \qquad \text{(S5)}$$

where α is a constant if the optical setup and the 355-nm excitation power remain unchanged. The value of α was determined when Φ_{isc} of the reference sample (benzophenone) at RT was set to 1. Accordingly, Φ_{isc} for **3h**, **3d**, and **4h** were determined (Table S2). Reference [S9] shows data regarding Φ_{isc} of **1h** and **2h**.

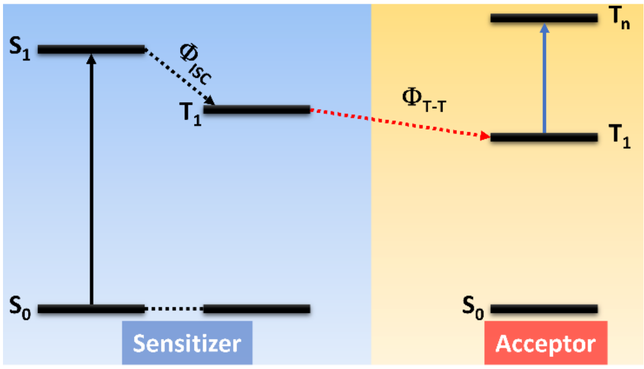

Scheme S23. Schematic of triplet–triplet energy transfer (Φ_{T-T}) from a triplet sensitizer to an acceptor for Φ_{isc} determination of the triplet sensitizer at room temperature.

Table S2. Parameters used to determine Φ_{isc} for **3h**, **3d**, and **4h** in degassed benzene at room temperature. Benzophenone with $\Phi_{isc} = 1$ was used as the reference and *β*-carotene was used as the triplet energy acceptor. The concentration of *β*-carotene was 0.5 mM and the optical density of all the solutions was set to OD = 1. The experiment error range for Φ_{isc} was ±25%.

Sample	A	B	τ_1 (μs)	τ_2 (μs)	τ_0 (μs)	$\Phi_{T\text{-}T}$	Φ_{isc}
**Benzophenone**	0.0202	0.0045	205	6067	6159	0.97	1
**3h**	0.0178	0.0010	834	5570	191860	0.99	0.85 (±0.21)
**3d**	0.0185	0.0008	1150	5546	214452	0.99	0.88 (±0.22)
**Benzophenone**	0.0296	0.0065	219	6022	5134	0.96	1
**4h**	0.0269	0.0041	754	5877	12626	0.94	0.92 (±0.22)

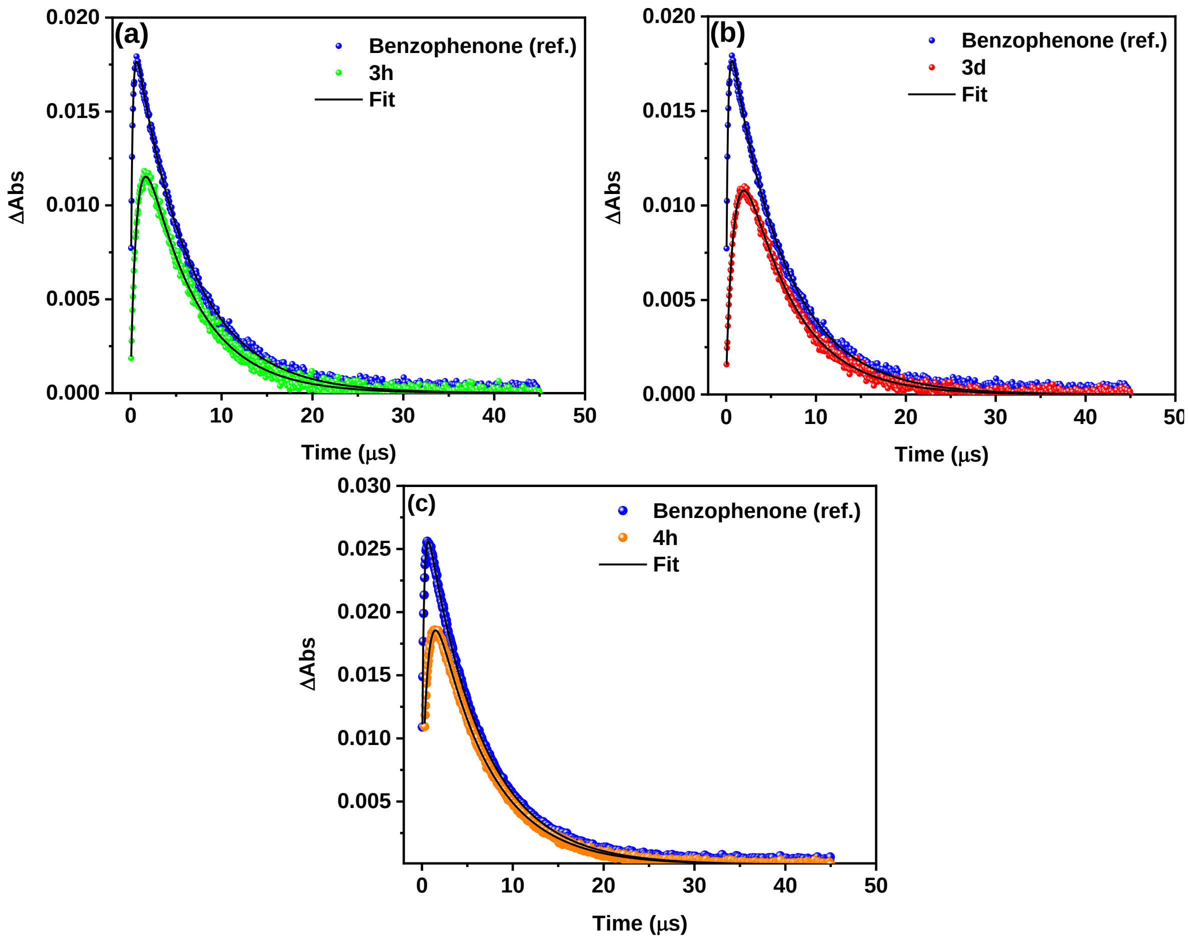

Figure S24. Triplet–triplet absorption intensity changes at 532 nm for **3h**, **3d**, and **4h** sample solutions, as well as benzophenone reference solution. The sample solutions contained **3h**, **3d**, and **4h** as a triplet sensitizer, as well as *β*-carotene as an acceptor in degassed benzene. The reference solution had benzophenone as a triplet sensitizer and *β*-carotene as an acceptor in degassed benzene.

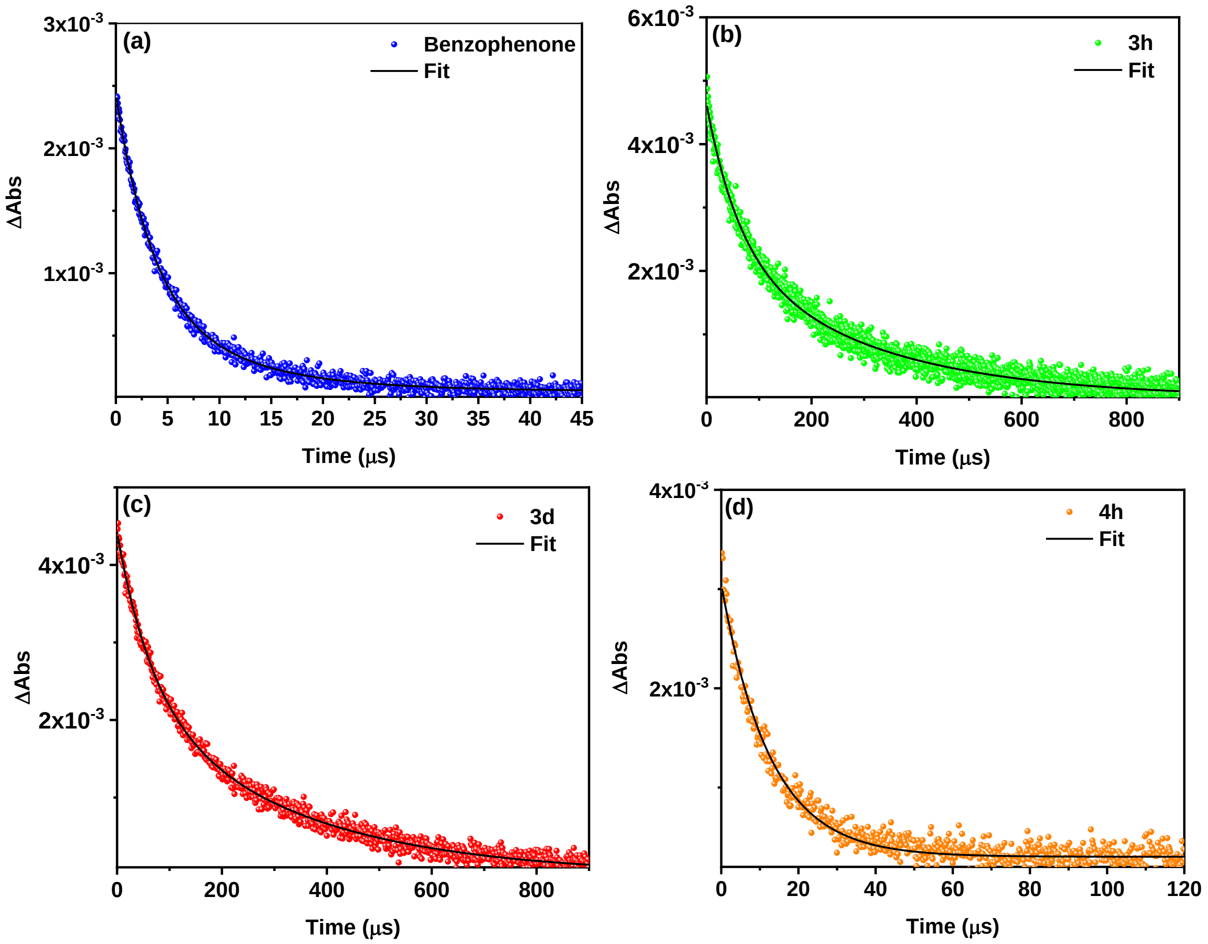

Figure S25. Triplet–triplet absorption decay characteristic of (a) benzophenone (532-nm excitation), (b) **3h** (460-nm excitation), (c) **3d** (460-nm excitation), and **4h** (455-nm excitation) in degassed benzene at room temperature.

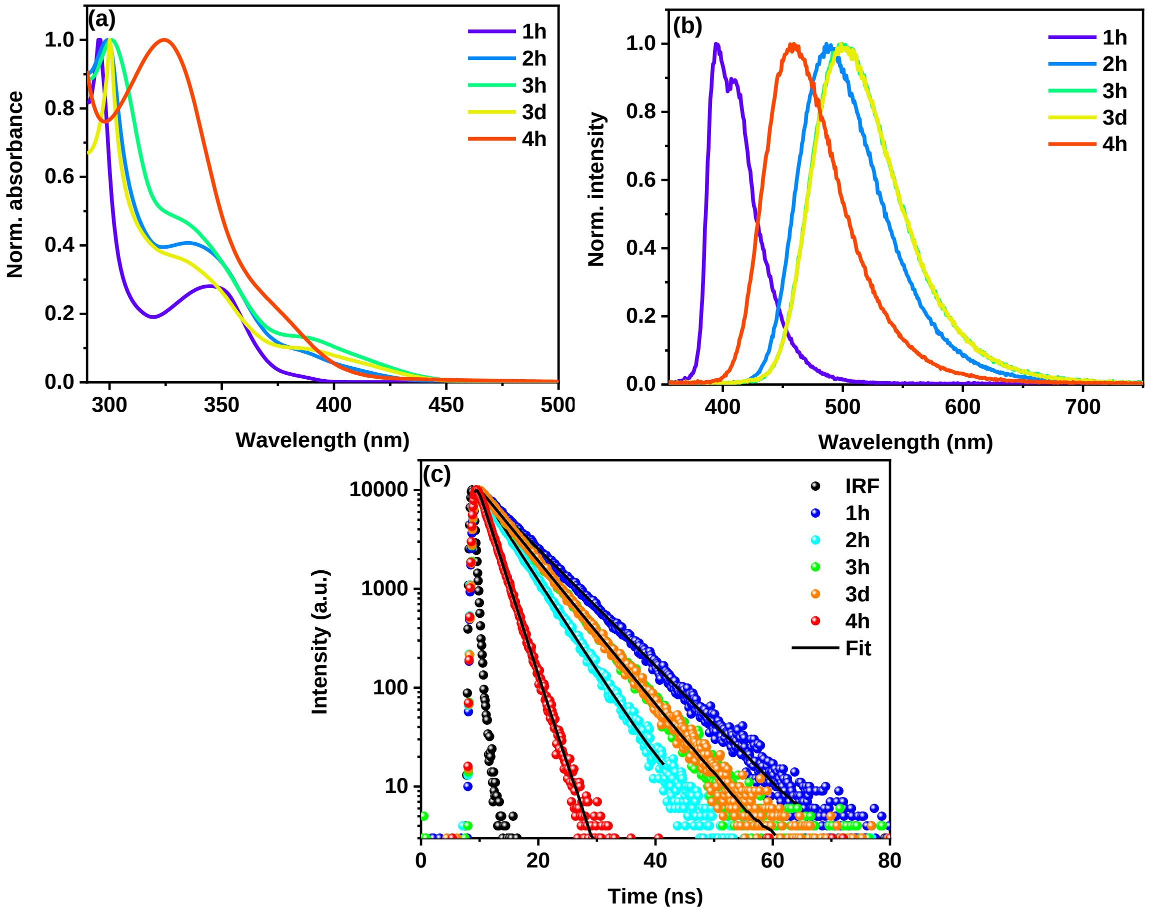

Figure S26. Normalized (a) absorption spectra, (b) fluorescence spectra, and (c) emission decay characteristics of chromophores **1h**–**3h**, **3d**, and **4h** in benzene (10^{-5} M) at room temperature.

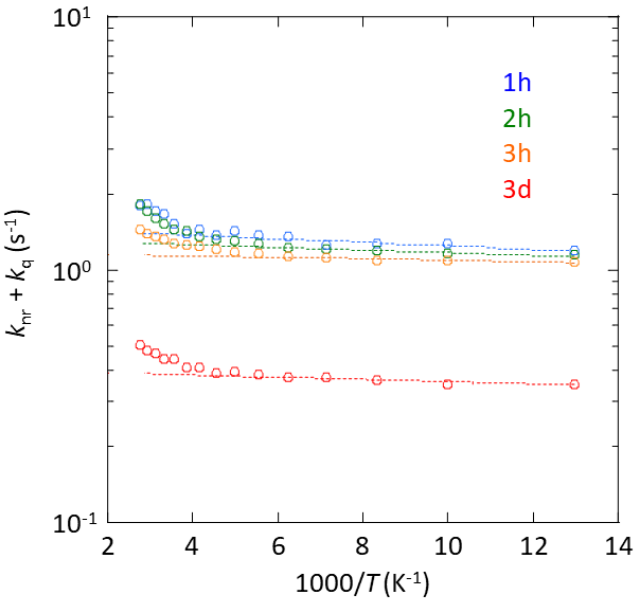

Figure S27. Temperature dependence of $k_{nr} + k_q$ of 0.3 wt% chromophore-doped amorphous β-estradiol. Dotted lines are fitting lines of k_{nr} when k_{nr} has an exponential function.

Table S3. Photophysical of chromophores **1h**–**3h**, **3d**, and **4h** in benzene (10^{-5} M) at room temperature.

Sample	λ_f (nm)^a	Φ_f	τ_f (ns)	k_f (10^9 s^{-1})^b	Φ_{isc}^{c}	k_{isc} (10^9 s^{-1})^d
**1h**	395, 410	0.16	7.3	0.022	0.84	0.11
**2h**	490	0.16	4.7	0.033	0.84	0.18
**3h**	500	0.19	6.0	0.031	0.81	0.14
**3d**	500	0.19	6.0	0.031	0.81	0.14
**4h**	460	0.11	2.3	0.047	0.89	0.38

^{a} The peak wavelengths of fluorescence. ^{b} $k_f = \Phi_f/\tau_f$, ^{c} Φ_{isc}(RT) = 1 − Φ_f(RT). ^{d} $k_{isc} = \Phi_{isc}/\tau_f$.

Section S7. Optical properties in polymer hosts

Polymethyl methacrylate (PMMA) (average M_w = 350,000, Sigma–Aldrich, St. Louis, Missouri, USA), Zeonex (Zeonex480, Zeon, Tokyo, Japan), and polystyrene (average M_w = 280,000, Sigma–Aldrich) polymers were dissolved in toluene, chloroform, and toluene, respectively, at a concentration of 50 mg/mL. Then, guest chromophore powders with a concentration of 0.3 wt% were mixed with the polymer solution. Thin films were drop-casted onto a quartz substrate and annealed at 100°C for 10 min. Next, a thin layer of polyvinyl alcohol polymers was coated on top of each film to suppress oxygen penetration. The oxygen consumption using 365 nm UV irradiation was performed for 5–10 min prior to photophysical experiments.

Table S4. Summary of photophysical parameters of 0.3 wt% chromophore **3d** in various host matrices at room temperature.

Host	λ_f[a] (nm)	Φ_f(RT)	τ_f(RT) (ns)	k_f[b] (10^7 s^{-1})	Φ_{isc}[c]	k_{isc}[d] (10^7 s^{-1})
β-estradiol	491	0.28	6.8	4.0	0.72	11
Zeonex	460	0.065	3.4	1.9	0.94	28
PMMA	478	0.15	6.2	2.4	0.85	14
Polystyrene	471	0.14	4.7	2.9	0.86	18

a) Peak wavelength of fluorescence. b) Value determined with $k_f = \Phi_f(RT)/\tau_f(RT)$. c) Value determined based on $\Phi_{isc} = 1 - \Phi_f(RT)$. d) Value determined with $k_{isc} = \Phi_{isc}/\tau_f(RT)$.

Table S5. Summary of radiative and nonradiative decay rates from triplet state of 0.3 wt% chromophore **3d** in various host matrices at room temperature.

Host	Φ_p(RT)	τ_p(RT)[a] (s)	λ_p[b] (nm)	k_p[c] (s^{-1})	k_{nr}[d] (s^{-1})	k_q[e] (s^{-1})
β-estradiol	0.21	1.63	625	0.180	0.39	0.061
Zeonex	0.05	0.84	630	0.065	0.75	0.064
PMMA	0.14	1.16	625	0.142	0.56	0.22
Polystyrene	0.13	1.12	630	0.139	0.53	0.19

a) Single-exponential fitting data. b) Peak wavelength of phosphorescence. c) Values determined by substituting experimentally observed Φ_p(RT), τ_p(RT), and $\Phi_{isc} = 1 - \Phi_f$ (RT) into $k_p = \Phi_p(RT)/(\Phi_{isc}\tau_p)$. d) Values determined by using fitting lines of $k_{nr}(T)$ in Figure S28. g) Values determined by subtracting k_{nr}(RT), determined by using the fitting lines of $k_{nr}(T)$ in Figure S28, from experimentally observed $k_{nr}(T) + k_q(T)$.

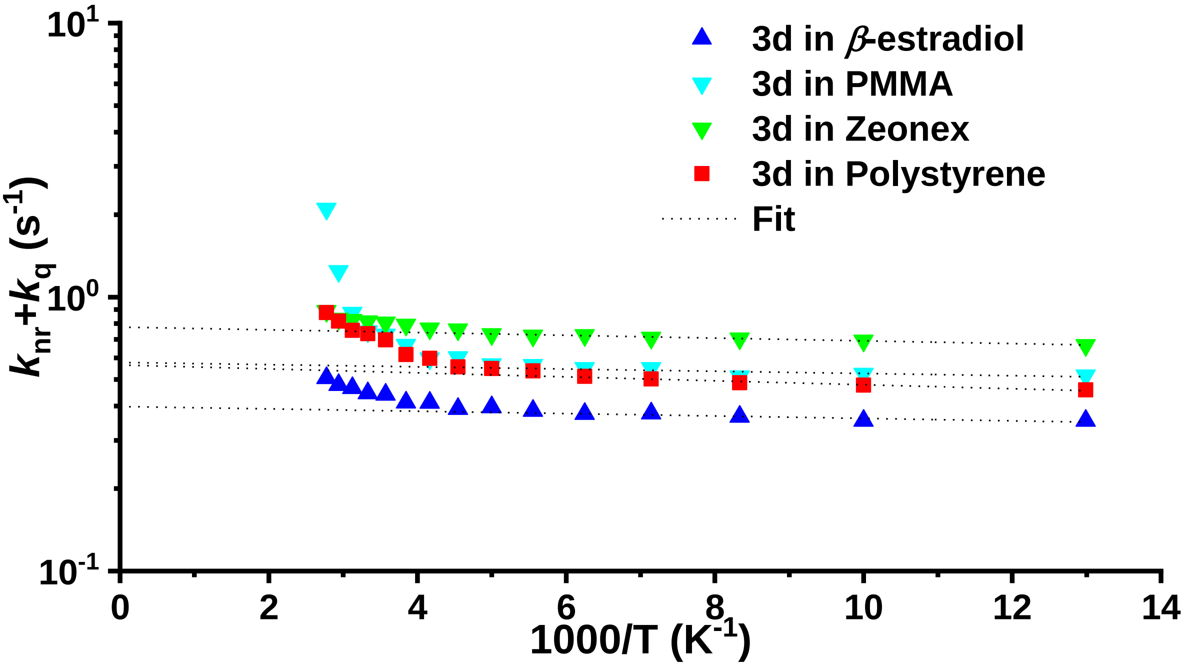

Figure S28. Temperature dependence of k_{nr} + k_q of 0.3 wt% **3d** chromophore-doped in amorphous *β*-estradiol, PMMA, Zeonex, and polystyrene host. Dotted lines are fitting lines of k_{nr} when k_{nr} has an exponential function.

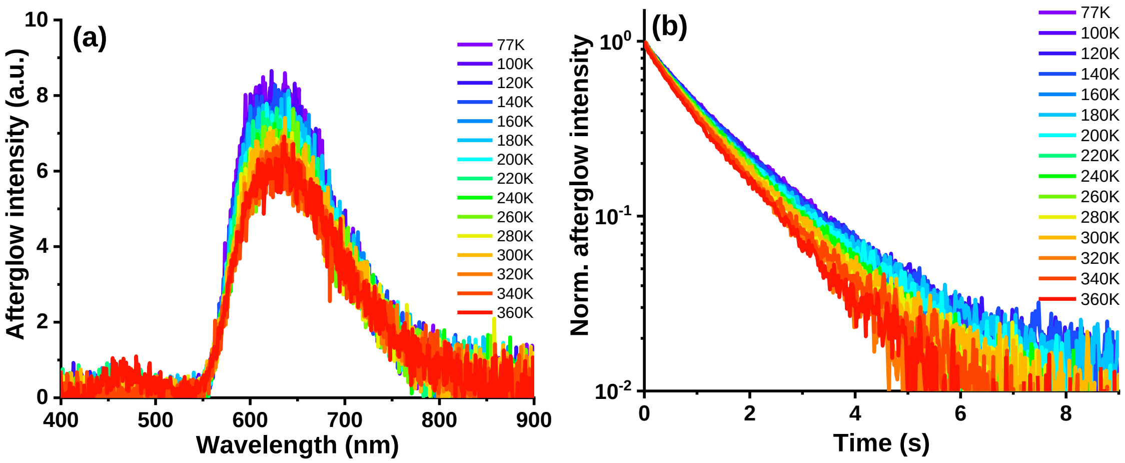

Figure S29. Temperature dependence of persistent phosphorescence (a) spectral intensities and (b) decay profile of chromophore **3d** in Zeonex polymer matrix. The concentration of chromophores was 0.3 wt%, and the excitation wavelength was 340 nm.

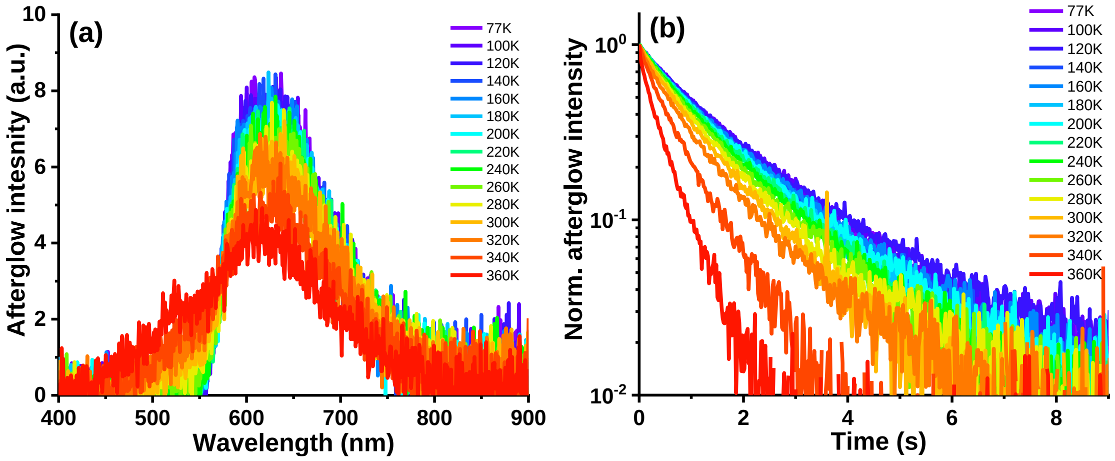

Figure S30. Temperature dependence of persistent phosphorescence (a) spectral intensities and (b) decay profile of chromophore **3d** in PMMA polymer matrix. The concentration of chromophores was 0.3 wt%, and the excitation wavelength was 340 nm.

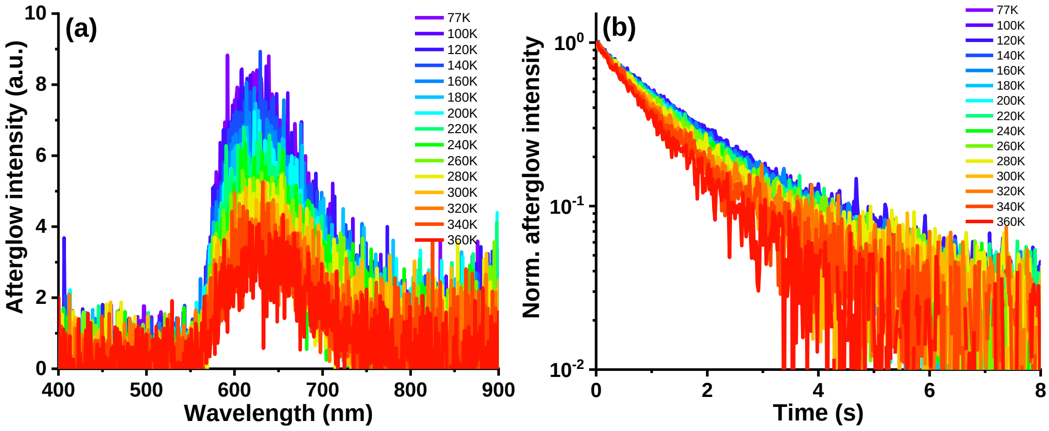

Figure S31. Temperature dependence of persistent phosphorescence (a) spectral intensities and (b) decay profile of chromophore **3d** in polystyrene polymer matrix. The concentration of chromophores was 0.3 wt% and the excitation wavelength was 340 nm.

Section S8. Effect of k_{nr} on Change of Dihedral Angle of D-π-D Structure

Because the enhancement of k_p by using the heavy-atom effect is common and also increases k_{nr}(RT),[S10,S11] we considered the contribution of the symmetry-breaking caused by the independent changes of θ_1 and θ_2 to k_{nr}(RT). In principle, k_{nr}(RT) is proportional to the square of the SOC between T_1 and S_0 ($\langle S_0|\hat{H}_{SO}|T_1\rangle$) and the Franck–Condon factor between T_1 and S_0 (FC).[S12] Since the energy gap law is contained in FC and FC is approximately inversely proportional to the square of the energy difference between T_1 and S_0 ($E_{T_1-S_0}$) when $E_{T_1-S_0}$ is a visible wavelength,[S12,S13] k_{nr}(RT) is approximately proportional to $\langle S_0|\hat{H}_{SO}|T_1\rangle^2/E_{T_1-S_0}{}^2$. The $E_{T_1-S_0}$ of **3h** changed by <±5% of the values upon independently changing θ_1 and θ_2 from 40° to 140° (Figure S37). The changes of $\langle S_0|\hat{H}_{SO}|T_1\rangle^2$ were <±12% of the values upon independently changing θ_1 and θ_2 from 40° to 140° (Figure S37). Because the possibility of geometries with θ_1 and θ_2 <50° and >100° is small at RT, the changes of $\langle S_0|\hat{H}_{SO}|T_1\rangle^2$ were $\ll$ ±12%. Insignificant changes of $E_{T_1-S_0}{}^2$ [Figure S37c (i)] and $\langle S_0|\hat{H}_{SO}|T_1\rangle^2$ [Figure S37c (ii)] over a variety of θ_1 and θ_2 explain no substantial increase of k_{nr}(RT). Therefore, the symmetry-breaking because of independently changing the θ_1 and θ_2 of **3h** selectively enhanced k_p without a large increase of k_{nr}(RT). Although the rigidity of the chemical structure and chemical interactions that induce rigidity has often been phenomenologically explained regarding enhanced emission characteristics, the demonstrated good correlation between optically determined values and calculated values for k_p and k_{nr}(RT) based on dynamic calculations indicates that flexibility of some conjugated molecules corresponds to selectively enhancing k_p compared with k_{nr}(RT). Calculations without considering such flexibility of θ_1 and θ_2 underestimated k_p of **3h**, which was a bottleneck to discovering state-of-the-art persistent RTP chromophores at long wavelengths.

Section S9. Quantum chemical calculations

A geometry of the lowest triplet excited state (T_1) was optimized by using density functional theory (DFT) based on Gaussian09 with the B3LYP functional and 6-31G(d) basis set. Vibrational information of T_1 geometry was calculated by DFT based on Gaussian09 with the B3LYP functional and 6-31G(d) basis set.

To determine vibrational spin-orbit coupling (SOC) between T_1 and S_0 (VSOC) at room temperature, the SOC between T_1 and S_0 ($\langle S_0|\hat{H}_{SO}|T_1\rangle$), depending on the mass-weighted normal coordinates at T_1 (Q_p) [$\langle T_1|\hat{H}_{SO}|S_0\rangle_{Q_p}$], was calculated by using the Amsterdam Density Functional (ADF) 2018 package at Q_p=−1, −0.8, −0.6, −0.4, −0.2, 0, +0.2, +0.4, +0.6, +0.8, and +1.0. The SOC operator within the zeroth-order regular approximation was $\hat{H}_{SO}$. The parameter $\langle S_0|\hat{H}_{SO}|T_1\rangle$ was treated as a perturbation based on the scalar relativistic orbitals with the PBE0 functional and TZP basis set. The relationship between Q_p and $\langle T_1|\hat{H}_{SO}|S_0\rangle_{Q_p}{}^2$ [Figure S32a, (i)], where $\langle T_1|\hat{H}_{SO}|S_0\rangle_{Q_p}{}^2$ represents $\langle T_1|\hat{H}_{SO}|S_0\rangle^2$ at Q_p, was used to obtain the relationship between $|\partial\langle T_1|\hat{H}_{SO}|S_0\rangle_{Q_p}/\partial Q_p|^2$ and Q_p [Figure S32a, (ii)]. The increase of the T_1 energy at Q_p geometry compared with that of the optimized T_1 geometry (ΔE_{Q_p}) was determined by DFT based on Gaussian09 with the B3LYP functional and 6-31G(d) basis set. ΔE_{Q_p} was also plotted vs. Q_p [Figure S32a, (iii)]. The possibility of the coordinate of Q_p [P_{Q_p}(RT)] can be determined by using $P_{Q_p}(RT) = \exp(-\Delta E_{Q_p}/kT)$ based on the Boltzmann distribution [Figure S32a, (iv)], where k is the Boltzmann constant and the possibility of an optimized T_1 geometry is defined as unity. Integrating $\left|\partial\langle T_1|\hat{H}_{SO}|S_0\rangle_{Q_p}/\partial Q_p\right|^2 P_{Q_p}(\mathrm{RT})$ for Q_p gives $\left|\partial\langle T_1|\hat{H}_{SO}|S_0\rangle/\partial Q_p\right|^2 P(\mathrm{RT})$ [Figure S32a, (v)]. Table S6 shows information regarding parameters to determine $\left|\partial\langle T_1|\hat{H}_{SO}|S_0\rangle/\partial Q_p\right|^2 P(\mathrm{RT})$ of **3h**. The information in the tables was used in Figure S32b for determining $\sum_p\left|\partial\langle T_1|\hat{H}_{SO}|S_0\rangle/\partial Q_p\right|^2 P(\mathrm{RT})$. Calculations regarding **1h** and **2h** were performed by using the same procedure and have been reported in reference S9. The Frank–Condon factor between T_1 and S_0 (FC) was approximated by using a previously reported procedure in References S12 and S13 (Table S7 and Figure S33). The relationship between $\sum_p\left|\partial\langle T_1|\hat{H}_{SO}|S_0\rangle/\partial Q_p\right|^2 P(\mathrm{RT})$ and the optically determined k_{nr}(RT) of **1h**–**3h** was set on the statistical data regarding the relationship between $\sum_p\left|\partial\langle T_1|\hat{H}_{SO}|S_0\rangle/\partial Q_p\right|^2 P(\mathrm{RT})$ and the optically determined k_{nr}(RT) in previously reported data on a variety of chromophores (Figure S34).

k_p [calculated based on the fixed T_1 geometry ($k_{T_1\mathrm{opt}}$)] was determined by using the ADF 2018 package. From the T_1-optimized structure determined by DFT based on Gaussian09 with the B3LYP functional and 6-31G(d) basis set, the dihedral angle between the donating unit and π-conjugated unit (θ_1) for **2h** as well as the biphenyl substituted with a phenoxazine was changed. Regarding **3h** and **4h**, the dihedral angles between the donating unit and π-conjugated unit (θ_1 and θ_2) were changed. The geometries with a variety of θ_1 and/or θ_2 were used for calculating the energy increase compared with the optimized T_1 geometry [$\Delta E(\theta_1, \theta_2)$]. p(RT), depending on independent changes of θ_1 and θ_2 [$p(\mathrm{RT})(\theta_1, \theta_2)$], was calculated by using $p(\mathrm{RT})(\theta_1,\theta_2) = \exp(-\Delta E(\theta_1,\theta_2)/kT)$ based on the Boltzmann distribution. k_p when θ_1 and θ_2 were independently changed (Figure 4c) {i.e., [$k_p(\theta_1, \theta_2)$]} was constructed. The integration of $p(\mathrm{RT})(\theta_1, \theta_2)k_p(\theta_1, \theta_2)$ regarding to θ_1 and θ_2 was calculated as the average value of k_p when θ_1 and θ_2 were distributed based on the Boltzmann theory [$k_{p\ \theta}$]. For calculating k_p in each geometry to determine $k_{p\ T1opt}$ and $k_{p\ \theta}$, the SOC operator within the zeroth-order regular approximation was $\hat{H}_{SO}$. The SOC parameter between the nth-order singlet excited state and

the mth-order triplet excited state $\langle S_n|\hat{H}_{SO}|T_m\rangle$ was treated as a perturbation based on the scalar relativistic orbitals, with the PBE0 functional and TZP basis set. In calculating $\langle S_n|\hat{H}_{SO}|T_m\rangle$, n and m were considered as 10 and 10, respectively. Figure S38 shows information regarding the T_1–S_0 energy ($E_{T_1-S_0}$) in the optimized T_1 geometry to determine $k_{p\ T1opt}$. Figure S39 shows a histogram of the calculated $E_{T_1-S_0}$ vs. the optically measured $E_{T_1-S_0}$. Figure S37 shows data regarding $\Delta E_{T_1-S_0}$ and $\langle T_1|\hat{H}_{SO}|S_0\rangle$ when θ_1 and θ_2 of **3h** were changed. Figures S36, S42, and S43 show data for **2h**, **4h**, and the biphenyl substituted with a phenoxazine, respectively, to determine $k_{p\ \theta}$. Figure S45 shows the change of k_p of **5h** when θ_1 and θ_2 were changed yet the relationship $\theta_1 = \theta_2$ was kept.

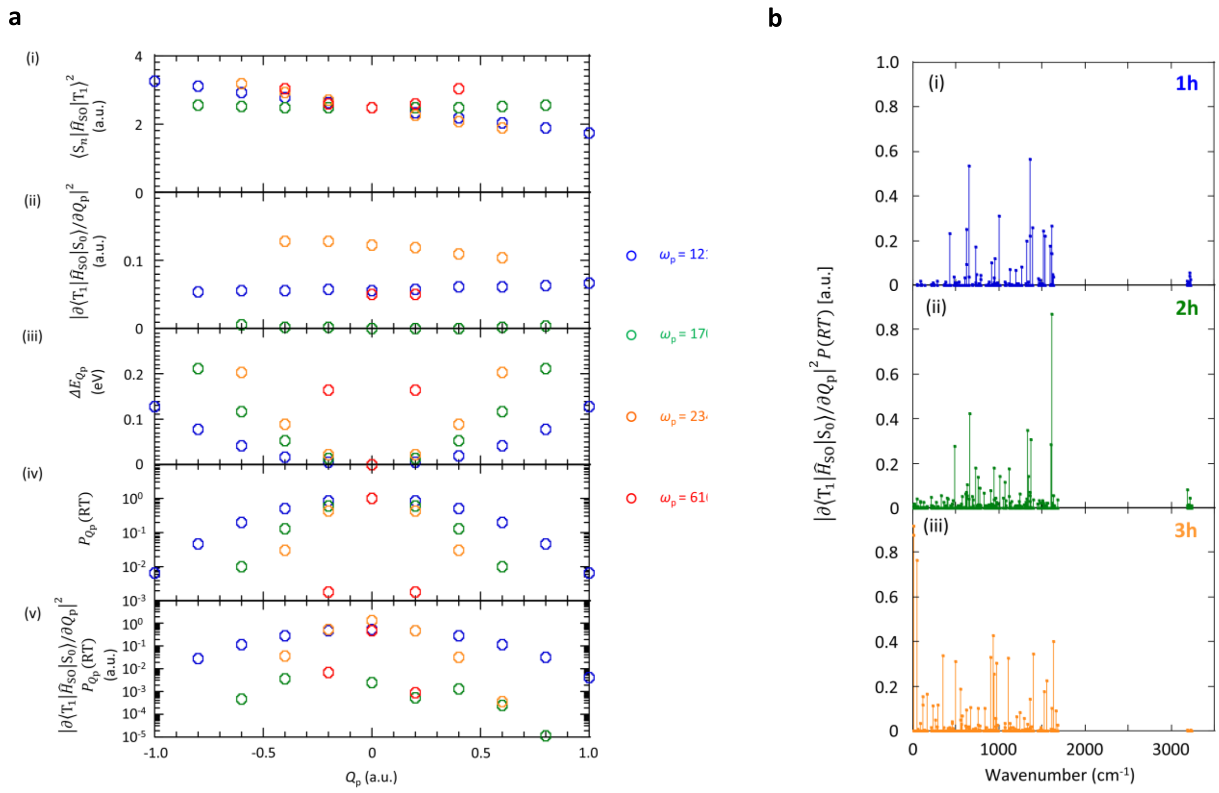

Figure S32. Information to determine $\sum_p|\partial\langle T_1|\hat{H}_{SO}|S_0\rangle/\partial Q_p|^2 P(\mathrm{RT})$ of **3h**. (a) Relationship between parameters related to the T_1–S_0 transition and Q_p of isolated **3h**. (i) $\langle T_1|\hat{H}_{SO}|S_0\rangle_{Q_p}{}^2$, (ii) $|\partial\langle T_1|\hat{H}_{SO}|S_0\rangle_{Q_p}/\partial Q_p|^2$, (iii) ΔE_{Q_p}, (iv) $P_{Q_p}(\mathrm{RT})$, and (v) $\left|\partial\langle T_1|\hat{H}_{SO}|S_0\rangle_{Q_p}/\partial Q_p\right|^2 P_{Q_p}(\mathrm{RT})$. (b) $\left|\frac{\partial\langle T_1|\hat{H}_{SO}|S_0\rangle}{\partial Q_p}\right|^2 P(\mathrm{RT})$ vs. ω_p plots for **1h**–**3h**. Regarding (a), reference S9 shows detailed data for **1h** and **2h**.

Table S6. Photo-physical parameters ($\mathit{\Delta} E_{Q_\mathrm{p}}$, P_{Q_p}(RT), $E_{\mathrm{T_1-S_0}}$, and $\langle \mathrm{T_1} | \hat{H}_{\mathrm{SO}} | \mathrm{S_0} \rangle_{Q_\mathrm{p}}{}^2$) calculated for geometries allowed by normal-mode vibrations with frequency in $\mathrm{T_1}$ at RT for **3h**. ω_p is the wavenumber of the p_th normal vibrational mode in the structure optimized at $\mathrm{T_1}$. Q_p is the mass weighted normal coordinates at $\mathrm{T_1}$. Calculation regarding geometries was stopped when $\mathit{\Delta} E_{Q_\mathrm{p}}$ becomes larger than 0.2 eV because these geometries with $\mathit{\Delta} E_{Q_\mathrm{p}}$ > 0.2 eV are hardly allowed below RT. $E_{\mathrm{T_1-S_0}}$ does not largely change for Q_p with P_{Q_p}(RT) > 0.01, which corresponds to geometries much allowed at RT.

ω_p (cm^{-1})	Q_p (a.u.)	$\mathit{\Delta} E_{Q_\mathrm{p}}$ (eV)	P_{Q_p}(RT)	$\langle \mathrm{T_1} \vert \hat{H}_{\mathrm{SO}} \vert \mathrm{S_0} \rangle_{Q_\mathrm{p}}{}^2$ (cm^{-2})	$E_{\mathrm{T_1-S_0}}$ (eV)
8.859869	-1	0.0021	0.9227	2.28	1.72
8.859869	-0.8	0.0011	0.9566	3.42	1.71
8.859869	-0.6	0.0006	0.9780	3.45	1.71
8.859869	-0.4	0.0002	0.9904	3.49	1.71
8.859869	-0.2	0.0001	0.9971	3.52	1.71
8.859869	0	0.0000	1.0000	3.55	1.71
8.859869	0.2	0.0000	0.9992	3.58	1.71
8.859869	0.4	0.0001	0.9946	3.61	1.70
8.859869	0.6	0.0004	0.9841	3.64	1.70
8.859869	0.8	0.0009	0.9646	3.67	1.70
8.859869	1	0.0018	0.9323	3.71	1.70
13.0857	-1	0.0034	0.8767	3.55	1.71
13.0857	-0.8	0.0018	0.9324	3.56	1.71
13.0857	-0.6	0.0008	0.9676	3.55	1.71
13.0857	-0.4	0.0003	0.9875	3.55	1.71
13.0857	-0.2	0.0001	0.9971	3.55	1.71
13.0857	0	0.0000	1.0000	3.55	1.71
13.0857	0.2	0.0001	0.9965	3.55	1.71
13.0857	0.4	0.0004	0.9864	3.54	1.71
13.0857	0.6	0.0009	0.9658	2.41	1.70
13.0857	0.8	0.0019	0.9302	2.40	1.70
13.0857	1	0.0035	0.8738	2.40	1.70
14.72459	-1	0.0047	0.8327	3.56	1.71
14.72459	-0.8	0.0020	0.9241	3.57	1.71
14.72459	-0.6	0.0007	0.9730	3.56	1.71
14.72459	-0.4	0.0002	0.9933	3.56	1.71
14.72459	-0.2	0.0000	0.9991	3.56	1.71
14.72459	0	0.0000	1.0000	3.55	1.71
14.72459	0.2	0.0000	0.9988	3.56	1.71
14.72459	0.4	0.0002	0.9928	3.56	1.71

14.72459	0.6	0.0007	0.9720	3.56	1.71
14.72459	0.8	0.0021	0.9220	3.56	1.71
14.72459	1	0.0048	0.8283	3.56	1.71
18.43289	-1	0.0109	0.6550	3.61	1.70
18.43289	-0.8	0.0048	0.8298	3.61	1.71
18.43289	-0.6	0.0011	0.9568	3.60	1.71
18.43289	-0.4	0.0009	0.9639	3.59	1.71
18.43289	-0.2	0.0003	1.0116	3.57	1.71
18.43289	0	0.0000	1.0000	3.55	1.71
18.43289	0.2	0.0003	1.0114	3.57	1.71
18.43289	0.4	0.0011	0.9598	3.59	1.71
18.43289	0.6	0.0013	0.9513	3.60	1.71
18.43289	0.8	0.0050	0.8227	3.60	1.71
18.43289	1	0.0114	0.6421	3.47	1.70
20.09279	-1	0.0210	0.4412	3.45	1.70
20.09279	-0.8	0.0097	0.6852	3.47	1.70
20.09279	-0.6	0.0038	0.8620	3.49	1.70
20.09279	-0.4	0.0012	0.9550	3.51	1.70
20.09279	-0.2	0.0002	0.9909	3.53	1.71
20.09279	0	0.0000	1.0000	3.55	1.71
20.09279	0.2	0.0001	0.9945	3.57	1.71
20.09279	0.4	0.0009	0.9640	3.59	1.71
20.09279	0.6	0.0033	0.8786	3.61	1.71
20.09279	0.8	0.0088	0.7091	3.62	1.71
20.09279	1	0.0196	0.4667	3.64	1.71
26.43615	-1	0.0085	0.7190	3.57	1.70
26.43615	-0.8	0.0047	0.8340	3.57	1.71
26.43615	-0.6	0.0022	0.9197	3.57	1.71
26.43615	-0.4	0.0007	0.9713	3.57	1.71
26.43615	-0.2	0.0001	0.9943	3.56	1.71
26.43615	0	0.0000	1.0000	3.55	1.71
26.43615	0.2	0.0002	0.9939	3.56	1.71
26.43615	0.4	0.0008	0.9707	3.56	1.71
26.43615	0.6	0.0022	0.9191	3.57	1.71
26.43615	0.8	0.0047	0.8333	3.57	1.71
26.43615	1	0.0085	0.7179	3.57	1.70
32.51849	-1	0.0177	0.5017	3.60	1.71
32.51849	-0.8	0.0106	0.6632	3.61	1.71
32.51849	-0.6	0.0045	0.8400	3.60	1.71

32.51849	-0.4	0.0010	0.9624	3.59	1.71
32.51849	-0.2	0.0000	1.0000	3.57	1.71
32.51849	0	0.0000	1.0000	3.55	1.71
32.51849	0.2	0.0000	0.9996	3.57	1.71
32.51849	0.4	0.0010	0.9626	3.59	1.71
32.51849	0.6	0.0045	0.8391	3.60	1.71
32.51849	0.8	0.0106	0.6632	3.61	1.71
32.51849	1	0.0177	0.5023	3.60	1.71
36.4705	-1	0.0208	0.4449	3.45	1.73
36.4705	-0.8	0.0120	0.6262	3.48	1.73
36.4705	-0.6	0.0062	0.7863	3.50	1.72
36.4705	-0.4	0.0025	0.9056	3.52	1.72
36.4705	-0.2	0.0006	0.9768	3.54	1.71
36.4705	0	0.0000	1.0000	3.55	1.71
36.4705	0.2	0.0006	0.9769	3.56	1.70
36.4705	0.4	0.0024	0.9098	3.56	1.70
36.4705	0.6	0.0057	0.8001	3.56	1.69
36.4705	0.8	0.0109	0.6534	3.56	1.69
36.4705	1	0.0187	0.4837	3.56	1.69
48.35656	-1	0.0270	0.3494	4.28	1.68
48.35656	-0.8	0.0165	0.5260	4.12	1.68
48.35656	-0.6	0.0090	0.7054	3.97	1.69
48.35656	-0.4	0.0039	0.8589	3.82	1.70
48.35656	-0.2	0.0010	0.9624	3.68	1.70
48.35656	0	0.0000	1.0000	3.55	1.71
48.35656	0.2	0.0009	0.9645	3.42	1.71
48.35656	0.4	0.0039	0.8604	3.30	1.72
48.35656	0.6	0.0091	0.7024	2.17	1.72
48.35656	0.8	0.0170	0.5165	2.09	1.72
48.35656	1	0.0281	0.3348	2.01	1.72
57.4897	-1	0.0411	0.2020	3.46	1.67
57.4897	-0.8	0.0251	0.3757	3.47	1.68
57.4897	-0.6	0.0137	0.5878	3.47	1.69
57.4897	-0.4	0.0059	0.7941	3.49	1.69
57.4897	-0.2	0.0015	0.9443	3.51	1.70
57.4897	0	0.0000	1.0000	3.55	1.71
57.4897	0.2	0.0014	0.9458	3.59	1.71
57.4897	0.4	0.0059	0.7958	3.64	1.72
57.4897	0.6	0.0136	0.5885	3.70	1.72

57.4897	0.8	0.0252	0.3749	3.77	1.73
57.4897	1	0.0413	0.2002	3.85	1.74
84.17174	-1	0.0771	0.0498	3.94	1.63
84.17174	-0.8	0.0454	0.1707	3.88	1.65
84.17174	-0.6	0.0238	0.3962	3.82	1.67
84.17174	-0.4	0.0100	0.6779	3.74	1.68
84.17174	-0.2	0.0024	0.9108	3.65	1.70
84.17174	0	0.0000	1.0000	3.55	1.71
84.17174	0.2	0.0024	0.9110	3.44	1.72
84.17174	0.4	0.0098	0.6840	3.33	1.72
84.17174	0.6	0.0229	0.4104	3.20	1.73
84.17174	0.8	0.0431	0.1866	3.08	1.73
84.17174	1	0.0724	0.0597	2.95	1.73
85.83134	-1	0.0799	0.0446	3.65	1.71
85.83134	-0.8	0.0492	0.1474	3.63	1.71
85.83134	-0.6	0.0271	0.3480	3.61	1.71
85.83134	-0.4	0.0111	0.6491	3.59	1.71
85.83134	-0.2	0.0018	0.9318	3.57	1.71
85.83134	0	0.0000	1.0000	3.55	1.71
85.83134	0.2	0.0018	0.9319	3.57	1.71
85.83134	0.4	0.0110	0.6506	3.59	1.71
85.83134	0.6	0.0270	0.3490	3.61	1.71
85.83134	0.8	0.0491	0.1481	3.63	1.71
85.83134	1	0.0796	0.0451	3.65	1.71
96.24173	-1	0.0918	0.0281	3.63	1.68
96.24173	-0.8	0.0559	0.1135	3.61	1.69
96.24173	-0.6	0.0303	0.3072	3.59	1.70
96.24173	-0.4	0.0127	0.6100	3.58	1.70
96.24173	-0.2	0.0025	0.9074	3.57	1.71
96.24173	0	0.0000	1.0000	3.55	1.71
96.24173	0.2	0.0025	0.9073	3.57	1.71
96.24173	0.4	0.0127	0.6107	3.58	1.70
96.24173	0.6	0.0302	0.3082	3.59	1.70
96.24173	0.8	0.0557	0.1143	3.61	1.69
96.24173	1	0.0914	0.0285	3.63	1.68
118.5852	-1	0.1524	0.0027	2.31	1.71
118.5852	-0.8	0.0961	0.0238	2.54	1.71
118.5852	-0.6	0.0534	0.1253	2.78	1.71
118.5852	-0.4	0.0235	0.4008	3.03	1.71

118.5852	-0.2	0.0059	0.7963	3.29	1.71
118.5852	0	0.0000	1.0000	3.55	1.71
118.5852	0.2	0.0057	0.8009	3.81	1.71
118.5852	0.4	0.0229	0.4102	4.09	1.70
118.5852	0.6	0.0517	0.1336	4.36	1.70
118.5852	0.8	0.0925	0.0273	4.64	1.70
118.5852	1	0.1458	0.0034	4.93	1.69
119.6082	-1	0.1224	0.0085	3.50	1.70
119.6082	-0.8	0.0740	0.0562	3.53	1.70
119.6082	-0.6	0.0396	0.2138	3.55	1.70
119.6082	-0.4	0.0170	0.5167	3.55	1.71
119.6082	-0.2	0.0041	0.8511	3.56	1.71
119.6082	0	0.0000	1.0000	3.55	1.71
119.6082	0.2	0.0042	0.8507	3.55	1.71
119.6082	0.4	0.0170	0.5161	3.55	1.71
119.6082	0.6	0.0397	0.2133	3.53	1.70
119.6082	0.8	0.0741	0.0559	3.51	1.70
119.6082	1	0.1226	0.0085	3.48	1.70
121.4602	-1	0.1282	0.0068	4.70	1.70
121.4602	-0.8	0.0779	0.0482	4.46	1.70
121.4602	-0.6	0.0420	0.1952	4.22	1.71
121.4602	-0.4	0.0181	0.4953	3.99	1.71
121.4602	-0.2	0.0044	0.8422	3.77	1.71
121.4602	0	0.0000	1.0000	3.55	1.71
121.4602	0.2	0.0045	0.8394	3.33	1.71
121.4602	0.4	0.0183	0.4911	3.12	1.71
121.4602	0.6	0.0424	0.1920	2.92	1.70
121.4602	0.8	0.0786	0.0470	2.71	1.70
121.4602	1	0.1291	0.0066	2.51	1.70
132.0507	-1	0.1677	0.0015	3.58	1.69
132.0507	-0.8	0.1042	0.0173	3.58	1.70
132.0507	-0.6	0.0572	0.1078	3.57	1.70
132.0507	-0.4	0.0249	0.3800	3.57	1.70
132.0507	-0.2	0.0061	0.7890	3.56	1.71
132.0507	0	0.0000	1.0000	3.55	1.71
132.0507	0.2	0.0061	0.7882	3.56	1.71
132.0507	0.4	0.0249	0.3789	3.57	1.70
132.0507	0.6	0.0574	0.1072	3.57	1.70
132.0507	0.8	0.1045	0.0171	3.57	1.70

132.0507	1	0.1681	0.0014	3.58	1.69
138.2501	-1	0.2138	0.0002	3.61	1.75
138.2501	-0.8	0.1341	0.0054	3.63	1.74
138.2501	-0.6	0.0742	0.0557	3.63	1.73
138.2501	-0.4	0.0325	0.2819	3.61	1.73
138.2501	-0.2	0.0081	0.7310	3.59	1.72
138.2501	0	0.0000	1.0000	3.55	1.71
138.2501	0.2	0.0081	0.7291	3.49	1.70
138.2501	0.4	0.0326	0.2809	3.43	1.68
138.2501	0.6	0.0742	0.0556	3.36	1.67
138.2501	0.8	0.1341	0.0054	3.27	1.66
138.2501	1	0.2137	0.0002	3.18	1.64
145.1135	-1	0.2125	0.0003	3.35	1.69
145.1135	-0.8	0.1332	0.0056	3.43	1.70
145.1135	-0.6	0.0730	0.0585	3.48	1.70
145.1135	-0.4	0.0313	0.2953	3.53	1.70
145.1135	-0.2	0.0076	0.7445	3.55	1.71
145.1135	0	0.0000	1.0000	3.55	1.71
145.1135	0.2	0.0076	0.7436	3.55	1.71
145.1135	0.4	0.0314	0.2951	3.53	1.70
145.1135	0.6	0.0729	0.0586	3.48	1.70
145.1135	0.8	0.1330	0.0056	3.42	1.70
145.1135	1	0.2122	0.0003	3.35	1.69
166.7538	-1	0.2747	0.0000	2.27	1.71
166.7538	-0.8	0.1690	0.0014	2.47	1.72
166.7538	-0.6	0.0917	0.0282	2.70	1.72
166.7538	-0.4	0.0395	0.2148	2.96	1.72
166.7538	-0.2	0.0096	0.6876	3.24	1.71
166.7538	0	0.0000	1.0000	3.55	1.71
166.7538	0.2	0.0094	0.6938	3.84	1.70
166.7538	0.4	0.0372	0.2349	4.26	1.69
166.7538	0.6	0.0836	0.0387	4.66	1.67
166.7538	0.8	0.1491	0.0030	5.09	1.66
166.7538	1	0.2352	0.0001	5.56	1.64
169.8765	-0.8	0.2116	0.0003	3.70	1.71
169.8765	-0.6	0.1181	0.0101	3.64	1.71
169.8765	-0.4	0.0522	0.1313	3.59	1.71
169.8765	-0.2	0.0130	0.6028	3.56	1.71
169.8765	0	0.0000	1.0000	3.55	1.71

169.8765	0.2	0.0130	0.6037	3.56	1.71
169.8765	0.4	0.0521	0.1315	3.58	1.71
169.8765	0.6	0.1181	0.0101	3.63	1.71
169.8765	0.8	0.2117	0.0003	3.69	1.71
218.6663	-0.6	0.2052	0.0003	3.98	1.76
218.6663	-0.4	0.0910	0.0289	3.80	1.75
218.6663	-0.2	0.0227	0.4127	3.66	1.73
218.6663	0	0.0000	1.0000	3.55	1.71
218.6663	0.2	0.0228	0.4125	3.47	1.68
218.6663	0.4	0.0911	0.0288	3.41	1.65
218.6663	0.6	0.2055	0.0003	3.37	1.62
224.9159	-0.8	0.3456	0.0000	3.49	1.68
224.9159	-0.6	0.1918	0.0006	3.52	1.69
224.9159	-0.4	0.0843	0.0375	3.54	1.70
224.9159	-0.2	0.0209	0.4425	3.56	1.71
224.9159	0	0.0000	1.0000	3.55	1.71
224.9159	0.2	0.0209	0.4426	3.56	1.71
224.9159	0.4	0.0844	0.0375	3.54	1.70
224.9159	0.6	0.1920	0.0006	3.52	1.69
224.9159	0.8	0.3460	0.0000	3.49	1.68
231.1359	-0.6	0.2035	0.0004	3.53	1.70
231.1359	-0.4	0.0899	0.0302	3.55	1.71
231.1359	-0.2	0.0224	0.4180	3.56	1.71
231.1359	0	0.0000	1.0000	3.55	1.71
231.1359	0.2	0.0224	0.4186	3.56	1.71
231.1359	0.4	0.0899	0.0303	3.55	1.71
231.1359	0.6	0.2034	0.0004	3.54	1.70
234.485	-0.6	0.2038	0.0004	4.57	1.76
234.485	-0.4	0.0903	0.0297	4.22	1.74
234.485	-0.2	0.0225	0.4158	3.87	1.73
234.485	0	0.0000	1.0000	3.55	1.71
234.485	0.2	0.0225	0.4158	3.24	1.69
234.485	0.4	0.0903	0.0298	2.96	1.66
234.485	0.6	0.2036	0.0004	2.70	1.64
263.8402	-0.6	0.2624	0.0000	3.42	1.68
263.8402	-0.4	0.1153	0.0113	3.50	1.70
263.8402	-0.2	0.0286	0.3287	3.55	1.70
263.8402	0	0.0000	1.0000	3.55	1.71
263.8402	0.2	0.0286	0.3284	3.54	1.70

263.8402	0.4	0.1153	0.0112	3.50	1.70
263.8402	0.6	0.2625	0.0000	3.42	1.68
266.7894	-0.6	0.2732	0.0000	4.31	1.59
266.7894	-0.4	0.1212	0.0089	4.04	1.63
266.7894	-0.2	0.0304	0.3067	3.78	1.67
266.7894	0	0.0000	1.0000	3.55	1.71
266.7894	0.2	0.0311	0.2981	3.33	1.74
266.7894	0.4	0.1263	0.0073	3.12	1.77
266.7894	0.6	0.2897	0.0000	2.93	1.80
269.1882	-0.6	0.2499	0.0001	3.50	1.78
269.1882	-0.4	0.1098	0.0139	3.50	1.76
269.1882	-0.2	0.0273	0.3454	3.51	1.73
269.1882	0	0.0000	1.0000	3.55	1.71
269.1882	0.2	0.0273	0.3453	3.61	1.68
269.1882	0.4	0.1104	0.0136	3.69	1.65
269.1882	0.6	0.2522	0.0001	3.81	1.62
269.8252	-0.6	0.2519	0.0001	3.61	1.71
269.8252	-0.4	0.1098	0.0139	3.58	1.71
269.8252	-0.2	0.0271	0.3477	3.57	1.71
269.8252	0	0.0000	1.0000	3.55	1.71
269.8252	0.2	0.0272	0.3471	3.57	1.71
269.8252	0.4	0.1101	0.0138	3.58	1.71
269.8252	0.6	0.2526	0.0001	3.61	1.71
281.0796	-0.6	0.2667	0.0000	3.16	1.82
281.0796	-0.4	0.1190	0.0098	3.29	1.78
281.0796	-0.2	0.0299	0.3121	3.42	1.75
281.0796	0	0.0000	1.0000	3.55	1.71
281.0796	0.2	0.0304	0.3063	3.69	1.67
281.0796	0.4	0.1229	0.0084	3.84	1.62
281.0796	0.6	0.2801	0.0000	4.00	1.57
295.9453	-0.6	0.3269	0.0000	3.51	1.71
295.9453	-0.4	0.1438	0.0037	3.54	1.71
295.9453	-0.2	0.0357	0.2494	3.56	1.71
295.9453	0	0.0000	1.0000	3.55	1.71
295.9453	0.2	0.0357	0.2494	3.55	1.71
295.9453	0.4	0.1438	0.0037	3.53	1.71
295.9453	0.6	0.3268	0.0000	3.49	1.71
296.0523	-0.6	0.3318	0.0000	2.56	1.75
296.0523	-0.4	0.1449	0.0035	2.86	1.74

296.0523	-0.2	0.0357	0.2491	3.20	1.72
296.0523	0	0.0000	1.0000	3.55	1.71
296.0523	0.2	0.0351	0.2548	3.92	1.69
296.0523	0.4	0.1400	0.0043	4.32	1.67
296.0523	0.6	0.3151	0.0000	4.73	1.65
301.9013	-0.6	0.3428	0.0000	3.54	1.70
301.9013	-0.4	0.1520	0.0027	3.54	1.70
301.9013	-0.2	0.0379	0.2284	3.55	1.71
301.9013	0	0.0000	1.0000	3.55	1.71
301.9013	0.2	0.0379	0.2286	3.55	1.71
301.9013	0.4	0.1519	0.0027	3.54	1.70
301.9013	0.6	0.3428	0.0000	3.54	1.70
330.7734	-0.6	0.4209	0.0000	3.64	1.68
330.7734	-0.4	0.1854	0.0007	3.60	1.70
330.7734	-0.2	0.0461	0.1662	3.57	1.70
330.7734	0	0.0000	1.0000	3.55	1.71
330.7734	0.2	0.0461	0.1662	3.57	1.70
330.7734	0.4	0.1854	0.0007	3.59	1.70
330.7734	0.6	0.4209	0.0000	3.63	1.68
351.8719	-0.4	0.2248	0.0002	5.07	1.61
351.8719	-0.2	0.0559	0.1134	4.27	1.66
351.8719	0	0.0000	1.0000	3.55	1.71
351.8719	0.2	0.0555	0.1151	2.91	1.74
351.8719	0.4	0.2219	0.0002	2.36	1.77
361.1028	-0.4	0.2384	0.0001	3.87	1.52
361.1028	-0.2	0.0595	0.0986	3.69	1.62
361.1028	0	0.0000	1.0000	3.55	1.71
361.1028	0.2	0.0596	0.0983	3.43	1.79
361.1028	0.4	0.2388	0.0001	3.34	1.87
382.8671	-0.4	0.2598	0.0000	3.63	1.70
382.8671	-0.2	0.0639	0.0830	3.58	1.71
382.8671	0	0.0000	1.0000	3.55	1.71
382.8671	0.2	0.0639	0.0830	3.57	1.71
382.8671	0.4	0.2598	0.0000	3.63	1.70
387.8236	-0.4	0.2895	0.0000	3.62	1.68
387.8236	-0.2	0.0719	0.0609	3.57	1.70
387.8236	0	0.0000	1.0000	3.55	1.71
387.8236	0.2	0.0712	0.0626	3.55	1.71
387.8236	0.4	0.2838	0.0000	3.57	1.70

395.1883	-0.4	0.3038	0.0000	3.50	1.58
395.1883	-0.2	0.0766	0.0506	3.53	1.64
395.1883	0	0.0000	1.0000	3.55	1.71
395.1883	0.2	0.0782	0.0477	3.58	1.77
395.1883	0.4	0.3156	0.0000	3.61	1.83
397.5881	-0.4	0.2972	0.0000	3.58	1.70
397.5881	-0.2	0.0739	0.0564	3.57	1.71
397.5881	0	0.0000	1.0000	3.55	1.71
397.5881	0.2	0.0739	0.0563	3.57	1.71
397.5881	0.4	0.2972	0.0000	3.58	1.70
410.4327	-0.4	0.2530	0.0001	3.78	1.55
410.4327	-0.2	0.0632	0.0855	3.68	1.63
410.4327	0	0.0000	1.0000	3.55	1.71
410.4327	0.2	0.0633	0.0852	3.37	1.78
410.4327	0.4	0.2538	0.0001	3.12	1.85
414.8951	-0.4	0.2644	0.0000	3.57	1.70
414.8951	-0.2	0.0651	0.0793	3.57	1.71
414.8951	0	0.0000	1.0000	3.55	1.71
414.8951	0.2	0.0651	0.0794	3.57	1.71
414.8951	0.4	0.2644	0.0000	3.58	1.70
431.6056	-0.4	0.2222	0.0002	3.68	1.76
431.6056	-0.2	0.0554	0.1157	3.68	1.74
431.6056	0	0.0000	1.0000	3.55	1.71
431.6056	0.2	0.0557	0.1143	3.31	1.67
431.6056	0.4	0.2242	0.0002	2.98	1.63
444.6936	-0.4	0.2548	0.0000	3.60	1.70
444.6936	-0.2	0.0630	0.0862	3.57	1.70
444.6936	0	0.0000	1.0000	3.55	1.71
444.6936	0.2	0.0630	0.0861	3.57	1.70
444.6936	0.4	0.2550	0.0000	3.59	1.70
445.4891	-0.4	0.2350	0.0001	3.51	1.70
445.4891	-0.2	0.0585	0.1025	3.55	1.71
445.4891	0	0.0000	1.0000	3.55	1.71
445.4891	0.2	0.0585	0.1027	3.55	1.71
445.4891	0.4	0.2349	0.0001	3.50	1.70
449.6042	-0.4	0.3353	0.0000	3.77	1.67
449.6042	-0.2	0.0835	0.0388	3.66	1.69
449.6042	0	0.0000	1.0000	3.55	1.71
449.6042	0.2	0.0830	0.0396	3.43	1.72

449.6042	0.4	0.3309	0.0000	3.32	1.74
456.9026	-0.4	0.3399	0.0000	4.09	1.65
456.9026	-0.2	0.0846	0.0372	3.81	1.68
456.9026	0	0.0000	1.0000	3.55	1.71
456.9026	0.2	0.0838	0.0383	3.30	1.73
456.9026	0.4	0.3340	0.0000	3.06	1.75
457.4321	-0.4	0.2639	0.0000	3.58	1.70
457.4321	-0.2	0.0658	0.0772	3.56	1.71
457.4321	0	0.0000	1.0000	3.55	1.71
457.4321	0.2	0.0658	0.0771	3.57	1.71
457.4321	0.4	0.2639	0.0000	3.58	1.70
461.1784	-0.4	0.2250	0.0002	3.12	1.71
461.1784	-0.2	0.0555	0.1152	3.34	1.71
461.1784	0	0.0000	1.0000	3.55	1.71
461.1784	0.2	0.0551	0.1172	3.75	1.71
461.1784	0.4	0.2218	0.0002	3.92	1.71
461.1971	-0.4	0.2276	0.0001	3.51	1.71
461.1971	-0.2	0.0564	0.1115	3.54	1.71
461.1971	0	0.0000	1.0000	3.55	1.71
461.1971	0.2	0.0563	0.1117	3.57	1.71
461.1971	0.4	0.2272	0.0001	3.56	1.71
470.5751	-0.4	0.2405	0.0001	3.28	1.71
470.5751	-0.2	0.0596	0.0984	3.43	1.71
470.5751	0	0.0000	1.0000	3.55	1.71
470.5751	0.2	0.0595	0.0988	3.64	1.71
470.5751	0.4	0.2402	0.0001	3.71	1.71
477.0072	-0.4	0.2604	0.0000	3.54	1.71
477.0072	-0.2	0.0646	0.0810	3.55	1.71
477.0072	0	0.0000	1.0000	3.55	1.71
477.0072	0.2	0.0646	0.0810	3.54	1.71
477.0072	0.4	0.2604	0.0000	3.53	1.71
492.0799	-0.4	0.2825	0.0000	5.02	1.65
492.0799	-0.2	0.0705	0.0644	4.27	1.68
492.0799	0	0.0000	1.0000	3.55	1.71
492.0799	0.2	0.0710	0.0630	2.88	1.72
492.0799	0.4	0.2867	0.0000	2.29	1.72
515.3639	-0.4	0.3707	0.0000	3.52	1.75
515.3639	-0.2	0.0925	0.0273	3.57	1.73
515.3639	0	0.0000	1.0000	3.55	1.71

515.3639	0.2	0.0925	0.0273	3.45	1.68
515.3639	0.4	0.3711	0.0000	3.30	1.64
518.6098	-0.4	0.3354	0.0000	3.59	1.69
518.6098	-0.2	0.0835	0.0388	3.57	1.70
518.6098	0	0.0000	1.0000	3.55	1.71
518.6098	0.2	0.0835	0.0388	3.56	1.70
518.6098	0.4	0.3354	0.0000	3.59	1.69
537.3804	-0.4	0.5256	0.0000	3.37	1.69
537.3804	-0.2	0.1313	0.0060	3.51	1.70
537.3804	0	0.0000	1.0000	3.55	1.71
537.3804	0.2	0.1313	0.0060	3.51	1.70
537.3804	0.4	0.5255	0.0000	3.37	1.69
550.8598	-0.4	0.4317	0.0000	2.54	1.67
550.8598	-0.2	0.1076	0.0152	3.01	1.69
550.8598	0	0.0000	1.0000	3.55	1.71
550.8598	0.2	0.1076	0.0152	4.16	1.71
550.8598	0.4	0.4313	0.0000	4.86	1.69
551.4696	-0.4	0.3867	0.0000	3.57	1.70
551.4696	-0.2	0.0962	0.0236	3.57	1.71
551.4696	0	0.0000	1.0000	3.55	1.71
551.4696	0.2	0.0962	0.0236	3.56	1.71
551.4696	0.4	0.3868	0.0000	3.55	1.70
551.7307	-0.4	0.3944	0.0000	4.15	1.70
551.7307	-0.2	0.0983	0.0218	3.84	1.70
551.7307	0	0.0000	1.0000	3.55	1.71
551.7307	0.2	0.0983	0.0218	3.27	1.71
551.7307	0.4	0.3948	0.0000	3.01	1.70
557.7318	-0.4	0.4505	0.0000	3.54	1.71
557.7318	-0.2	0.1123	0.0127	3.55	1.71
557.7318	0	0.0000	1.0000	3.55	1.71
557.7318	0.2	0.1123	0.0127	3.55	1.71
557.7318	0.4	0.4505	0.0000	3.53	1.71
559.8926	-0.4	0.4210	0.0000	3.13	1.71
559.8926	-0.2	0.1048	0.0169	3.33	1.71
559.8926	0	0.0000	1.0000	3.55	1.71
559.8926	0.2	0.1050	0.0168	3.78	1.70
559.8926	0.4	0.4217	0.0000	4.04	1.70
567.2075	-0.4	0.5474	0.0000	3.51	1.70
567.2075	-0.2	0.1367	0.0049	3.54	1.70

567.2075	0	0.0000	1.0000	3.55	1.71
567.2075	0.2	0.1367	0.0049	3.54	1.70
567.2075	0.4	0.5474	0.0000	3.51	1.70
570.7419	-0.4	0.5258	0.0000	4.24	1.71
570.7419	-0.2	0.1313	0.0060	3.89	1.71
570.7419	0	0.0000	1.0000	3.55	1.71
570.7419	0.2	0.1315	0.0060	3.22	1.70
570.7419	0.4	0.5273	0.0000	2.90	1.68
596.6787	-0.2	0.2065	0.0003	3.61	1.69
596.6787	0	0.0000	1.0000	3.55	1.71
596.6787	0.2	0.2065	0.0003	3.61	1.69
615.5932	-0.4	0.6533	0.0000	4.37	1.63
615.5932	-0.2	0.1627	0.0018	3.75	1.69
615.5932	0	0.0000	1.0000	3.55	1.71
615.5932	0.2	0.1628	0.0018	3.75	1.69
615.5932	0.4	0.6535	0.0000	4.37	1.63
620.205	-0.4	0.7456	0.0000	3.24	1.68
620.205	-0.2	0.1860	0.0007	3.38	1.70
620.205	0	0.0000	1.0000	3.55	1.71
620.205	0.2	0.1858	0.0007	3.75	1.71
620.205	0.4	0.7435	0.0000	4.01	1.70
622.8894	-0.4	0.6852	0.0000	4.24	1.66
622.8894	-0.2	0.1709	0.0013	3.72	1.69
622.8894	0	0.0000	1.0000	3.55	1.71
622.8894	0.2	0.1709	0.0013	3.72	1.69
622.8894	0.4	0.6852	0.0000	4.23	1.66
623.7169	-0.2	0.2205	0.0002	3.96	1.71
623.7169	0	0.0000	1.0000	3.55	1.71
623.7169	0.2	0.2222	0.0002	3.16	1.70
631.8451	-0.2	0.2103	0.0003	3.56	1.71
631.8451	0	0.0000	1.0000	3.55	1.71
631.8451	0.2	0.2103	0.0003	3.55	1.71
636.4578	-0.2	0.2124	0.0003	3.55	1.71
636.4578	0	0.0000	1.0000	3.55	1.71
636.4578	0.2	0.2120	0.0003	3.51	1.70
646.5336	-0.4	0.7788	0.0000	3.60	1.69
646.5336	-0.2	0.1946	0.0005	3.57	1.70
646.5336	0	0.0000	1.0000	3.55	1.71
646.5336	0.2	0.1946	0.0005	3.57	1.70

646.5336	0.4	0.7789	0.0000	3.60	1.69
648.8627	-0.4	0.5964	0.0000	3.25	1.67
648.8627	-0.2	0.1485	0.0031	3.36	1.69
648.8627	0	0.0000	1.0000	3.55	1.71
648.8627	0.2	0.1483	0.0031	3.85	1.71
648.8627	0.4	0.5947	0.0000	4.31	1.70
649.5932	-0.4	0.6321	0.0000	4.07	1.68
649.5932	-0.2	0.1576	0.0022	3.69	1.70
649.5932	0	0.0000	1.0000	3.55	1.71
649.5932	0.2	0.1576	0.0022	3.69	1.70
649.5932	0.4	0.6321	0.0000	4.08	1.68
667.1871	-0.2	0.2244	0.0002	3.14	1.68
667.1871	0	0.0000	1.0000	3.55	1.71
667.1871	0.2	0.2244	0.0002	4.01	1.72
672.9651	-0.4	0.7927	0.0000	3.60	1.69
672.9651	-0.2	0.1980	0.0004	3.57	1.70
672.9651	0	0.0000	1.0000	3.55	1.71
672.9651	0.2	0.1980	0.0004	3.57	1.70
672.9651	0.4	0.7927	0.0000	3.61	1.69
688.9307	-0.2	0.2234	0.0002	3.51	1.72
688.9307	0	0.0000	1.0000	3.55	1.71
688.9307	0.2	0.2215	0.0002	3.57	1.69
697.8024	-0.2	0.2012	0.0004	3.55	1.71
697.8024	0	0.0000	1.0000	3.55	1.71
697.8024	0.2	0.2011	0.0004	3.55	1.71
698.9684	-0.4	0.7924	0.0000	3.74	1.72
698.9684	-0.2	0.1949	0.0005	3.65	1.71
698.9684	0	0.0000	1.0000	3.55	1.71
698.9684	0.2	0.1961	0.0005	3.44	1.70
698.9684	0.4	0.8024	0.0000	3.31	1.70
701.8728	-0.2	0.2096	0.0003	3.55	1.71
701.8728	0	0.0000	1.0000	3.55	1.71
701.8728	0.2	0.2095	0.0003	3.55	1.71
705.0848	-0.2	0.2206	0.0002	3.66	1.73
705.0848	0	0.0000	1.0000	3.55	1.71
705.0848	0.2	0.2196	0.0002	3.40	1.68
705.6599	-0.4	0.7897	0.0000	3.49	1.71
705.6599	-0.2	0.1940	0.0005	3.54	1.71
705.6599	0	0.0000	1.0000	3.55	1.71

705.6599	0.2	0.1942	0.0005	3.53	1.71
705.6599	0.4	0.7910	0.0000	3.47	1.71
705.8665	-0.4	0.8073	0.0000	3.36	1.70
705.8665	-0.2	0.1981	0.0004	3.48	1.70
705.8665	0	0.0000	1.0000	3.55	1.71
705.8665	0.2	0.1969	0.0005	3.57	1.71
705.8665	0.4	0.7978	0.0000	3.56	1.72
731.9147	-0.4	0.6540	0.0000	3.34	1.68
731.9147	-0.2	0.1615	0.0019	3.42	1.70
731.9147	0	0.0000	1.0000	3.55	1.71
731.9147	0.2	0.1610	0.0019	3.73	1.70
731.9147	0.4	0.6507	0.0000	3.95	1.68
736.8057	-0.2	0.2292	0.0001	3.58	1.70
736.8057	0	0.0000	1.0000	3.55	1.71
736.8057	0.2	0.2293	0.0001	3.58	1.70
752.742	-0.2	0.2450	0.0001	3.56	1.71
752.742	0.2	0.2450	0.0001	3.56	1.71
758.8225	-0.4	0.3081	0.0000	4.38	1.71
758.8225	-0.2	0.0758	0.0523	3.95	1.70
758.8225	0.2	0.0757	0.0525	3.18	1.70
758.8225	0.4	0.3078	0.0000	2.85	1.71
759.3214	-0.4	0.2175	0.0002	3.56	1.71
759.3214	-0.2	0.0527	0.1285	3.56	1.70
759.3214	0	0.0000	1.0000	3.55	1.71
759.3214	0.2	0.0527	0.1285	3.54	1.71
759.3214	0.4	0.2176	0.0002	3.54	1.71
759.5111	-0.4	0.2470	0.0001	3.18	1.71
759.5111	-0.2	0.0606	0.0947	3.36	1.71
759.5111	0	0.0000	1.0000	3.55	1.70
759.5111	0.2	0.0605	0.0949	3.75	1.71
759.5111	0.4	0.2469	0.0001	3.95	1.71
761.6465	-0.2	0.0532	0.1263	3.55	1.71
761.6465	0	0.0000	1.0000	3.55	1.71
761.6465	0.2	0.0531	0.1268	3.55	1.71
761.6915	-0.4	0.2196	0.0002	3.57	1.71
761.6915	-0.2	0.0531	0.1264	3.56	1.71
761.6915	0	0.0000	1.0000	3.55	1.71
761.6915	0.2	0.0531	0.1264	3.53	1.71
761.6915	0.4	0.2196	0.0002	3.52	1.71

768.9527	-0.4	0.2581	0.0000	3.63	1.71
768.9527	-0.2	0.0614	0.0918	3.57	1.71
768.9527	0	0.0000	1.0000	3.55	1.71
768.9527	0.2	0.0615	0.0912	3.57	1.71
768.9527	0.4	0.2592	0.0000	3.63	1.70
775.3806	-0.2	0.2228	0.0002	3.74	1.71
775.3806	0	0.0000	1.0000	3.55	1.71
775.3806	0.2	0.2261	0.0002	3.37	1.71
777.0726	-0.4	0.4151	0.0000	3.40	1.70
777.0726	-0.2	0.1012	0.0195	3.47	1.72
777.0726	0	0.0000	1.0000	3.55	1.71
777.0726	0.2	0.1012	0.0195	3.64	1.69
777.0726	0.4	0.4151	0.0000	3.73	1.70
779.8194	-0.4	0.5890	0.0000	3.86	1.71
779.8194	-0.2	0.1454	0.0035	3.64	1.71
779.8194	0	0.0000	1.0000	3.55	1.70
779.8194	0.4	0.5886	0.0000	3.87	1.68
816.0997	-0.2	0.2986	0.0000	3.55	1.68
816.0997	0	0.0000	1.0000	3.55	1.70
834.3448	-0.4	0.3534	0.0000	4.38	1.71
834.3448	-0.2	0.0839	0.0382	3.96	1.70
834.3448	0	0.0000	1.0000	3.55	1.68
834.3448	0.2	0.0837	0.0385	3.12	1.71
834.3448	0.4	0.3517	0.0000	2.68	1.71
834.866	-0.4	0.4376	0.0000	3.48	1.71
834.866	-0.2	0.1052	0.0167	3.55	1.73
834.866	0	0.0000	1.0000	3.55	1.72
834.866	0.2	0.1050	0.0168	3.51	1.71
834.866	0.4	0.4360	0.0000	3.39	1.68
843.6419	-0.2	0.2189	0.0002	3.71	1.65
843.6419	0	0.0000	1.0000	3.55	1.70
843.6419	0.2	0.2178	0.0002	3.41	1.71
849.6973	-0.4	0.6526	0.0000	3.66	1.71
849.6973	-0.2	0.1618	0.0018	3.59	1.70
849.6973	0	0.0000	1.0000	3.55	1.69
849.6973	0.2	0.1617	0.0018	3.59	1.72
849.6973	0.4	0.6521	0.0000	3.66	1.71
856.6311	-0.4	0.3092	0.0000	3.50	1.69
856.6311	-0.2	0.0748	0.0544	3.53	1.70

856.6311	0	0.0000	1.0000	3.55	1.71
856.6311	0.2	0.0746	0.0548	3.56	1.71
856.6311	0.4	0.3081	0.0000	3.57	1.71
856.7113	-0.4	0.3096	0.0000	3.54	1.70
856.7113	-0.2	0.0748	0.0544	3.55	1.71
856.7113	0	0.0000	1.0000	3.55	1.71
856.7113	0.2	0.0749	0.0543	3.55	1.71
856.7113	0.4	0.3101	0.0000	3.54	1.71
859.2003	-0.4	0.3156	0.0000	3.25	1.71
859.2003	-0.2	0.0766	0.0507	3.40	1.71
859.2003	0	0.0000	1.0000	3.55	1.71
859.2003	0.2	0.0768	0.0504	3.69	1.71
859.2003	0.4	0.3169	0.0000	3.83	1.71
859.2441	-0.4	0.3190	0.0000	3.56	1.71
859.2441	-0.2	0.0774	0.0492	3.56	1.71
859.2441	0	0.0000	1.0000	3.55	1.71
859.2441	0.2	0.0774	0.0492	3.55	1.71
859.2441	0.4	0.3192	0.0000	3.53	1.71
882.9593	-0.4	0.5787	0.0000	3.45	1.71
882.9593	-0.2	0.1432	0.0038	3.50	1.71
882.9593	0	0.0000	1.0000	3.55	1.71
882.9593	0.2	0.1431	0.0038	3.59	1.71
882.9593	0.4	0.5783	0.0000	3.65	1.71
884.8035	-0.2	0.2604	0.0000	3.57	1.71
884.8035	0	0.0000	1.0000	3.55	1.72
884.8035	0.2	0.2603	0.0000	3.57	1.71
888.2276	-0.4	0.3467	0.0000	3.59	1.71
888.2276	-0.2	0.0838	0.0383	3.56	1.70
888.2276	0	0.0000	1.0000	3.55	1.69
888.2276	0.2	0.0838	0.0384	3.56	1.71
888.2276	0.4	0.3463	0.0000	3.58	1.71
888.9262	-0.4	0.4454	0.0000	3.73	1.71
888.9262	-0.2	0.1089	0.0144	3.62	1.70
888.9262	0	0.0000	1.0000	3.55	1.71
888.9262	0.2	0.1085	0.0147	3.49	1.71
888.9262	0.4	0.4423	0.0000	3.42	1.71
902.7939	-0.4	0.4847	0.0000	3.68	1.70
902.7939	-0.2	0.1136	0.0120	3.61	1.73
902.7939	0	0.0000	1.0000	3.55	1.72

902.7939	0.2	0.1141	0.0118	3.52	1.71
902.7939	0.4	0.4879	0.0000	3.50	1.70
903.1654	-0.4	0.4600	0.0000	5.19	1.68
903.1654	-0.2	0.1075	0.0152	4.32	1.70
903.1654	0	0.0000	1.0000	3.55	1.71
903.1654	0.2	0.1094	0.0142	2.84	1.71
903.1654	0.4	0.4708	0.0000	2.21	1.71
911.3666	-0.2	0.3344	0.0000	3.57	1.70
911.3666	0	0.0000	1.0000	3.55	1.68
911.3666	0.2	0.3344	0.0000	3.57	1.69
924.0638	-0.4	0.3544	0.0000	3.53	1.71
924.0638	-0.2	0.0861	0.0350	3.54	1.72
924.0638	0	0.0000	1.0000	3.55	1.73
924.0638	0.2	0.0862	0.0350	3.55	1.70
924.0638	0.4	0.3552	0.0000	3.55	1.71
924.1102	-0.4	0.3560	0.0000	3.55	1.70
924.1102	-0.2	0.0862	0.0349	3.55	1.70
924.1102	0	0.0000	1.0000	3.55	1.71
924.1102	0.2	0.0862	0.0349	3.55	1.71
924.1102	0.4	0.3561	0.0000	3.55	1.71
924.4792	-0.4	0.3530	0.0000	3.47	1.71
924.4792	-0.2	0.0855	0.0359	3.51	1.71
924.4792	0	0.0000	1.0000	3.55	1.71
924.4792	0.2	0.0855	0.0359	3.59	1.71
924.4792	0.4	0.3532	0.0000	3.63	1.71
924.4917	-0.4	0.3543	0.0000	3.56	1.71
924.4917	-0.2	0.0856	0.0358	3.56	1.71
924.4917	0	0.0000	1.0000	3.55	1.71
924.4917	0.2	0.0855	0.0359	3.54	1.71
924.4917	0.4	0.3538	0.0000	3.54	1.71
937.8161	-0.4	0.7955	0.0000	3.70	1.71
937.8161	-0.2	0.1973	0.0005	3.65	1.71
937.8161	0	0.0000	1.0000	3.55	1.71
937.8161	0.2	0.1973	0.0005	3.41	1.71
937.8161	0.4	0.7951	0.0000	3.20	1.71
937.8625	-0.2	0.2006	0.0004	4.45	1.71
937.8625	0	0.0000	1.0000	3.55	1.70
937.8625	0.2	0.2006	0.0004	2.67	1.71
943.8823	-0.4	0.5282	0.0000	2.28	1.71

943.8823	-0.2	0.1296	0.0065	2.92	1.70
943.8823	0	0.0000	1.0000	3.55	1.69
943.8823	0.2	0.1294	0.0065	4.17	1.72
943.8823	0.4	0.5286	0.0000	4.80	1.71
944.4173	-0.2	0.2073	0.0003	3.59	1.69
944.4173	0	0.0000	1.0000	3.55	1.70
944.4173	0.2	0.2060	0.0003	3.57	1.71
946.1963	-0.4	0.4180	0.0000	3.56	1.71
946.1963	-0.2	0.1011	0.0195	3.55	1.70
946.1963	0	0.0000	1.0000	3.55	1.68
946.1963	0.2	0.1011	0.0195	3.55	1.71
946.1963	0.4	0.4183	0.0000	3.56	1.71
956.4962	-0.2	0.2718	0.0000	3.52	1.69
956.4962	0	0.0000	1.0000	3.55	1.70
956.4962	0.2	0.2718	0.0000	3.52	1.70
960.1638	-0.4	0.3648	0.0000	3.89	1.71
960.1638	-0.2	0.0881	0.0325	3.72	1.70
960.1638	0	0.0000	1.0000	3.55	1.70
960.1638	0.2	0.0880	0.0326	3.37	1.70
960.1638	0.4	0.3639	0.0000	3.20	1.71
960.4996	-0.4	0.3689	0.0000	3.55	1.70
960.4996	-0.2	0.0888	0.0316	3.55	1.71
960.4996	0	0.0000	1.0000	3.55	1.71
960.4996	0.2	0.0888	0.0316	3.55	1.71
960.4996	0.4	0.3692	0.0000	3.55	1.71
961.0405	-0.4	0.3655	0.0000	3.71	1.71
961.0405	-0.2	0.0855	0.0359	3.63	1.71
961.0405	0	0.0000	1.0000	3.55	1.71
961.0405	0.2	0.0857	0.0356	3.47	1.71
961.0405	0.4	0.3669	0.0000	3.39	1.71
961.0542	-0.4	0.3675	0.0000	3.45	1.71
961.0542	-0.2	0.0858	0.0354	3.50	1.71
961.0542	0	0.0000	1.0000	3.55	1.71
961.0542	0.2	0.0856	0.0358	3.60	1.71
961.0542	0.4	0.3660	0.0000	3.65	1.71
962.5424	-0.4	0.6486	0.0000	2.96	1.71
962.5424	-0.2	0.1610	0.0019	3.24	1.71
962.5424	0	0.0000	1.0000	3.55	1.71
962.5424	0.2	0.1610	0.0019	3.89	1.71

962.5424	0.4	0.6487	0.0000	4.29	1.71
967.1625	-0.4	0.3900	0.0000	3.54	1.71
967.1625	-0.2	0.0950	0.0248	3.55	1.69
967.1625	0	0.0000	1.0000	3.55	1.70
967.1625	0.2	0.0950	0.0248	3.55	1.71
967.1625	0.4	0.3901	0.0000	3.55	1.70
969.7952	-0.4	0.5366	0.0000	5.10	1.68
969.7952	-0.2	0.1320	0.0059	4.30	1.70
969.7952	0	0.0000	1.0000	3.55	1.70
969.7952	0.2	0.1320	0.0059	2.84	1.71
969.7952	0.4	0.5369	0.0000	2.16	1.71
985.747	-0.4	0.3702	0.0000	3.11	1.70
985.747	-0.2	0.0902	0.0299	3.35	1.70
985.747	0	0.0000	1.0000	3.55	1.71
985.747	0.2	0.0910	0.0290	3.72	1.71
985.747	0.4	0.3765	0.0000	3.86	1.70
987.3214	-0.4	0.3810	0.0000	3.46	1.69
987.3214	-0.2	0.0927	0.0271	3.53	1.69
987.3214	0	0.0000	1.0000	3.55	1.70
987.3214	0.2	0.0927	0.0271	3.54	1.71
987.3214	0.4	0.3818	0.0000	3.48	1.71
1046.116	-0.2	0.2797	0.0000	3.56	1.71
1046.116	0	0.0000	1.0000	3.55	1.70
1046.116	0.2	0.2727	0.0000	3.50	1.71
1047.471	-0.2	0.2780	0.0000	3.56	1.71
1047.471	0	0.0000	1.0000	3.55	1.71
1047.471	0.2	0.2781	0.0000	3.56	1.70
1071.237	-0.2	0.2196	0.0002	3.71	1.67
1071.237	0	0.0000	1.0000	3.55	1.71
1071.237	0.2	0.2120	0.0003	3.38	1.74
1072.294	-0.4	0.7471	0.0000	3.56	1.71
1072.294	-0.2	0.1862	0.0007	3.56	1.71
1072.294	0	0.0000	1.0000	3.55	1.71
1072.294	0.2	0.1862	0.0007	3.56	1.66
1072.294	0.4	0.7479	0.0000	3.57	1.71
1073.731	-0.2	0.2087	0.0003	3.31	1.75
1073.731	0	0.0000	1.0000	3.55	1.71
1073.731	0.2	0.2115	0.0003	3.78	1.71
1077.872	-0.4	0.6422	0.0000	3.63	1.71

1077.872	-0.2	0.1591	0.0020	3.59	1.71
1077.872	0	0.0000	1.0000	3.55	1.71
1077.872	0.2	0.1581	0.0021	3.52	1.76
1077.872	0.4	0.6344	0.0000	3.48	1.71
1077.896	-0.4	0.6431	0.0000	3.73	1.65
1077.896	-0.2	0.1592	0.0020	3.60	1.71
1077.896	0	0.0000	1.0000	3.55	1.71
1077.896	0.2	0.1580	0.0021	3.46	1.71
1077.896	0.4	0.6334	0.0000	3.38	1.71
1082.314	-0.4	0.7503	0.0000	3.55	1.70
1082.314	-0.2	0.1864	0.0007	3.55	1.71
1082.314	0	0.0000	1.0000	3.55	1.71
1082.314	0.2	0.1863	0.0007	3.55	1.71
1082.314	0.4	0.7500	0.0000	3.55	1.70
1106.759	-0.2	0.2753	0.0000	2.84	1.70
1106.759	0	0.0000	1.0000	3.55	1.70
1106.759	0.2	0.2633	0.0000	4.26	1.71
1124.984	-0.2	0.2273	0.0001	3.31	1.71
1124.984	0	0.0000	1.0000	3.55	1.71
1124.984	0.2	0.2275	0.0001	3.79	1.70
1125.518	-0.4	0.7937	0.0000	3.56	1.81
1125.518	-0.2	0.1979	0.0005	3.55	1.71
1125.518	0	0.0000	1.0000	3.55	1.58
1125.518	0.2	0.1979	0.0005	3.56	1.77
1125.518	0.4	0.7937	0.0000	3.58	1.71
1125.653	-0.4	0.8013	0.0000	3.83	1.65
1125.653	-0.2	0.1999	0.0004	3.69	1.71
1125.653	0	0.0000	1.0000	3.55	1.71
1125.653	0.2	0.2000	0.0004	3.41	1.71
1125.653	0.4	0.8017	0.0000	3.29	1.71
1128.664	-0.2	0.2081	0.0003	3.55	1.70
1128.664	0	0.0000	1.0000	3.55	1.67
1128.664	0.2	0.2081	0.0003	3.55	1.69
1147.017	-0.4	0.6925	0.0000	3.58	1.71
1147.017	-0.2	0.1727	0.0012	3.57	1.73
1147.017	0	0.0000	1.0000	3.55	1.75
1147.017	0.2	0.1727	0.0012	3.57	1.71
1147.017	0.4	0.6925	0.0000	3.58	1.71
1149.335	-0.4	0.6361	0.0000	3.89	1.71

1149.335	-0.2	0.1588	0.0021	3.72	1.70
1149.335	0	0.0000	1.0000	3.55	1.71
1149.335	0.2	0.1595	0.0020	3.38	1.71
1149.335	0.4	0.6419	0.0000	3.21	1.71
1156.059	-0.4	0.6211	0.0000	3.59	1.70
1156.059	-0.2	0.1540	0.0025	3.56	1.68
1156.059	0	0.0000	1.0000	3.55	1.70
1156.059	0.2	0.1541	0.0025	3.56	1.71
1156.059	0.4	0.6213	0.0000	3.59	1.72
1164.553	-0.4	0.5691	0.0000	3.85	1.73
1164.553	-0.2	0.1390	0.0045	3.71	1.69
1164.553	0	0.0000	1.0000	3.55	1.70
1164.553	0.2	0.1359	0.0051	3.38	1.71
1164.553	0.4	0.5426	0.0000	3.20	1.70
1189.618	-0.4	0.5211	0.0000	3.57	1.69
1189.618	-0.2	0.1290	0.0066	3.56	1.61
1189.618	0	0.0000	1.0000	3.55	1.66
1189.618	0.2	0.1290	0.0066	3.56	1.71
1189.618	0.4	0.5210	0.0000	3.57	1.74
1189.864	-0.4	0.4742	0.0000	3.61	1.77
1189.864	-0.2	0.1177	0.0102	3.58	1.70
1189.864	0	0.0000	1.0000	3.55	1.71
1189.864	0.2	0.1179	0.0102	3.52	1.71
1189.864	0.4	0.4757	0.0000	3.49	1.71
1190.034	-0.4	0.5067	0.0000	3.56	1.70
1190.034	-0.2	0.1252	0.0077	3.56	1.70
1190.034	0	0.0000	1.0000	3.55	1.71
1190.034	0.2	0.1252	0.0076	3.56	1.71
1190.034	0.4	0.5068	0.0000	3.56	1.71
1190.67	-0.4	0.5392	0.0000	3.30	1.71
1190.67	-0.2	0.1340	0.0054	3.42	1.71
1190.67	0	0.0000	1.0000	3.55	1.71
1190.67	0.2	0.1339	0.0054	3.68	1.71
1190.67	0.4	0.5378	0.0000	3.80	1.71
1194.926	-0.4	0.4818	0.0000	3.54	1.71
1194.926	-0.2	0.1192	0.0097	3.55	1.71
1194.926	0	0.0000	1.0000	3.55	1.71
1194.926	0.2	0.1192	0.0096	3.56	1.71
1194.926	0.4	0.4818	0.0000	3.55	1.70

1195.936	-0.4	0.4860	0.0000	3.72	1.70
1195.936	-0.2	0.1206	0.0091	3.64	1.70
1195.936	0	0.0000	1.0000	3.55	1.71
1195.936	0.2	0.1209	0.0090	3.45	1.71
1195.936	0.4	0.4882	0.0000	3.35	1.71
1204.498	-0.4	0.7731	0.0000	3.54	1.70
1204.498	-0.2	0.1926	0.0006	3.56	1.67
1204.498	0	0.0000	1.0000	3.55	1.69
1204.498	0.2	0.1926	0.0006	3.55	1.71
1204.498	0.4	0.7732	0.0000	3.53	1.72
1206.754	-0.2	0.2070	0.0003	3.23	1.73
1206.754	0	0.0000	1.0000	3.55	1.71
1206.754	0.2	0.2059	0.0003	3.87	1.71
1221.579	-0.2	0.2561	0.0000	3.45	1.71
1221.579	0	0.0000	1.0000	3.55	1.71
1221.579	0.2	0.2561	0.0000	3.45	1.71
1237.167	-0.2	0.2699	0.0000	3.58	1.73
1237.167	0	0.0000	1.0000	3.55	1.71
1237.167	0.2	0.2673	0.0000	3.42	1.68
1238.566	-0.2	0.3707	0.0000	3.56	1.69
1238.566	0	0.0000	1.0000	3.55	1.71
1238.566	0.2	0.3709	0.0000	3.56	1.69
1239.416	-0.2	0.2432	0.0001	3.55	1.69
1239.416	0	0.0000	1.0000	3.55	1.71
1239.416	0.2	0.2434	0.0001	3.55	1.70
1239.82	-0.2	0.2758	0.0000	3.53	1.71
1239.82	0	0.0000	1.0000	3.55	1.71
1239.82	0.2	0.2863	0.0000	3.56	1.71
1240.694	-0.2	0.2902	0.0000	3.93	1.71
1240.694	0	0.0000	1.0000	3.55	1.71
1240.694	0.2	0.2834	0.0000	3.13	1.71
1246.425	-0.2	0.2741	0.0000	3.60	1.72
1246.425	0	0.0000	1.0000	3.55	1.71
1246.425	0.2	0.2742	0.0000	3.60	1.70
1272.286	-0.2	0.2888	0.0000	3.61	1.68
1272.286	0	0.0000	1.0000	3.55	1.71
1272.286	0.2	0.2776	0.0000	3.27	1.73
1295.17	-0.2	0.3485	0.0000	3.49	1.70
1295.17	0	0.0000	1.0000	3.55	1.71

1295.17	0.2	0.3484	0.0000	3.49	1.70
1297.703	-0.2	0.2725	0.0000	3.24	1.67
1297.703	0	0.0000	1.0000	3.55	1.71
1297.703	0.2	0.2733	0.0000	3.77	1.72
1302.991	-0.2	0.4100	0.0000	3.56	1.70
1302.991	0	0.0000	1.0000	3.55	1.71
1302.991	0.2	0.4100	0.0000	3.56	1.70
1309.166	-0.2	0.2683	0.0000	3.67	1.70
1309.166	0	0.0000	1.0000	3.55	1.71
1309.166	0.2	0.2687	0.0000	3.43	1.72
1314.227	-0.2	0.2872	0.0000	3.57	1.70
1314.227	0	0.0000	1.0000	3.55	1.71
1314.227	0.2	0.2872	0.0000	3.57	1.70
1316.863	-0.2	0.2973	0.0000	3.58	1.66
1316.863	0	0.0000	1.0000	3.55	1.71
1316.863	0.2	0.2958	0.0000	3.51	1.75
1320.34	-0.2	0.2849	0.0000	3.48	1.70
1320.34	0	0.0000	1.0000	3.55	1.71
1320.34	0.2	0.2819	0.0000	3.54	1.70
1320.432	-0.2	0.4503	0.0000	3.68	1.67
1320.432	0	0.0000	1.0000	3.55	1.71
1320.432	0.2	0.4500	0.0000	3.67	1.73
1323.458	-0.2	0.2249	0.0002	3.55	1.63
1323.458	0	0.0000	1.0000	3.55	1.71
1323.458	0.2	0.2249	0.0002	3.55	1.79
1331.409	-0.2	0.2504	0.0001	3.55	1.68
1331.409	0	0.0000	1.0000	3.55	1.71
1331.409	0.2	0.2503	0.0001	3.55	1.68
1332.83	-0.2	0.5847	0.0000	3.77	1.71
1332.83	0	0.0000	1.0000	3.55	1.71
1332.83	0.2	0.5799	0.0000	3.66	1.71
1342.591	-0.2	0.8394	0.0000	3.52	1.70
1342.591	0	0.0000	1.0000	3.55	1.71
1342.591	0.2	0.8424	0.0000	3.51	1.70
1342.656	-0.2	0.8549	0.0000	3.54	1.94
1342.656	0	0.0000	1.0000	3.55	1.71
1342.656	0.2	0.8571	0.0000	3.51	1.45
1355.59	-0.2	0.5734	0.0000	3.44	1.72
1355.59	0	0.0000	1.0000	3.55	1.71

1355.59	0.2	0.5733	0.0000	3.43	1.70
1358.664	-0.2	0.4136	0.0000	3.72	1.73
1358.664	0	0.0000	1.0000	3.55	1.71
1358.664	0.2	0.4148	0.0000	3.28	1.68
1360.865	-0.2	0.4345	0.0000	3.61	1.71
1360.865	0	0.0000	1.0000	3.55	1.71
1360.865	0.2	0.4345	0.0000	3.62	1.71
1364.508	-0.2	0.4665	0.0000	3.07	1.76
1364.508	0	0.0000	1.0000	3.55	1.71
1364.508	0.2	0.4934	0.0000	3.87	1.66
1365.294	-0.2	0.5380	0.0000	3.51	1.71
1365.294	0	0.0000	1.0000	3.55	1.71
1365.294	0.2	0.5353	0.0000	3.48	1.71
1371.759	-0.2	0.9470	0.0000	3.48	1.70
1371.759	0	0.0000	1.0000	3.55	1.71
1371.759	0.2	0.8924	0.0000	3.63	1.70
1372.228	-0.2	0.8662	0.0000	3.56	1.70
1372.228	0	0.0000	1.0000	3.55	1.71
1372.228	0.2	0.8736	0.0000	3.55	1.70
1378.226	-0.2	0.6946	0.0000	3.73	1.79
1378.226	0	0.0000	1.0000	3.55	1.71
1378.226	0.2	0.7101	0.0000	3.44	1.63
1401.722	-0.2	0.4578	0.0000	2.82	1.70
1401.722	0	0.0000	1.0000	3.55	1.71
1401.722	0.2	0.4676	0.0000	3.86	1.71
1448.159	-0.2	0.4873	0.0000	3.55	1.58
1448.159	0	0.0000	1.0000	3.55	1.71
1448.159	0.2	0.4872	0.0000	3.55	1.75
1457.014	-0.2	0.6338	0.0000	3.51	1.97
1457.014	0	0.0000	1.0000	3.55	1.71
1457.014	0.2	0.6092	0.0000	3.53	1.47
1476.637	-0.2	0.4348	0.0000	3.63	1.70
1476.637	0	0.0000	1.0000	3.55	1.71
1476.637	0.2	0.4376	0.0000	3.31	1.70
1484.274	-0.2	0.4350	0.0000	3.51	1.58
1484.274	0	0.0000	1.0000	3.55	1.71
1484.274	0.2	0.4350	0.0000	3.51	1.84
1487.258	-0.2	0.3827	0.0000	3.38	1.74
1487.258	0	0.0000	1.0000	3.55	1.71

1487.258	0.2	0.3843	0.0000	3.62	1.65
1493.029	-0.2	0.4048	0.0000	3.63	1.71
1493.029	0	0.0000	1.0000	3.55	1.71
1493.029	0.2	0.4049	0.0000	3.48	1.71
1493.367	-0.2	0.4108	0.0000	3.55	1.68
1493.367	0	0.0000	1.0000	3.55	1.71
1493.367	0.2	0.4108	0.0000	3.55	1.72
1504.901	-0.2	0.4617	0.0000	3.54	1.72
1504.901	0	0.0000	1.0000	3.55	1.71
1504.901	0.2	0.4617	0.0000	3.54	1.70
1511.544	-0.2	0.4466	0.0000	3.56	1.71
1511.544	0	0.0000	1.0000	3.55	1.71
1511.544	0.2	0.4466	0.0000	3.56	1.71
1512.476	-0.2	0.4409	0.0000	3.52	1.71
1512.476	0	0.0000	1.0000	3.55	1.71
1512.476	0.2	0.4406	0.0000	3.57	1.71
1517.697	-0.2	0.4795	0.0000	3.56	1.71
1517.697	0	0.0000	1.0000	3.55	1.71
1517.697	0.2	0.4795	0.0000	3.56	1.71
1528.296	-0.2	0.5022	0.0000	4.11	1.71
1528.296	0	0.0000	1.0000	3.55	1.71
1528.296	0.2	0.5139	0.0000	3.05	1.71
1533.451	-0.2	0.5048	0.0000	3.52	1.71
1533.451	0	0.0000	1.0000	3.55	1.71
1533.451	0.2	0.5049	0.0000	3.51	1.71
1533.684	-0.2	0.5044	0.0000	3.51	1.58
1533.684	0	0.0000	1.0000	3.55	1.71
1533.684	0.2	0.5044	0.0000	3.51	1.84
1552.447	-0.2	0.7145	0.0000	3.60	1.70
1552.447	0	0.0000	1.0000	3.55	1.71
1552.447	0.2	0.7142	0.0000	3.56	1.70
1552.822	-0.2	0.5430	0.0000	4.19	1.69
1552.822	0	0.0000	1.0000	3.55	1.71
1552.822	0.2	0.5407	0.0000	3.03	1.71
1555.253	-0.2	0.5611	0.0000	3.56	1.70
1555.253	0	0.0000	1.0000	3.55	1.71
1555.253	0.2	0.5611	0.0000	3.56	1.70
1560.914	-0.2	0.8016	0.0000	3.59	1.73
1560.914	0	0.0000	1.0000	3.55	1.71

1560.914	0.2	0.7855	0.0000	3.69	1.67
1606.184	-0.2	0.8809	0.0000	3.47	1.70
1606.184	0	0.0000	1.0000	3.55	1.71
1606.184	0.2	0.8798	0.0000	3.47	1.70
1607.403	-0.2	0.8737	0.0000	3.42	1.53
1607.403	0	0.0000	1.0000	3.55	1.71
1607.403	0.2	0.9763	0.0000	3.46	1.87
1608.294	-0.2	0.8786	0.0000	3.50	1.70
1608.294	0	0.0000	1.0000	3.55	1.71
1608.294	0.2	0.8780	0.0000	3.50	1.70
1613.161	-0.2	0.9533	0.0000	3.15	1.80
1613.161	0	0.0000	1.0000	3.55	1.71
1613.161	0.2	1.0102	0.0000	3.74	1.60
1624.514	-0.2	1.1107	0.0000	3.67	1.68
1624.514	0	0.0000	1.0000	3.55	1.71
1624.514	0.2	1.1133	0.0000	3.48	1.68
1624.547	-0.2	1.1134	0.0000	3.47	1.78
1624.547	0	0.0000	1.0000	3.55	1.71
1624.547	0.2	1.1108	0.0000	3.68	1.58
1631.054	-0.2	1.0281	0.0000	3.50	1.70
1631.054	0	0.0000	1.0000	3.55	1.71
1631.054	0.2	1.0282	0.0000	3.50	1.71
1635.247	-0.2	0.9979	0.0000	2.77	1.71
1635.247	0	0.0000	1.0000	3.55	1.71
1635.247	0.2	0.9803	0.0000	4.37	1.70
1641.934	-0.2	1.2379	0.0000	3.60	1.68
1641.934	0	0.0000	1.0000	3.55	1.71
1641.934	0.2	1.2404	0.0000	3.52	1.68
1641.975	-0.2	1.2408	0.0000	3.48	1.43
1641.975	0	0.0000	1.0000	3.55	1.71
1641.975	0.2	1.2379	0.0000	3.65	1.96
1657.493	-0.2	1.4674	0.0000	3.56	1.70
1657.493	0	0.0000	1.0000	3.55	1.71
1657.493	0.2	1.4655	0.0000	3.58	1.70
1658.284	-0.2	1.4549	0.0000	3.95	1.70
1658.284	0	0.0000	1.0000	3.55	1.71
1658.284	0.2	1.4785	0.0000	3.18	1.70
1678.281	-0.2	1.4959	0.0000	3.77	1.70
1678.281	0	0.0000	1.0000	3.55	1.71

1678.281	0.2	1.3504	0.0000	3.39	1.70
1678.479	-0.2	1.4103	0.0000	3.56	1.67
1678.479	0	0.0000	1.0000	3.55	1.71
1678.479	0.2	1.4429	0.0000	3.59	1.74
3187.499	-0.2	0.8714	0.0000	3.65	1.64
3187.499	0	0.0000	1.0000	3.55	1.71
3187.499	0.2	0.7906	0.0000	3.45	1.76
3187.616	-0.2	0.8205	0.0000	3.54	1.71
3187.616	0	0.0000	1.0000	3.55	1.71
3187.616	0.2	0.8421	0.0000	3.56	1.70
3195.262	-0.2	0.7794	0.0000	3.56	1.71
3195.262	0	0.0000	1.0000	3.55	1.71
3195.262	0.2	0.9300	0.0000	3.54	1.70
3195.276	-0.2	0.7786	0.0000	3.56	1.71
3195.276	0	0.0000	1.0000	3.55	1.71
3195.276	0.2	0.9306	0.0000	3.54	1.71
3195.744	-0.2	0.8865	0.0000	3.55	1.71
3195.744	0	0.0000	1.0000	3.55	1.71
3195.744	0.2	0.8060	0.0000	3.55	1.71
3195.771	-0.2	0.8004	0.0000	3.54	1.71
3195.771	0	0.0000	1.0000	3.55	1.71
3195.771	0.2	0.8918	0.0000	3.56	1.71
3199.066	-0.2	0.8239	0.0000	3.52	1.71
3199.066	0	0.0000	1.0000	3.55	1.71
3199.066	0.2	0.8519	0.0000	3.58	1.71
3199.096	-0.2	0.8467	0.0000	3.55	1.71
3199.096	0	0.0000	1.0000	3.55	1.71
3199.096	0.2	0.8291	0.0000	3.54	1.71
3204.29	-0.2	0.6295	0.0000	3.54	1.70
3204.29	0	0.0000	1.0000	3.55	1.71
3204.29	0.2	1.1598	0.0000	3.57	1.71
3204.375	-0.2	1.1605	0.0000	3.57	1.71
3204.375	0	0.0000	1.0000	3.55	1.71
3204.375	0.2	0.6284	0.0000	3.54	1.71
3208.807	-0.2	0.8312	0.0000	3.56	1.70
3208.807	0	0.0000	1.0000	3.55	1.71
3208.807	0.2	0.8771	0.0000	3.54	1.71
3208.82	-0.2	0.8783	0.0000	3.53	1.71
3208.82	0	0.0000	1.0000	3.55	1.71

3208.82	0.2	0.8296	0.0000	3.57	1.70
3209.184	-0.2	0.8296	0.0000	3.55	1.71
3209.184	0	0.0000	1.0000	3.55	1.71
3209.184	0.2	0.8546	0.0000	3.55	1.71
3209.198	-0.2	0.8717	0.0000	3.54	1.71
3209.198	0	0.0000	1.0000	3.55	1.71
3209.198	0.2	0.8119	0.0000	3.56	1.71
3210.236	-0.2	0.8840	0.0000	3.54	1.71
3210.236	0	0.0000	1.0000	3.55	1.71
3210.236	0.2	0.8000	0.0000	3.55	1.71
3210.364	-0.2	0.8372	0.0000	3.55	1.71
3210.364	0	0.0000	1.0000	3.55	1.71
3210.364	0.2	0.8475	0.0000	3.55	1.71
3218.991	-0.2	0.8429	0.0000	3.55	1.71
3218.991	0	0.0000	1.0000	3.55	1.71
3218.991	0.2	0.8477	0.0000	3.55	1.71
3219.385	-0.2	0.8584	0.0000	3.52	1.71
3219.385	0	0.0000	1.0000	3.55	1.71
3219.385	0.2	0.8357	0.0000	3.57	1.71
3220.738	-0.2	0.9491	0.0000	3.54	1.71
3220.738	0	0.0000	1.0000	3.55	1.71
3220.738	0.2	0.7603	0.0000	3.56	1.71
3220.773	-0.2	0.7630	0.0000	3.56	1.70
3220.773	0	0.0000	1.0000	3.55	1.71
3220.773	0.2	0.9509	0.0000	3.54	1.71
3220.942	-0.2	0.8868	0.0000	3.56	1.71
3220.942	0	0.0000	1.0000	3.55	1.71
3220.942	0.2	0.8150	0.0000	3.56	1.71
3221.062	-0.2	0.7245	0.0000	3.54	1.71
3221.062	0	0.0000	1.0000	3.55	1.71
3221.062	0.2	0.9756	0.0000	3.55	1.71
3221.187	-0.2	0.7577	0.0000	3.53	1.71
3221.187	0	0.0000	1.0000	3.55	1.71
3221.187	0.2	0.9266	0.0000	3.55	1.71
3221.601	-0.2	0.8430	0.0000	3.55	1.71
3221.601	0	0.0000	1.0000	3.55	1.71
3221.601	0.2	0.8444	0.0000	3.55	1.71
3230.225	-0.2	0.7766	0.0000	3.56	1.70
3230.225	0	0.0000	1.0000	3.55	1.71

3230.225	0.2	0.9635	0.0000	3.52	1.71
3230.343	-0.2	1.0582	0.0000	3.45	1.71
3230.343	0	0.0000	1.0000	3.55	1.71
3230.343	0.2	0.6802	0.0000	3.63	1.71
3235.487	-0.2	1.0672	0.0000	3.59	1.71
3235.487	0	0.0000	1.0000	3.55	1.71
3235.487	0.2	0.7226	0.0000	3.51	1.71
3235.557	-0.2	0.7356	0.0000	3.50	1.71
3235.557	0	0.0000	1.0000	3.55	1.71
3235.557	0.2	1.0506	0.0000	3.59	1.71
3235.874	-0.2	1.1514	0.0000	3.57	1.71
3235.874	0	0.0000	1.0000	3.55	1.71
3235.874	0.2	0.6389	0.0000	3.54	1.71
3235.935	-0.2	0.6410	0.0000	3.54	1.71
3235.935	0	0.0000	1.0000	3.55	1.71
3235.935	0.2	1.1457	0.0000	3.57	1.71

Table S7. Estimated parameters relating the Franck–Condon factor between T_1 and S_0 (FC).

Chromophores	$\sum_p \left\| \frac{\partial \langle T_1 \| \hat{H}_{SO} \| S_0 \rangle}{\partial Q_p} \right\|^2 P(\mathrm{RT}) FC$ [au]	$\sum_p \left\| \frac{\partial \langle T_1 \| \hat{H}_{SO} \| S_0 \rangle}{\partial Q_p} \right\|^2 P(\mathrm{RT})$ [au]	η	$(E_{T_1-S_0} - E_0)/\eta$ [b] [10^3 cm^{-1}]	FC [10^{-5} au]
1h	3.8×10^{-5}	5.3×10^{0}	0.381	31.4	7.18
2h	4.4×10^{-5}	6.6×10^{0}	0.377	31.8	6.65
3h	5.7×10^{-4}	8.9×10^{1}	0.375	31.9	6.40

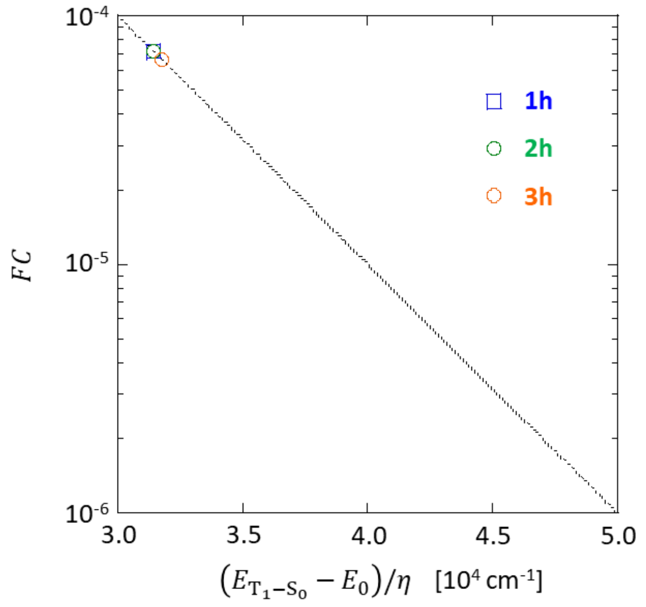

Figure S33. Relationship between FC and $(E_{T_1-S_0} - E_0)/\eta$ for converting from $E_{T_1-S_0}$ and η to FC for protonated chromophores, where E_0 = 4000 cm^{-1} is the empirical parameter by Siebrand. Reference [S12] shows the relationship. η is the ratio of the number of hydrogen atoms to the total number of hydrogen atoms and carbon. η = 0.4 was used for calculating the FC of **3h**. $E_{T_1-S_0}$ = 15,977 cm^{-1}, which is the peak wavelength of phosphorescence of **3h** that was used for calculating the FC of **3h**. Reference [S9] shows information for determining FC for **1h** and **2h**.

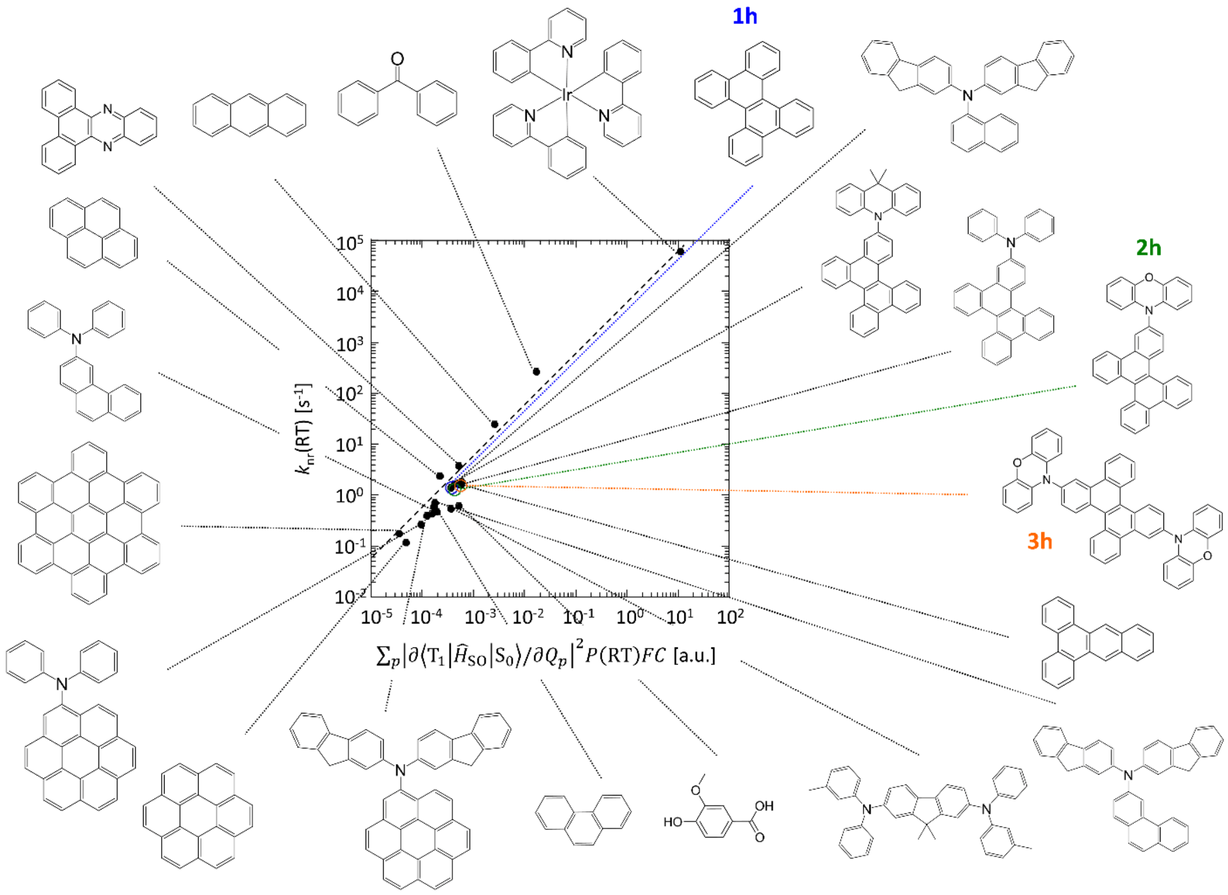

Figure S34. Correlation between optically determined k_{nr}(RT) and $\sum_p |\partial\langle T_1|\hat{H}_{SO}|S_0\rangle/\partial Q_p|^2\, P(\mathrm{RT})FC$ for the chromophores. $\sum_p |\partial\langle T_1|\hat{H}_{SO}|S_0\rangle/\partial Q_p|^2\, P(\mathrm{RT})FC$ of isolated **3h** was determined by the same procedure as in reference S13 to compare $\sum_p |\partial\langle T_1|\hat{H}_{SO}|S_0\rangle/\partial Q_p|^2\, P(\mathrm{RT})FC$ values with previous calculated data (black dots). Reference S9 shows data for **1h** and **2h**. References [S3, S9, S13–S16] summarize the black dots. The dashed line represents a slope of 1.

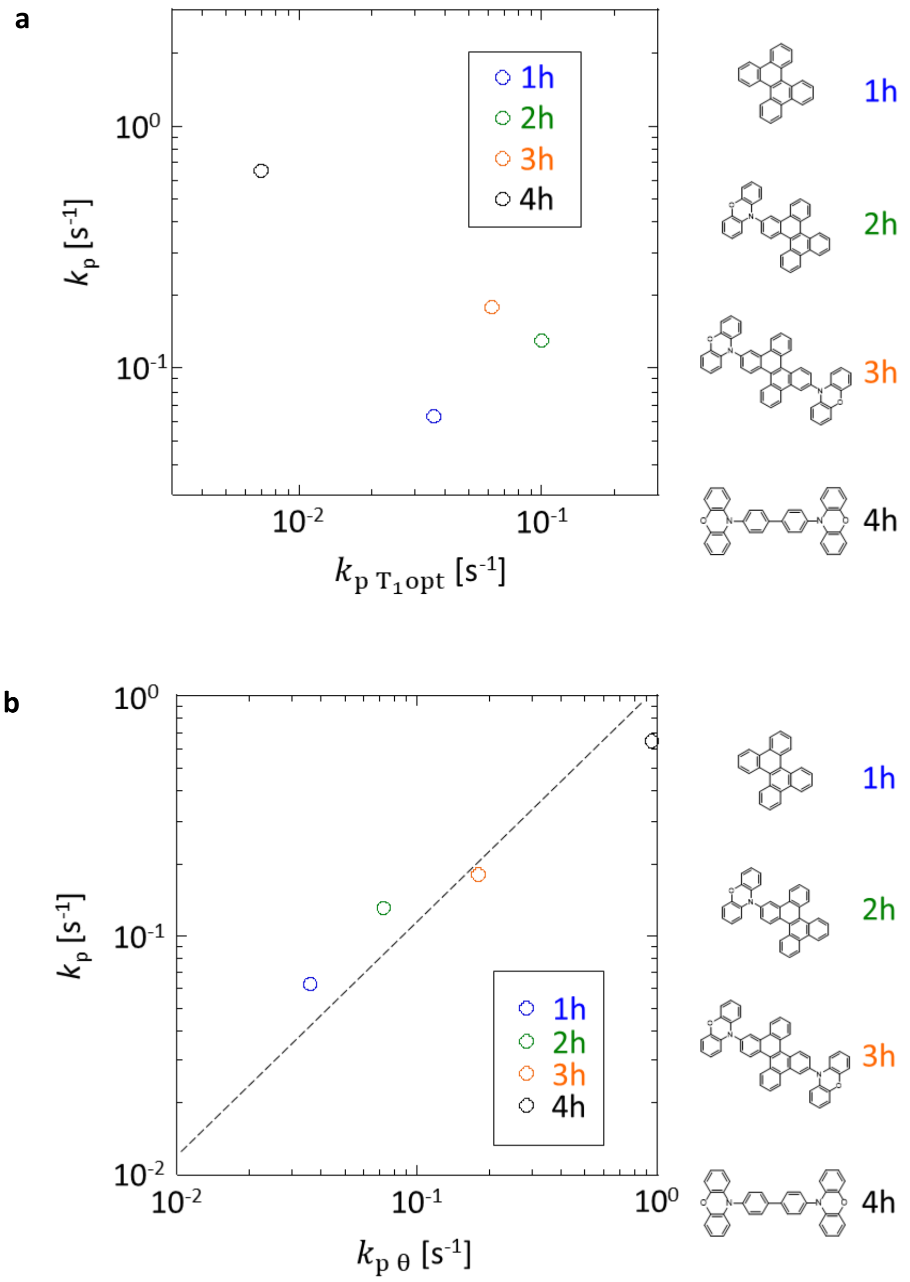

Figure S35. (a) k_{p} vs. $k_{\mathrm{p\ T_1opt}}$ plot and (b) k_{p} vs. $k_{\mathrm{p}\ \theta}$ plot using **1h**–**4h**. k_{p} is data determined by using 0.3 wt% chromophores doped into amorphous *β*-estradiol. $k_{\mathrm{p\ T_1opt}}$ and $k_{\mathrm{p}\ \theta}$ were determined by the procedure used in Figure 3.

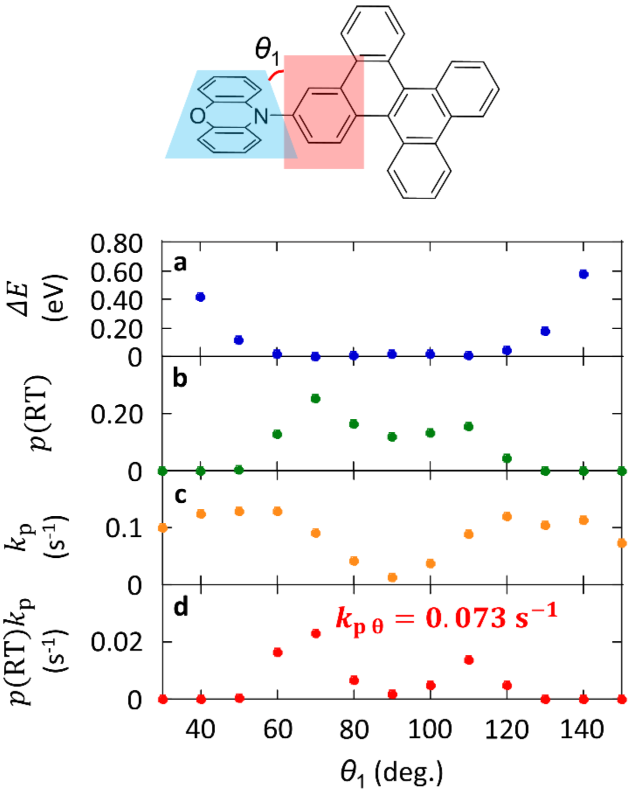

Figure S36. Contribution of the change of θ_1 considering the thermal energy to k_p of **2h**. The change of ΔE (a), p(RT) (b), k_p (c), and p(RT)k_p (d) when θ_1 was independently changed.

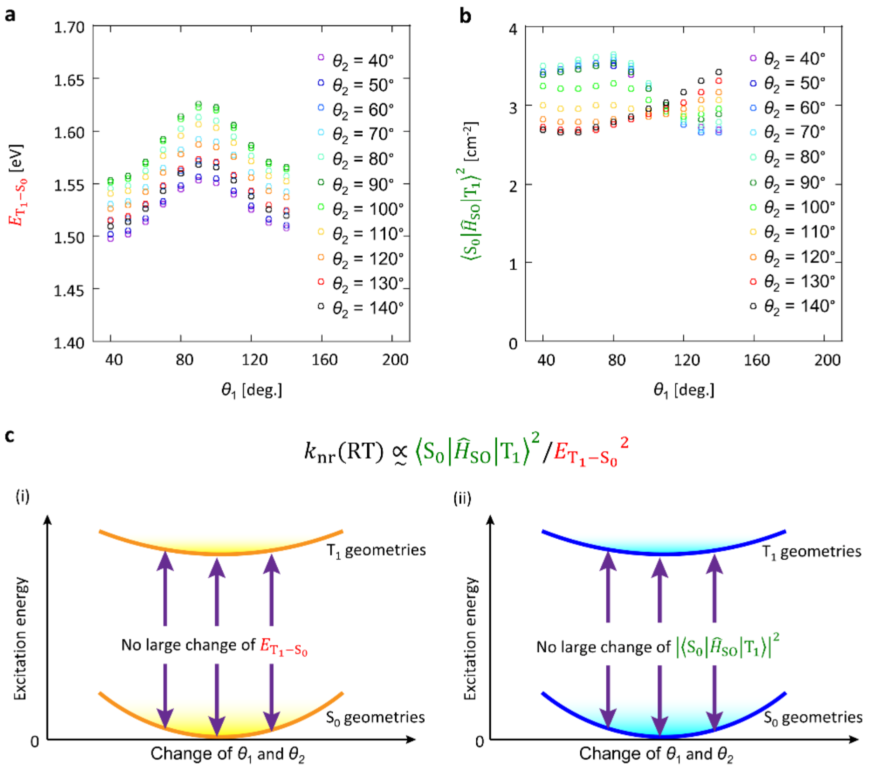

Figure S37. Contribution of the change of θ_1 and θ_2, considering the thermal energy to k_{nr}(RT) of **3h**. (a) Relationship between the T_1–S_0 energy and θ_1 when θ_2 = 70°. (b) Relationship between $\langle S_0|\hat{H}_{SO}|T_1\rangle^2$ and θ_1 when θ_2 = 70°. (c) (i) Visualization of the results of (a) by using a Jablonski diagram. (ii) Visualization of the results of (b) by using a Jablonski diagram.

a

n	S_n-S_0 energy (eV)	T_n-S_0 energy (eV)
1	2.94	1.58
2	3.27	3.00
3	3.59	3.18
4	3.78	3.22
5	3.93	3.26
6	3.96	3.31
7	4.35	3.44
8	4.4	3.53
9	4.48	3.88
10	4.62	3.98

b

n	S_n-S_0 energy (eV)	T_n-S_0 energy (eV)
1	2.33	1.58
2	2.96	2.32
3	3.03	2.83
4	3.29	2.93
5	3.38	3.06
6	3.56	3.18
7	3.65	3.21
8	3.67	3.24
9	3.82	3.30
10	3.88	3.34

c

n	S_n-S_0 energy (eV)	T_n-S_0 energy (eV)
1	2.19	1.55
2	2.28	2.20
3	2.92	2.29
4	3.01	2.77
5	3.01	2.83
6	3.26	2.88
7	3.26	2.96
8	3.39	3.05
9	3.47	3.13
10	3.51	3.18

d

n	S_n-S_0 energy (eV)	T_n-S_0 energy (eV)
1	2.39	1.93
2	2.54	2.36
3	3.42	2.83
4	3.52	2.84
5	3.64	2.84
6	3.67	3.24
7	3.88	3.35
8	4.02	3.42
9	4.04	3.45
10	4.09	3.56

Figure S38. S_n–S_0 transition energies and T_n–S_0 transition energies of **1h–4h**. (a) **1h**. (b) **2h**. (c) **3h**. (d) **4h**.

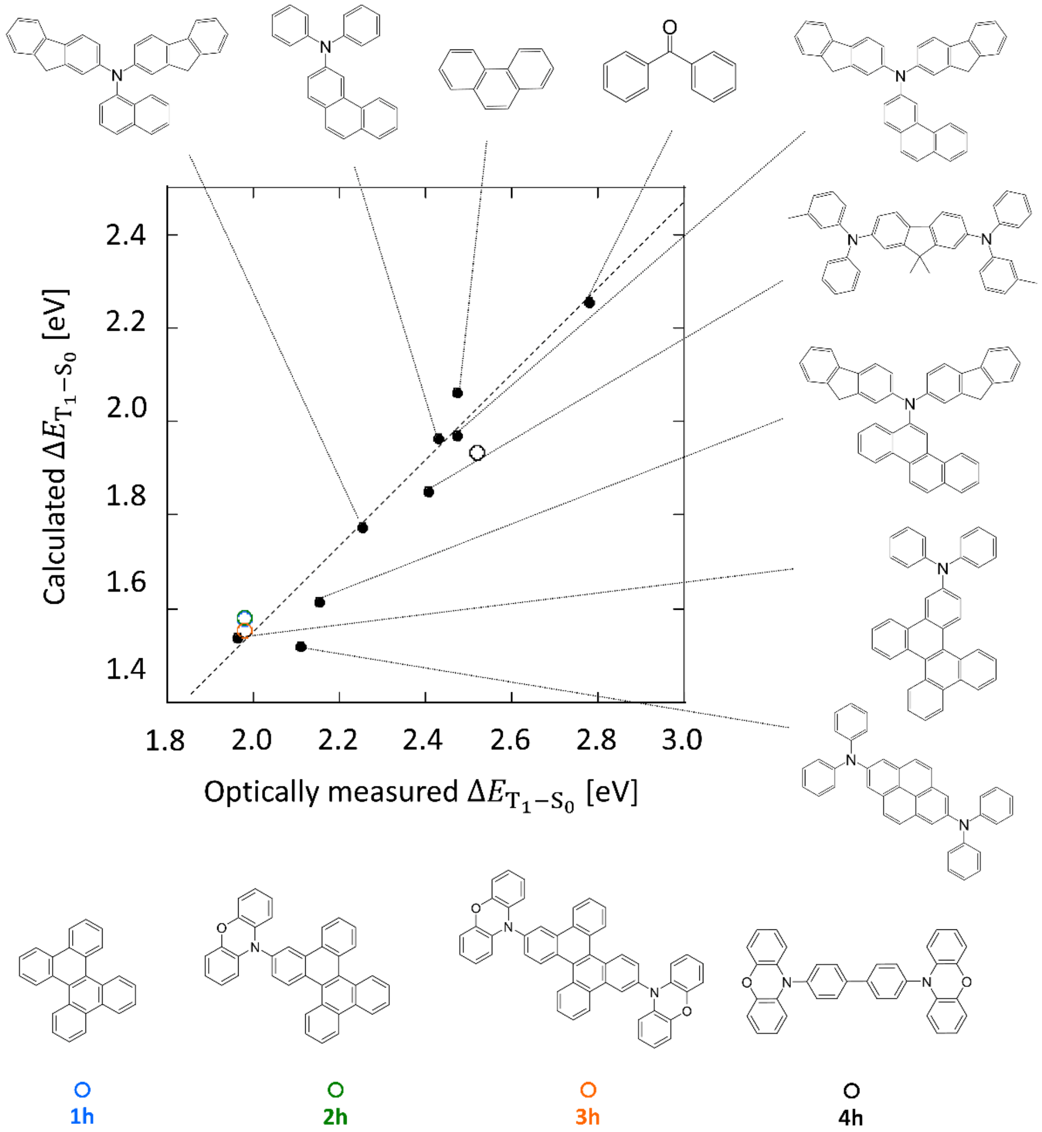

Figure S39. Relationship between the calculated T_1–S_0 energy (*y*-axis) and the peak wavelength of RTP (*x*-axis) for heavy-atom-free chromophores. Figure S21 in reference [4h] show data for the black plots.

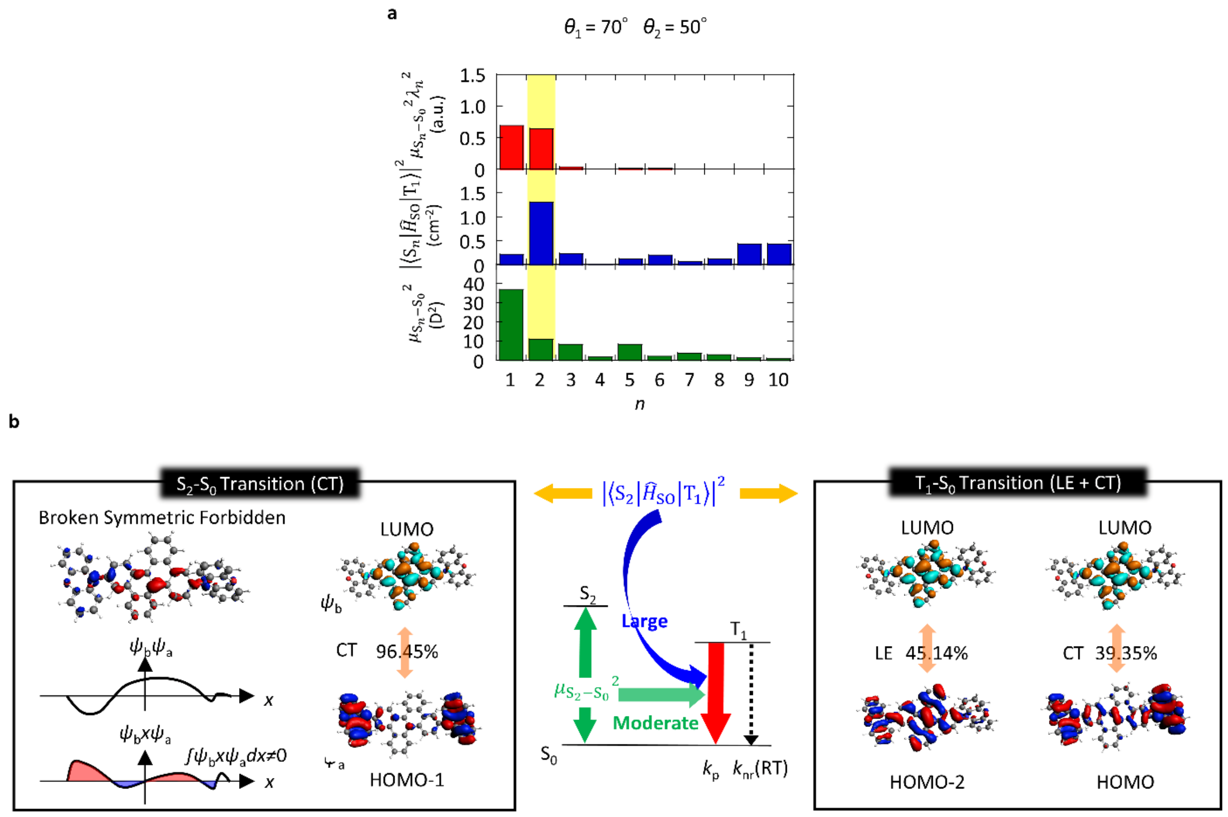

Figure S40. (a) $\mu_{S_n-S_0}{}^2\lambda_n{}^2$ (i), $\langle S_n|\hat{H}_{SO}|T_1\rangle^2$ (ii), and $\mu_{S_n-S_0}{}^2$ (iii) for each n for a geometry with θ_1 = 70° and θ_2 = 50° of **3h**. (b) Schematic of molecular orbitals for k_p enhancement that is attributable to S_2–S_0 transitions for a geometry in which θ_1 = 70° and θ_2 = 50°.

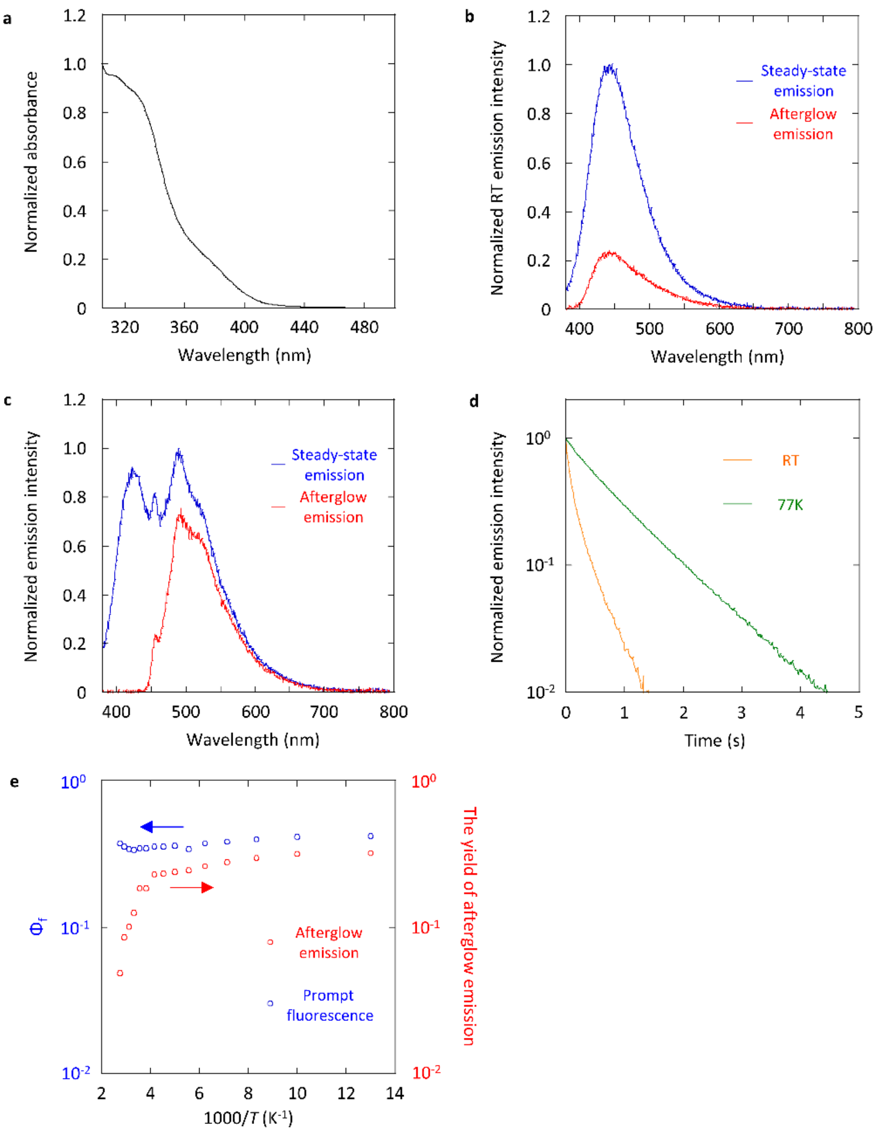

Figure S41. Optical characteristics of 0.3 wt% **4h** doped amorphous *β*-estradiol film. (a) Normalized absorption spectra. (b) Steady-state emission spectrum under excitation (blue) and afterglow emission spectrum after excitation (red) at RT. (c) Steady-state emission spectrum under excitation (blue) and afterglow emission spectra after excitation (red) at 77K. In (b) and (c), excitation < 0.1 mW/cm^2 was used to avoid annihilation of fluorescence and afterglow emission. (d) Afterglow emission decay characteristics at 77K and RT. (e) Temperature dependence of Φ_f (top) and the yield of afterglow emission (bottom). The excitation wavelength was 340 nm. Afterglow emission at RT is mainly caused by thermally activated delayed fluorescence whereas the afterglow emission at 77K is caused by phosphorescence because of single-exponential decay characteristics (Figure S22c). Therefore, Φ_p(77K) = 0.32 was used to determine k_p of **4h** in amorphous *β*-estradiol. Because Φ_{isc} is considered as 1 – Φ_f(RT) from Table S2 and Φ_f(RT) of **4h** is 0.33 in amorphous *β*-estradiol, Φ_{isc} was determined as 0.67. Because τ_p(77K) is 0.78 s, k_p was determined as 0.61 s^{-1} from Φ_p(77K) = $\Phi_{isc}k_p\tau_p$(77K).

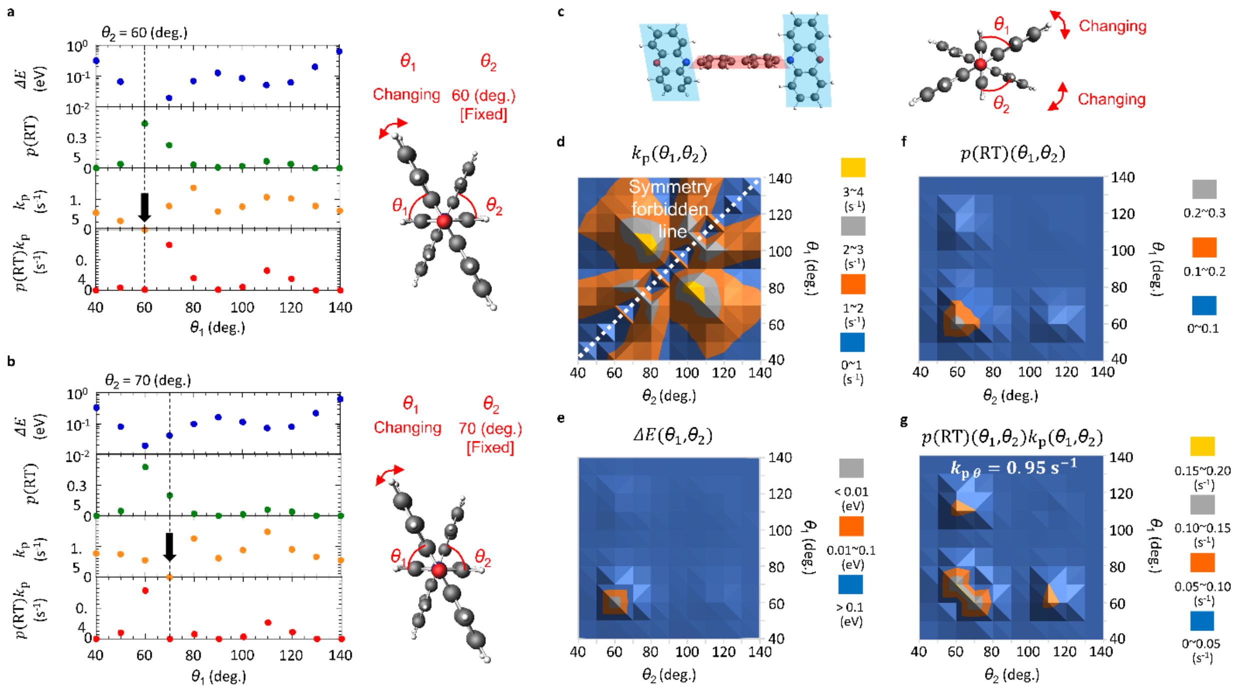

Figure S42. Contribution of the change of θ_1 and θ_2 considering the thermal energy to k_p of **4h**. (a) The change of ΔE, p(RT), k_p, and $p(RT)k_p$ when θ_1 was changed yet keeping θ_2 = 60°. (b) The change of ΔE, p(RT), k_p, and $p(RT)k_p$ when θ_1 was changed yet θ_2 = 70° was kept. (c) Molecular illustration that explains the independent change of θ_1 and θ_2 for **4h**. (d–g) Two-dimensional histograms of k_p (d), ΔE (e), p(RT) (f), and p(RT) k_p (g) when θ_1 and θ_2 were independently changed.

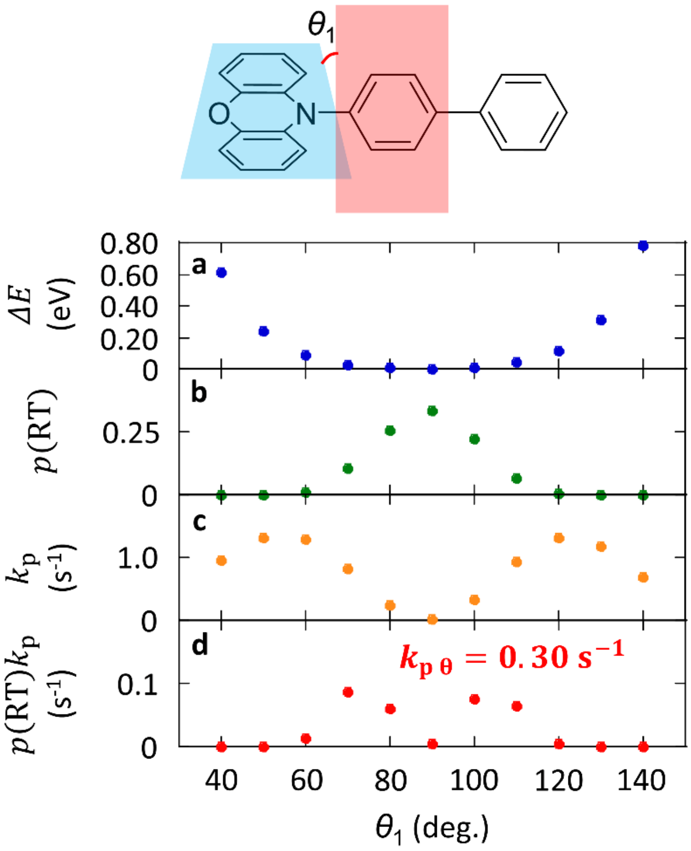

Figure S43. Contribution of the change of θ_1 considering the thermal energy to k_p of biphenyl substituted with a phenoxazine. The change of ΔE (a), p(RT) (b), k_p (c), and p(RT)k_p (d) when θ_1 was independently changed.

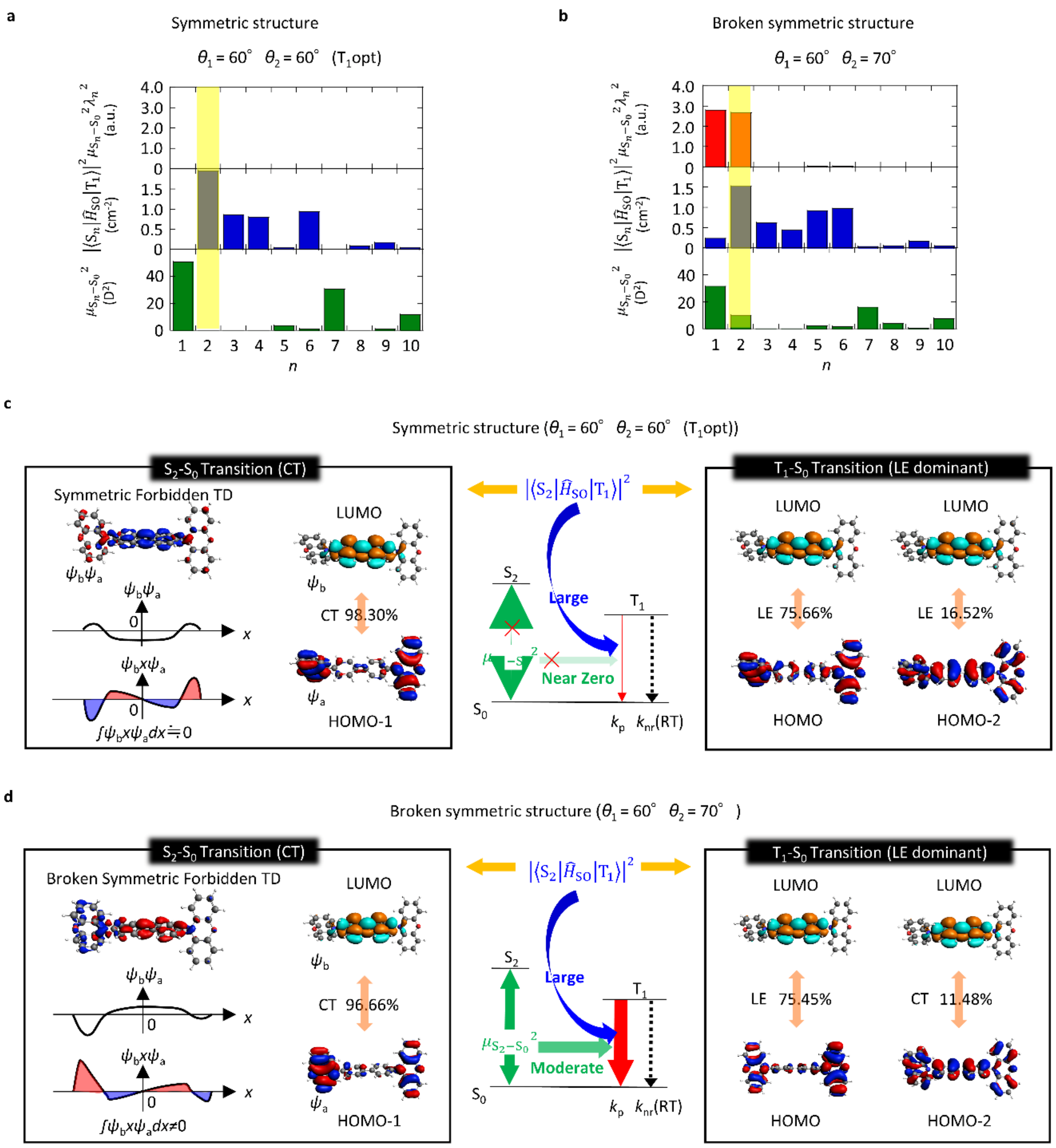

Figure S44. Visualization regarding the contribution of breaking the symmetry-forbidden transition to selective k_p enhancement in **4h.** (a–c) $\mu_{S_n-S_0}{}^2\lambda_n{}^2$ (i), $\langle S_n|\hat{H}_{SO}|T_1\rangle^2$ (ii), and $\mu_{S_n-S_0}{}^2$ (iii) for each n for a geometry in which $\theta_1 = \theta_2 = 60°$ (a) and a geometry in which $\theta_1 = 60°$ and $\theta_2 = 70°$ (c). (b and d) Schematic of molecular orbitals for k_p enhancement that is attributable to S_2–S_0 transitions for a geometry in which $\theta_1 = \theta_2 = 60°$ (b) and a geometry in which $\theta_1 = 60°$ and $\theta_2 = 70°$ (d).

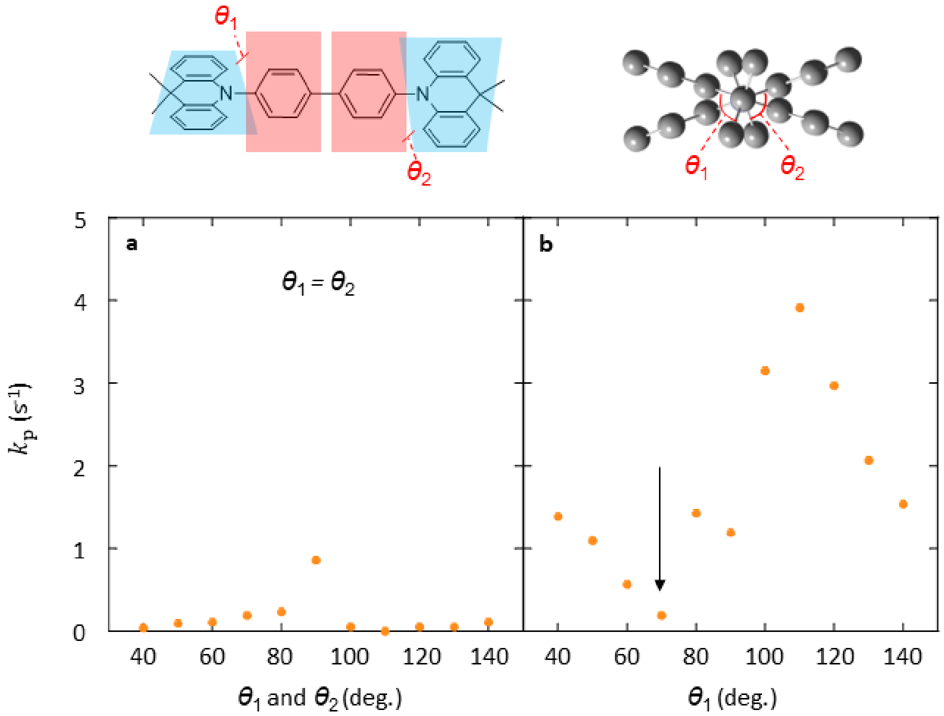

Figure S45. Contribution of the change of θ_1 and θ_2 considering the thermal energy to k_p of **5h**. The change of k_p when θ_1 and θ_2 were changed, yet $\theta_1 = \theta_2$ was kept (a); and the change of k_p when θ_1 was changed, yet $\theta_2 = 70°$ was kept (b).

Section S10. Ubiquitous afterglow readout

The visible light excited bright red afterglow emission (Φ_p = 21%, τ_p = 1.63 s) from 0.3 wt% chromophore **3d** doped in *β*-estradiol host was used for demonstrating the ubiquitous afterglow readout. We selected a red afterglow emitting dye **6d** (Φ_p = <2%, τ_p = 1.48 s) in comparison with **3d** (Figure 8c in main text). Initially, the melt-cast *β*-estradiol films doped with 0.3 wt% **3d** and **6d** were prepared at 240°C on a quartz substrate. The red emissive star ✶ pattern on films was prepared by exciting with a 360-nm laser (approximately 100 mW cm^{-2} for 10 min. Figure 8e in main text) in the presence of a solid ★ star photomask (blocks the UV light).[15] The ultraviolet (UV) light exposure led to photobleaching of the entire film except the star photomask part on the film (Figure 8c in the main text). Finally, two red afterglow emitting films doped with **3d** and **6d** were stacked (Figure 8c in the main text), and the afterglow emission was recorded by excitation with a flashlight under ambient conditions. Digital photographs and videos were captured with a Canon EOS digital camera. The ubiquitous afterglow readout under various conditions is indicated in Figure 8 and Movies S1–S3.

Section S11. Supporting movies

Movie S1. Demonstration of conventional afterglow readout in dark condition from a stacked sample prepared in Figure 8e soon after ceasing ultraviolet excitation.

Movie S2. Demonstration of white room light activated afterglow readout by naked eyes in dark condition from a stacked sample prepared in Figure 8e. The intensity of white room light is 0.21 mW cm^{-2} at the position of the sample when photon numbers of the white light is converted at 532 nm.

Movie S3. Demonstration of white LED-activated bright afterglow readout by naked eyes in white room light condition from a stacked sample prepared in Figure 8e. The intensity of white light excitation from the LED is 11.25 mW cm^{-2} at the sample position. The intensity of white room light is 0.094 mW cm^{-2} at the position of the sample when photon numbers of the white light is converted at 532 nm.

Section S12. References

[S1] Y. Fujii, Y. Taguchi, S. Tokai, Y. Matsumoto, N. Yoshida, T. Iwasawa, *Tetrahedron* **2021,** *95*, 132353.

[S2] M. M. Hossain, M. S. Mirzaei, S. V. Lindeman, S. Mirzaei, R. Rathore, *Org. Chem. Front.* **2021,** *8*, 2393.

[S3] I. Bhattacharjee, K. Hayashi, S. Hirata, *JACS Au* **2021**, *1*, 945.

[S4] S. Hirata, K. Totani, J. Zhang, T. Yamashita, H. Kaji, S. R. Marder, T. Watanabe, C. Adachi, *Adv. Funct. Mater.* **2013,** *23,* 3386.

[S5] O. V. Dolomanov, L. J. Bourhis, R. J. Gildea, J. A. K. Howard, H. Puschmann, *J. Appl. Cryst.* **2009,** *42*, 339.

[S6] T. Imagawa, S. Hirata, K. Totani, T. Watanabe, M. Vacha, *Chem. Commun.* **2015**, *61*, 13268.

[S7] H. D. Burrows, M. Fernandes, J. S. de Melo, A. P. Monkman, S. Navaratnam, *J. Am. Chem. Soc.* **2003,** *125*, 15310.

[S8] R. Huang, J. Avó, T. Northey, E. Chaning-Pearce, P. L. dos Santos, J. S. Ward, P. Data, M. K. Etherington, M. A. Fox, T. J. Penfold, M. N. Berberan-Santos, J. C. Lima, M. R. Bryce,F. B. Dias, *J. Mater. Chem. C* **2017**, *5*, 6269.

[S9] K. Fukasawa, K. Hiyashi, T. Yamashita, S. Hirata, *J. Phys. Chem. Lett.* **2022**, *13*, 1627.

[S10] A. Endo, K. Suzuki, , T. Yoshihara, S. Tobita, M. Yahiro, C. Adachi, *Chem. Phys. Lett.* **2008**, *460*, 155.

[S11] S. Hirata, *Appl. Phys. Rev.* **2022**, *9*, 011304.

[S12] E. W. Schlag, S. Schneider, S. F. Fischer, *Ann. Rev. Phys. Chem.* **1971**, *22*, 465.

[S13] S. Hirata, I. Bhattacharjee, *J. Phys. Chem.* A **2021**, *125*, 885.

[S14] K. Hayashi, K. Fukasawa, T. Yamashita, S. Hirata, *Chem. Mater.* **2022**, *34*, 1627.

[S15] B. Sk, R. Tsuru, K. Hayashi, S. Hirata, *Adv. Funct. Mater.* **2023**, *33*, 2211604.

[S16] S. Hirata, T. Kamatsuki, *J. Phys. Chem. C* **2023**, *127*, 3861.
